# Australian Assassins, Part I: A review of the Assassin Spiders (Araneae, Archaeidae) of mid-eastern Australia

**DOI:** 10.3897/zookeys.123.1448

**Published:** 2011-08-15

**Authors:** Michael G. Rix, Mark S. Harvey

**Affiliations:** 1 Department of Terrestrial Zoology, Western Australian Museum, Locked Bag 49, Welshpool DC, Perth, Western Australia 6986, Australia; 2 Research Associate, Division of Invertebrate Zoology, American Museum of Natural History, New York, NY 10024, USA; 3 Research Associate, California Academy of Sciences, 55 Music Concourse Drive, San Francisco, CA 94118, USA; 4 Adjunct Professor, School of Animal Biology, University of Western Australia, 35 Stirling Highway, Crawley, Perth, Western Australia 6009, Australia

**Keywords:** new species, taxonomy, rainforest, conservation, cytochrome c oxidase, mitochondrial DNA, Palpimanoidea

## Abstract

The Assassin Spiders of the family Archaeidae are an ancient and iconic lineage of basal araneomorph spiders, characterised by a specialised araneophagic ecology and unique, ‘pelican-like’ cephalic morphology. Found throughout the rainforests, wet sclerophyll forests and mesic heathlands of south-western, south-eastern and north-eastern Australia, the genus *Austrarchaea* Forster & Platnick, 1984 includes a diverse assemblage of relictual, largely short-range endemic species. With recent dedicated field surveys and significant advances in our understanding of archaeid biology and ecology, numerous new species of assassin spiders have been discovered in the montane sub-tropical and warm-temperate closed forests of mid-eastern Australia, including several rare or enigmatic taxa and species of conservation concern. This fauna is revised and 17 new species are described from south-eastern Queensland and eastern New South Wales: *Austrarchaea alani* **sp. n.**, *Austrarchaea aleenae* **sp. n.**, *Austrarchaea binfordae* **sp. n.**, *Austrarchaea christopheri* **sp. n.**, *Austrarchaea clyneae* **sp. n.**, *Austrarchaea cunninghami* **sp. n.**, *Austrarchaea dianneae* **sp. n.**, *Austrarchaea harmsi* **sp. n.**, *Austrarchaea helenae* **sp. n.**, *Austrarchaea judyae* **sp. n.**, *Austrarchaea mascordi* **sp. n.**, *Austrarchaea mcguiganae* **sp. n.**, *Austrarchaea milledgei* **sp. n.**, *Austrarchaea monteithi* **sp. n.**, *Austrarchaea platnickorum* **sp. n.**, *Austrarchaea raveni* **sp. n.** and *Austrarchaea smithae* **sp. n.** Adult specimens of the type species, *Austrarchaea nodosa* (Forster, 1956) are redescribed from the Lamington Plateau, south-eastern Queensland, and distinguished from the sympatric species *Austrarchaea dianneae* **sp. n.** A key to species and a molecular phylogenetic analysis of COI and COII mtDNA sequences complement the species-level taxonomy, with maps, habitat photos, natural history information and conservation assessments provided for all species.

## Introduction

**Figure 1.**
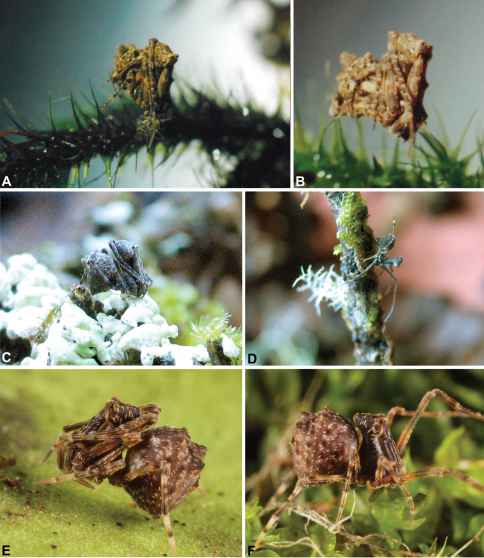
Habitus images of live Archaeidae from mid-eastern Australia: **A–B**, female *Austrarchaea nodosa* (Forster, 1956) from Binna Burra, Lamington National Park, Queensland; **C–D**, female *A. mascordi* sp. n. from Coolah Tops National Park, New South Wales; **E–F**, juvenile *A. raveni* sp. n. from Mount Glorious, Queensland. Images A–D by M. Rix; images E–F by Greg Anderson, used with permission.

The ‘assassin spiders’ of the family Archaeidae are an ancient and iconic lineage of basal araneomorph spiders, characterised by a remarkable cephalic morphology and specialised araneophagic ecology. Archaeid spiders are obligate predators of other spiders, and all possess a grossly-elevated, ‘pelican-like’ cephalothorax and long chelicerae (Figs 1, 4A-C) which are used to hunt and capture their spider prey (Legendre 1961, Forster and Platnick 1984, Wood et al. 2007, Wood 2008). With extant species known only from Australia, southern Africa and Madagascar, the family was first described in Europe from Baltic amber fossil specimens, prior to the discovery of living representatives in the forests of Madagascar in the mid-19th century (Cambridge 1881, Forster and Platnick 1984, Harvey 2002a, Wood et al. 2007). Other fossil assassin spiders – several congeneric with, and all remarkably similar to, extant taxa – have since been discovered in fossil strata of at least Mesozoic age, spectacularly illustrating the antiquity of the group (Penney 2003, Selden et al. 2008). Indeed, assassin spiders very similar to modern species were probably present throughout the Mesozoic; an observation further evidenced by recent higher-level phylogenetic research indicating the basal position of the Archaeidae relative to other araneomorph spider families (see Griswold et al. 2005, Rix et al. 2008, Rix and Harvey 2010).

**Figure 2.**
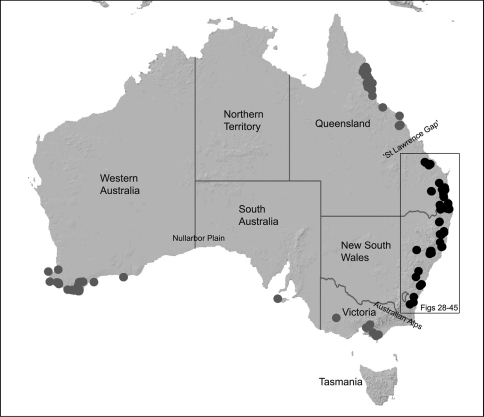
Map showing the known distribution of Archaeidae in Australia, with mid-eastern Australian localities highlighted in black. Note the absence of Archaeidae in central-eastern Queensland, the Australian Alps and Tasmania.

**Figure 3.**
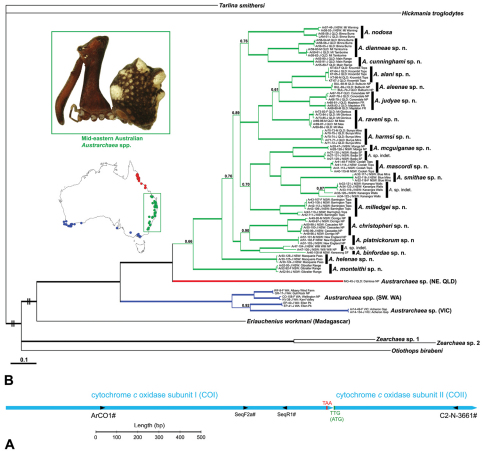
Molecular phylogenetic data analysed as part of this study. **A**, Schematic map of the mitochondrial cytochrome *c* oxidase subunit I–II (COI–COII) gene complex in Archaeidae and other basal Araneomorphae, showing (i) the position of primers used to amplify and sequence 1.6 kilobases of mtDNA, and (ii) the inferred stop and initiation codons for COI and COII, respectively. Note the centralised, overlapping position of the two internal sequencing primer sites (SeqF2a/SeqR1), and the TTG initiation codon for COII, present in all but one of the spider species sequenced for this study. **B**, Majority-rule consensus tree with re-estimated branch lengths, resulting from a combined, gene-partitioned Bayesian analysis of the COI–COII mtDNA data. Thickened branches represent clades with >95% posterior probability support, and individual support values are shown above other nodes.

Assassin spiders are iconic among arachnids due to the extraordinary history of their discovery, their remarkable appearance and antiquity, their limited distribution on the southern continents, their extreme endemism, and their highly specialised araneophagic biology (Forster and Platnick 1984, Harvey 2002a, Wood et al. 2007, Wood 2008). They are the emblem of Madagascar’s rich spider fauna (Wood 2008) and have attracted a great deal of research interest in recent years as highly diverse and endemic faunas have been uncovered in Madagascar and southern Africa (see Platnick 1991a, Lotz 1996, 2003, 2006, Wood et al. 2007, Wood 2008). The Australian fauna is comparatively poorly-known relative to those from the Malagasy and African regions, despite the presence of dozens of species in south-western, south-eastern and north-eastern Australia (Figs 2-3).

The Recent archaeid fauna consists of 37 described species in three genera (Platnick 2011): *Eriauchenius* O.P.-Cambridge, 1881 and *Afrarchaea* Forster & Platnick, 1984 from the Malagasy and African regions; and *Austrarchaea* Forster & Platnick, 1984, endemic to mainland Australia (Figs 1–2). Only five species of *Austrarchaea* have previously been described from opposite corners of continental Australia: *Austrarchaea daviesae* Forster & Platnick, 1984 from the Atherton Tableland, north-eastern Queensland; the type species *Austrarchaea nodosa* (Forster, 1956) from the Lamington Plateau, south-eastern Queensland; *Austrarchaea hickmani* (Butler, 1929) from Victoria; *Austrarchaea mainae* Platnick, 1991b from the Albany region of south-western Western Australia (see also Main 1995, Harvey 2002a, Rix and Harvey 2008); and *Austrarchaea robinsi* Harvey 2002a from the eastern Stirling Range National Park, south-western Western Australia. All five taxa were known only provisionally by their original taxonomic descriptions and subsequent collections, and recent research on *Austrarchaea* had not progressed beyond a simple recognition of the high levels of diversity and endemism present among Australian taxa (M. Rix, pers. obs.). In fact, the Australian archaeid fauna is far more diverse and widespread than expected even 10 years ago and, with recent advances in our understanding of archaeid biology and ecology, numerous new species and faunas have been discovered, including several species from regions previously assumed to be devoid of Archaeidae (e.g. southern South Australia and the south-eastern coast of Western Australia; see Fig. 2). In south-eastern Queensland and eastern New South Wales, the rainforests and montane wet sclerophyll forests along the Great Dividing Range provide habitats for at least 18 known species of *Austrarchaea*, most of which were undescribed, and all of which have relatively restricted, highly endemic distributions.

The current paper is thus a taxonomic revision of the species of Archaeidae known from ‘mid-eastern’ Australia, including those from south-eastern Queensland and eastern New South Wales, north of the Australian Alps (Fig. 2). The type species, *Austrarchaea nodosa*, is redescribed from the Lamington Plateau, south-eastern Queensland, and an additional 17 new species are described from habitats between Kroombit Tops National Park in Queensland and the Badja State Forest in southern New South Wales. These 18 species were found to form a monophyletic clade in a molecular phylogenetic analysis (Fig. 3B), and the remaining Australian Archaeidae will be described in forthcoming monographic treatments.

## Material and methods

All taxa were described and illustrated from specimens stored in 75% or 95% ethanol. Digital images were taken using a Leica MZ16A binocular microscope and a Leica DM2500 compound microscope, with auto-montage images captured using Leica DFC500 mounted cameras with Leica Application Suite Version 3.6.0 software. Male left pedipalps were dissected prior to imaging and bulbs were aligned for standardised comparison in the retrolateral and pro-distal positions illustrated; expanded pedipalps were illustrated in a retro-ventral position. Female genitalia were dissected and cleared in a 10% lactic acid plus 90% glycerol solution, prior to mounting on temporary glass slides. Illustrations were made on Utoplex tracing paper, using printed template auto-montage images.

**Measurements.** Measurements are in millimetres (rounded to the nearest hundredth of a millimetre) and were taken using an ocular graticule on a Leica M80 binocular microscope. Left legs were removed from specimens prior to taking measurements and imaging lateral body profiles. Lateral profile images were standardised for inter-specific comparison by vertically aligning the centre of each left anterior median eye with the lower anterior margin of the carapace (above the labrum) (Fig. 6). Carapace height was measured in lateral view, from the margin of the pars thoracica above coxa II to the highest point of the pars cephalica (Fig. 6). Carapace length was measured from the lower anterior margin of the carapace (above the labrum) to the posterior margin of the pars thoracica (above the pedicel) (Fig. 6). ‘Neck’ width was measured in lateral view, at the narrowest point of the carapace, with total length, carapace width, abdomen length and abdomen width all measured in dorsal view.

**Morphometrics.** To quantify inter-specific variation in the shape of the cephalothorax and ‘head’, three morphometric ratios were derived from lateral profile images (see Figs 6-9). The *carapace height to carapace length* (CH/CL) *ratio*, used extensively by Wood et al. (2007) and Wood (2008), quantifies the relative dorsal elevation of the carapace, irrespective of gross body size (Fig. 6). The CH/CL ratio used here differs slightly to that described by Wood (2008), in that carapace height and length are measured directly from relative points on the carapace (Fig. 6), and not necessarily at right angles to each other (see measurement definitions, above), thus avoiding any variation caused by tilting of the ‘neck’ or the non-perpendicular alignment of specimens. For any given size class, mid-eastern Australian *Austrarchaea* have a CH/CL ratio of 2.00–2.44; taxa with a relatively taller, more greatly elevated pars cephalica have a CH/CL ratio > 2.20 (Fig. 6). The *post-ocular ratio* (P.O. ratio) (Figs 7-9) measures the length of the ‘head’ posterior to the AME, relative to the dorsal elevation of the pars cephalica above the level of the AME, and quantifies the significant inter-specific (and often sexually dimorphic) variation seen in the relative dorsal extension of the posterior ‘head’ region (e.g. see Figs 8D, 8G) . While most species of *Austrarchaea* from mid-eastern Australia possess a post-ocular ratio of 0.25–0.35 (Figs 7J, 7N, 8H), several taxa possess a strongly elevated dorsal pars cephalica, with a P.O. ratio > 0.37 (Figs 7C, 8C, 8E, 9G). In contrast, the *highest point of pars cephalica* (HPC) *to post-ocular length ratio* (Figs 7–9) measures the position of the highest point of the dorsal pars cephalica, relative to the length of the ‘head’ posterior to the AME. It quantifies the equally significant variation observed in the position of the ‘head’ apex, from taxa with a more-or-less rounded or hemispherical ‘head’ in lateral view (HPC to post-ocular length ratio ~0.55–0.70) (Figs 7G, 7J, 7N) to taxa with a posteriorly extended, conical ‘head’ (HPC to post-ocular length ratio ~0.90) (Figs 7C, 8C-E).

**Molecular and laboratory methods.** For molecular analyses, specimens were preserved in 95% ethanol and stored at 4°C. Between two and seven legs of each individual were removed for DNA extractions and whole genomic DNA was extracted from leg tissue samples using the Qiagen DNeasy Blood and Tissue Kit protocol for animal tissues. Polymerase chain reaction (PCR) amplification of target gene regions was achieved using Invitrogen Platinum *Taq* Polymerase chemistry, in an Eppendorf Mastercycler ep gradient S thermal cycler. For each PCR reaction, 2 µl of extracted DNA, 0.25 µl of Platinum *Taq* (at 5 u/µl), 2 µl of MgCl2 (at 50 mM), 2.5 µl of 10x PCR buffer, 5 µl of dNTPs (at 1 mM) and 5 µl of each primer (at 2 µM) were used in every 25 µl reaction. For most taxa, 1071 bp of the mitochondrial cytochrome *c* oxidase subunit I (COI) gene, along with 535–541 bp of the adjacent COII gene (~1609 bp in total), were amplified using the primers ArCO1 (newly-designed for this study) and C2-N-3661b (modified from Simon et al. 1994), or variants thereof (see Tables 1–2). For several taxa, additional internal primers were used to amplify the same region in two overlapping segments. The PCR protocol used was: 94°C for 1 min; 35x (94°C for 30 sec, 52.1°C for 30 sec, 72°C for 1 min); 72°C for 5 min. The presence of PCR products in PCR reactions was confirmed using standard agarose gel electrophoresis; if PCR products were detected, PCR reactions were then purified using the MoBio UltraClean PCR Clean-up Kit. Bi-directional sequencing of purified PCR products was performed by Macrogen Corporation (South Korea), using supplied PCR primers and additional internal sequencing primers (see Table 1). Sequence (.ab1) files for the coding and non-coding strands were assembled automatically as anti-parallel contigs, and then visualised using Sequencher 4.8 (Demonstration Version). Annotated sequences were saved as text files, and imported into ClustalX Version 1.83 (Thompson et al. 1997) for alignment using default parameters.

**Conventions.** Throughout this paper the term ‘Border Ranges’ is used to denote the mountainous geographic border region between south-eastern Queensland and northern New South Wales (see Fig. 28B), including the McPherson Range and Main Range, and encompassing the World Heritage-listed rainforests of the Mount Warning, Border Ranges, Springbrook, Lamington, Mount Chinghee, Mount Barney, Mount Nothofagus, Mount Clunie, Koreelah and Main Range National Parks.

For Material Examined sections, specimens of indeterminate identification (usually juveniles) are included for mapping purposes, and tentatively linked to named species according to their geographic proximity to type localities (or in the case of genotyped juvenile specimens, according to their molecular phylogenetic affinity); such specimens are highlighted in Figures 28–45, and individually listed for each species. Specimens not examined for the current revision, but currently housed at the California Academy of Sciences (due to ongoing research) are also listed separately (with identifications confirmed by H. Wood), along with unexamined juvenile specimens recently accessioned. Specimens sequenced for the molecular analysis are denoted by superscript codes, which correspond to specimen codes as shown in Figure 3B and Table 2. For species Diagnoses, molecular autapomorphies (e.g. see Harvey et al. 2008, Cook et al. 2010) are coded according their nucleotide number (1–1609), as defined in Table 3 and Figure 3A.

Abbreviations used in the text are as follows:

ALEAnterior lateral eye/s

AMEAnterior median eye/s

CH/CLCarapace height (CH) to carapace length (CL) ratio

F1/CLFemur I length (F1) to carapace length (CL) ratio

HPCHighest point of pars cephalica

HT 1–6Abdominal hump-like tubercles 1–6

PMEPosterior median eye/s

TS 1–3Tegular sclerites 1–3

Specimens described in this study are lodged at the following institutions:

AMSAustralian Museum, Sydney (G. Milledge)

ANICAustralian National Insect Collection, Canberra (B. Halliday)

CASENTCalifornia Academy of Sciences, San Francisco (C. Griswold, D. Ubick)

MACNMuseo Argentino de Ciencias Naturales, Buenos Aires (M. Ramírez)

QMBQueensland Museum, Brisbane (R. Raven, O. Seeman)

WAMWestern Australian Museum, Perth (MSH, J. Waldock)

## Phylogenetic analysis

To complement and inform the morphological hypotheses presented for the species-level taxonomy (see below), and to provide molecular autapomorphies useful for distinguishing species of *Austrarchaea* from mid-eastern Australia, a molecular taxonomic approach was employed using mitochondrial DNA nucleotide sequences. A 1071 bp fragment of the cytochrome *c* oxidase subunit I (COI) gene, along with a 535–541 bp fragment of the adjacent COII gene (Fig. 3A, Table 3), were amplified in species of *Austrarchaea* (and outgroups) for analysis under a Bayesian framework. These data were generated and aligned as described in the Methods (above), and the resulting nexus file (see Appendix I) was analysed as highlighted (below).

**Taxa.** Specimens of Archaeidae were collected throughout mid-eastern Australia in March-May 2010, for use in molecular analyses. At least three specimens from each major population were sequenced for COI and COII; for some populations, fewer specimens were available. Most populations of Archaeidae previously known from mid-eastern Australia were successfully sampled and sequenced for the molecular analysis (see superscript DNA codes in the Material Examined sections, below), with numerous newly discovered populations also included. In total, sequences from 94 taxa were added to the final alignment (see Table 2), including 79 *Austrarchaea* from mid-eastern Australia, one archaeid specimen from north-eastern Queensland and eight Archaeidae from Victoria and Western Australia. A specimen of the Madagascan species *Eriauchenius workmani* O.P.-Cambridge, 1881 was also included, along with three other Palpimanoidea in the families Mecysmaucheniidae and Palpimanidae. The tree was rooted with the outgroups *Hickmania troglodytes* (Higgins & Petterd, 1883) (Austrochilidae) and *Tarlina smithersi* Gray, 1987 (Gradungulidae) (both in the superfamily Austrochiloidea), shown to be sister or basal to the Palpimanoidea in previous analyses (see Griswold et al. 2005, Rix et al. 2008, Rix and Harvey 2010).

**Analysis.** To infer phylogenetic relationships among sequenced specimens of Archaeidae from mid-eastern Australia, a combined, gene-partitioned Bayesian phylogenetic analysis was executed in MrBayes Version 3.1.2 (Huelsenbeck and Ronquist 2001, Ronquist and Huelsenbeck 2003). Prior to analysis, MrModeltest Version 3.7 (Posada and Crandall 1998) was used to choose the appropriate model of nucleotide substitution for each partition under an Akaike Information Criterion (AIC) framework; for the COI data, the GTR+I+G model was invoked with the following settings [Lset nst=6 rates=gamma]; for the COII data, the TVM+I+G model was invoked with the following settings [Lset nst=6 rates=gamma]. For each data partition, parameters were estimated independently ([Unlink tratio=(all) pinvar=(all) shape=(all) statefreq=(all) revmat=(all)]), rates were allowed to vary across partitions ([Prset applyto=(all) ratepr=variable]), and four Markov Chain Monte Carlo (MCMC) chains were run for 20 million generations, sampling every 1000 generations, with the final standard deviation of split frequencies < 0.01 and the first 2,000,000 sampled trees discarded as ‘burnin’ ([burnin=2000]). Burnin times and log likelikood trace files were visualised using Tracer Version 1.5 (Rambaut and Drummond 2009). Posterior probabilities were calculated and reported on a 50% majority-rule consensus tree of the post-burnin sample.

**Results and discussion.** The summary phylogenetic tree resulting from Bayesian analysis of the COI and COII data is presented in Figure 3B. The family Archaeidae and the genus *Austrarchaea* (as currently defined) were both monophyletic and strongly supported, with all mid-eastern Australian taxa similarly united in a monophyletic (although weakly supported) clade (highlighted green in Fig. 3B). Within this mid-eastern Australian lineage, evidence for at least 17 morphological species was supported by 17 equivalently-monophyletic and strongly supported molecular clades; inter-specific (i.e. sister-species) pairwise divergences for the combined (COI + COII) dataset ranged from 8–10%, with intra-specific divergences ranging from 0–6%. Three monophyletic clades from populations known only by juveniles (from the Kanangra-Boyd National Park, Willi Willi National Park and Badja State Forest) had sequence divergences in the range 8–9% (relative to sister-clades), suggesting that these populations may represent distinct species. Deeper species-group lineages were generally poorly supported by the COI and COII data, although *Austrarchaea monteithi* sp. n. was clearly inferred as a basal taxon, sister to all other species from mid-eastern Australia (Fig. 3B).

The results of the molecular phylogenetic analysis highlight the utility of comparing molecular and morphological taxonomic techniques, and provide a first insight into the possible phylogenetic relationships among Australian Archaeidae. Despite their exaggerated morphology and specialised ecology, species of *Austrarchaea* are otherwise morphologically conservative haplogyne spiders, with only relatively subtle inter-specific somatic and genitalic differences between adults, and a diagnostic requirement in most species for adult male specimens. This morphological conservatism, combined with the general paucity of specimens in collections, the relative over-representation of juveniles in collections and in the field, along with the difficulties associated with collecting adult males, renders the identification of species of *Austrarchaea* difficult based on morphology alone. By sequencing juveniles and adults from across mid-eastern Australia, a much clearer picture of the distribution and limits of each species has been achieved; populations known only from juveniles and females could be confidently linked to type localities, and newly-collected juvenile specimens could for the first time be associated with conspecific adult specimens. In the case of collections made at Binna Burra (Lamington National Park) in April 2010, juvenile specimens of two sympatric species were successfully genotyped to determine their identification, and to test whether *Austrarchaea nodosa* and *Austrarchaea dianneae* sp. n. were truly sympatric on the Lamington Plateau (see Nomenclatural Remarks for *Austrarchaea nodosa*, below).

The phylogenetic relationships inferred for Australian species of Archaeidae remain highly preliminary in the absence of additional genes and a greater taxon sample from southern and north-eastern Australia (M. Rix, unpublished data), however several key results are worthy of discussion. Firstly, the enigmatic *Austrarchaea monteithi* sp. n., from the Gibraltar Range National Park (Fig. 19), was clearly inferred as a basal sister-species to all other Archaeidae from mid-eastern Australia, which together formed a monophyletic (although weakly supported) mid-eastern Australian clade (highlighted green in Fig. 3B) sister to an undescribed species from north-eastern Queensland. This result is congruent with morphology, in that the linear gradation seen in the number of dorsal hump-like tubercles on the abdomen (four in north-eastern Queensland taxa; five in *Austrarchaea monteithi* sp. n.; six in all other mid-eastern Australian taxa; Figs 5E-G) matches the inferred gradation of clades in Figure 3B. Similarly, the observed gap in the distribution of archaeid species in central Queensland, roughly consistent with the ‘St Lawrence Gap’ (Webb and Tracey 1981) between Gladstone and Mackay (Fig. 2), seems to reflect a genuine phylogenetic barrier, rather than a collecting artefact. The other major gap in the distribution of Archaeidae in mesic eastern Australia, roughly consistent with the mountainous Australian alpine zone bordering New South Wales and Victoria (Fig. 2), seems to also reflect a second major phylogenetic barrier between a divergent clade of southern Australian taxa (highlighted blue in Fig. 3B) and all other Australian Archaeidae.

Clearly, applying molecular taxonomic methods to a morphological taxonomy is of great utility for species of *Austrarchaea*. For the current revision, molecular data are clearly linked to specimens and to museum registration numbers by using DNA taxon codes in Material Examined sections, each of which corresponds to an equivalent code in Table 2, and to branch terminals in Figure 3B. To fully integrate the molecular data with the morphological taxonomic hypotheses presented (below), species are also diagnosed (where possible) with unique molecular autapomorphies, in addition to standard morphological characters (see Conventions, above). This approach will facilitate the molecular identification of specimens in the future (as advocated by numerous authors, e.g. Cook et al. 2010), and assist in accurately genotyping juveniles for which genitalic and adult somatic characters are unavailable.

**Table 1.** Primers used to amplify and sequence COI and COII genes for the molecular analysis. Underlined letters denote nucleotide modifications.

Name	Sequence (5’-3’)	Type (Gene)	References
**PCR PRIMERS**			
**ArCO1**	CATTTAGCTGGTGCTTCTTCTATT	Forward (COI)	
ArCO1a1	CATTTAGCTGGTGCTTCATCTATT	Forward (COI)	
ArCO1c2	CATTTGGCTGGGGCGTCATCAATT	Forward (COI)	
ZrCO13	TCTTTACATTTAGCTGGTGCTTCTT	Forward (COI)	
**C2-N-3661**	CACAAATTTCTGAACATTGACCA	Reverse (COII)	Simon et al. (1994)
C2-N-3661a4	CACAAATTTCAGAACATTGACCA	Reverse (COII)	Simon et al. (1994)
C2-N-3661b5	CACAAATTTCAGAACATTGACCT	Reverse (COII)	Simon et al. (1994)
SEQUENCING/PCR PRIMERS*			
**SeqF2a**	TYCATTATGTWTTAAGAATAGG	Forward (COI)	
SeqF2a1	CATTTYCATTATGTDTTRAGAATRGG	Forward (COI)	
**SeqR1**	CATCAGGATAATCWGAATAHCG	Reverse (COI)	
SeqR1a	CATCWGGRTARTCHGAATAHCGACG	Reverse (COI)	

* Used as PCR primers in certain taxa (see Table 2)

1 Used for Victorian and Stirling Range National Park *Austrarchaea* spp.

2 Used for *Otiothops birabeni*

3 Used for *Zearchaea* sp. 2

4 Used for outgroups and Western Australian/Victorian *Austrarchaea* spp.

5 Used for most south-eastern Australian *Austrarchaea* spp.

**Table 2.** Specimens sequenced for the molecular analysis. Primer sequences are further listed in Table 1.

Species/Museum No.	SpecimenCode	GenBankAccession	Forward PCRPrimer/s	Reverse PCRPrimer/s
**OUTGROUPS**				
*Hickmania troglodytes* (Higgins & Petterd, 1883) (Bubs Hill Karst, TAS):				
WAM T79989	N.A.	**JF909360**	ArCO1	C2-N-3661a
*Tarlina smithersi* Gray, 1987 in Forster et al., 1987 (Willi Willi National Park, NSW):				
WAM T112581	N.A.	**JF909361**	ArCOI/SeqF2a	SeqR1/C2-N-3661a
*Otiothops birabeni* Mello-Leitão, 1945 (Parque Nacional El Palmar, Argentina):				
MACN Ar11491	N.A.	**JF909362**	ArCO1c/SeqF2a	SeqR1/C2-N-3661a
*Zearchaea* sp. 1 (Lewis Pass, New Zealand):				
WAM T79990	N.A.	**JF909363**	ArCO1/SeqF2a	SeqR1/C2-N-3661a
*Zearchaea* sp. 2 (Milford Sound, New Zealand):				
WAM T112582	N.A.	**JF909364**	ZrCO1	C2-N-3661a
*Eriauchenius workmani* O.P.-Cambridge, 1881 (Ranomafana, Madagascar):				
CASENT 9018984	N.A.	**JF909365**	ArCO1	C2-N-3661a
**OTHER *AUSTRARCHAEA* SPP.**				
*Austrarchaea* sp. (Acheron Gap, VIC):				
WAM T112583	Ar14-49-F	**JF909366**	ArCO1a	C2-N-3661a
WAM T112583	Ar14-134-J	**JF909367**	ArCO1a	C2-N-3661a
*Austrarchaea mainae* Platnick, 1991b (Albany, SW. WA):				
WAM T89572	WF-9-F	**JF909368**	ArCO1	C2-N-3661a
WAM T89578	GR-17-J	**JF909369**	ArCO1	C2-N-3661a
*Austrarchaea robinsi* Harvey, 2002a (Stirling Range, SW. WA):				
WAM T89558	EP-40-J	**JF909370**	ArCO1a	C2-N-3661a
WAM T89558	EP-41-J	**JF909371**	ArCO1a	C2-N-3661a
*Austrarchaea* sp. (Karri Valley, SW. WA):				
WAM T89565	KV-38-J	**JF909372**	ArCO1	C2-N-3661a
*Austrarchaea* sp. (Wellington National Park, SW. WA):				
WAM T112584	CO-158-F	**JF909373**	ArCO1	C2-N-3661a
*Austrarchaea* sp. (Daintree National Park, NE. QLD):				
WAM T97462	MG-45-J	**JF909374**	ArCO1	C2-N-3661
**MID-EASTERN AUSTRALIAN *AUSTRARCHAEA* SPP.**				
*Austrarchaea nodosa* (Forster, 1956):				
WAM T89592	LAM-51-J	**JF909375**	ArCO1/SeqF2a1	SeqR1a/C2-N-3661
WAM T112571	Ar56-58-J	**JF909376**	ArCO1	C2-N-3661b
WAM T112572	Ar57-46-J	**JF909377**	ArCO1	C2-N-3661b
WAM T112573	Ar58-53-J	**JF909378**	ArCO1	C2-N-3661b
*Austrarchaea alani* sp. n.:				
WAM T112550	KT-63-F	**JF909379**	ArCO1	C2-N-3661b
WAM T112550	KT-64-J	**JF909380**	ArCO1	C2-N-3661b
WAM T112550	KT-65-J	**JF909381**	ArCO1	C2-N-3661b
WAM T112551	KT-66-M	**JF909382**	ArCO1	C2-N-3661b
WAM T112551	KT-67-J	**JF909383**	ArCO1	C2-N-3661b
*Austrarchaea aleenae* sp. n.:				
WAM T112552	BUL-68-M	**JF909384**	ArCO1/SeqF2a1	SeqR1a/C2-N-3661b
WAM T112552	BUL-69-J	**JF909385**	ArCO1/SeqF2a1	SeqR1a/C2-N-3661b
WAM T112552	BUL-70-J	**JF909386**	ArCO1/SeqF2a1	SeqR1a/C2-N-3661b
*Austrarchaea binfordae* sp. n.:				
AMS KS114969	Ar46-106-M	**JF909402**	ArCO1	C2-N-3661b
*Austrarchaea christopheri* sp. n.:				
AMS KS114968	Ar49-95-M	**JF909387**	ArCO1	C2-N-3661b
WAM T112554	Ar49-96-J	**JF909388**	ArCO1	C2-N-3661b
WAM T112554	Ar49-97-J	**JF909389**	ArCO1	C2-N-3661b
WAM T112553	Ar50-98-J	**JF909390**	ArCO1	C2-N-3661b
WAM T112553	Ar50-99-J	**JF909391**	ArCO1	C2-N-3661b
WAM T112553	Ar50-100-J	**JF909392**	ArCO1	C2-N-3661b
*Austrarchaea cunninghami* sp. n.:				
WAM T112555	Ar55-89-F	**JF909393**	ArCO1	C2-N-3661b
WAM T112555	Ar55-90-J	**JF909394**	ArCO1	C2-N-3661b
WAM T112555	Ar55-91-J	**JF909395**	ArCO1	C2-N-3661b
*Austrarchaea dianneae* sp. n.:				
WAM T112557	Ar59-60-M	**JF909396**	ArCO1	C2-N-3661b
WAM T112557	Ar59-61-J	**JF909397**	ArCO1	C2-N-3661b
WAM T112557	Ar59-62-J	**JF909398**	ArCO1	C2-N-3661b
WAM T112556	Ar56-54-M	**JF909399**	ArCO1	C2-N-3661b
WAM T112556	Ar56-55-J	**JF909400**	ArCO1	C2-N-3661b
WAM T112556	Ar56-56-J	**JF909401**	ArCO1	C2-N-3661b
*Austrarchaea harmsi* sp. n.:				
WAM T112559	Ar70-73-M	**JF909406**	ArCO1/SeqF2a1	SeqR1a/C2-N-3661b
WAM T112559	Ar70-74-J	**JF909407**	ArCO1/SeqF2a1	SeqR1a/C2-N-3661b
WAM T112559	Ar70-75-J	**JF909408**	ArCO1/SeqF2a1	SeqR1a/C2-N-3661b
WAM T112560	Ar71-71-J	**JF909409**	ArCO1/SeqF2a1	SeqR1a/C2-N-3661b
WAM T112560	Ar71-72-J	**JF909410**	ArCO1/SeqF2a1	SeqR1a/C2-N-3661b
*Austrarchaea helenae* sp. n.:				
WAM T112561	Ar30-124-J	**JF909411**	ArCO1	C2-N-3661b
WAM T112561	Ar30-125-J	**JF909412**	ArCO1	C2-N-3661b
WAM T112561	Ar30-126-J	**JF909413**	ArCO1	C2-N-3661b
*Austrarchaea judyae* sp. n.:				
WAM T112563	Ar67-76-F	**JF909414**	ArCO1	C2-N-3661b
WAM T112563	Ar67-78-J	**JF909415**	ArCO1	C2-N-3661b
WAM T112562	Ar66-79-J	**JF909416**	ArCO1	C2-N-3661b
WAM T112564	Ar68-80-M	**JF909417**	ArCO1	C2-N-3661b
WAM T112564	Ar68-81-J	**JF909418**	ArCO1	C2-N-3661b
WAM T112564	Ar68-82-J	**JF909419**	ArCO1	C2-N-3661b
*Austrarchaea mascordi* sp. n.:				
AMS KS114973	Ar41-48-F	**JF909420**	ArCO1	C2-N-3661b
WAM T112566	Ar41-113-J	**JF909421**	ArCO1	C2-N-3661b
WAM T112566	Ar41-114-J	**JF909422**	ArCO1	C2-N-3661b
WAM T112565	Ar40-115-M	**JF909423**	ArCO1	C2-N-3661b
*Austrarchaea mcguiganae* sp. n.:				
WAM T112567	Ar28-47-J	**JF909424**	ArCO1	C2-N-3661
WAM T112567	Ar28-128-J	**JF909425**	ArCO1	SeqR1A/C2-N-3661
*Austrarchaea milledgei* sp. n.:				
WAM T112568	Ar43-107-F	**JF909426**	ArCO1	C2-N-3661b
WAM T112568	Ar43-108-J	**JF909427**	ArCO1	C2-N-3661b
WAM T112568	Ar43-109-J	**JF909428**	ArCO1	C2-N-3661b
WAM T112569	Ar42-110-J	**JF909429**	ArCO1	C2-N-3661b
WAM T112569	Ar42-111-J	**JF909430**	ArCO1	C2-N-3661b
WAM T112569	Ar42-112-J	**JF909431**	ArCO1	C2-N-3661b
*Austrarchaea monteithi* sp. n.:				
AMS KS114976	Ar52-92-F	**JF909432**	ArCO1	C2-N-3661b
WAM T112570	Ar52-93-J	**JF909433**	ArCO1	C2-N-3661b
WAM T112570	Ar52-94-J	**JF909434**	ArCO1	C2-N-3661b
*Austrarchaea platnickorum* sp. n.:				
WAM T112558	Ar51-101-M	**JF909403**	ArCO1	C2-N-3661b
WAM T112558	Ar51-102-F	**JF909404**	ArCO1	C2-N-3661b
WAM T112558	Ar51-103-J	**JF909405**	ArCO1	C2-N-3661b
*Austrarchaea raveni* sp. n.:				
QMB S90192	Ar73-83-F	**JF909435**	ArCO1/SeqF2a	SeqR1/C2-N-3661b
WAM T112574	Ar73-84-J	**JF909436**	ArCO1/SeqF2a	SeqR1/C2-N-3661b
WAM T112574	Ar73-85-J	**JF909437**	ArCO1/SeqF2a	SeqR1/C2-N-3661b
WAM T112575	Ar69-86-M	**JF909438**	ArCO1/SeqF2a	SeqR1/C2-N-3661b
WAM T112575	Ar69-87-J	**JF909439**	ArCO1/SeqF2a	SeqR1/C2-N-3661b
WAM T112575	Ar69-88-J	**JF909440**	ArCO1/SeqF2a	SeqR1/C2-N-3661b
*Austrarchaea smithae* sp. n.:				
WAM T112576	Ar32-116-F	**JF909441**	ArCO1	C2-N-3661b
WAM T112576	Ar32-117-J	**JF909442**	ArCO1	C2-N-3661b
WAM T112576	Ar32-118-J	**JF909443**	ArCO1	C2-N-3661b
*Austrarchaea* sp. indet. (Willi Willi National Park, NSW):				
WAM T112580	Ar47-104-J	**JF909444**	ArCO1	C2-N-3661b
WAM T112580	Ar47-105-J	**JF909445**	ArCO1	C2-N-3661b
*Austrarchaea* sp. indet. (Kanangra-Boyd National Park, NSW):				
WAM T112578	Ar33-119-J	**JF909446**	ArCO1	C2-N-3661b
WAM T112578	Ar33-120-J	**JF909447**	ArCO1	C2-N-3661b
WAM T112578	Ar33-121-J	**JF909448**	ArCO1	C2-N-3661b
WAM T112579	Ar34-122-J	**JF909449**	ArCO1	C2-N-3661b
WAM T112579	Ar34-123-J	**JF909450**	ArCO1	C2-N-3661b
*Austrarchaea* sp. indet. (Badja State Forest, NSW):				
WAM T112577	Ar27-129-J	**JF909451**	ArCO1	C2-N-3661b
WAM T112577	Ar27-130-J	**JF909452**	ArCO1	C2-N-3661b
WAM T112577	Ar27-131-J	**JF909453**	ArCO1	C2-N-3661b

**Table 3.** Mitochondrial COI-COII DNA sequence of juvenile *Austrarchaea nodosa* (Forster, 1956) (WAM T112571), showing the nucleotide numbering system (1-1609) used to designate molecular autapomorphies for species diagnoses. Underlined nucleotides denote stop and initiation codons for COI and COII, respectively.

COI mtDNA (nucleotides 1-1071)
ATAGGTGCTGTAAATTTTATTTCTACTATTTTGAATATACGATCTTATGGAATGAGAATAGATAAAGTTCCTTTGTTTGTTTGGTCTGTATTAATTACAGCTATTTTATTACTATTATCTTTGCCTGTTTTAGCTGGGGCAATTACAATATTGTTAACAGATCGAAATTTTAATACTTCTTTCTTTGATCCTGCGGGAGGTGGGGATCCTATTTTATTTCAACATTTATTTTGATTTTTTGGTCACCCTGAAGTTTATATTTTAATTTTACCTGGTTTTGGTATTGTTTCTCATGTTATTAGAGGATCAGTAGGTAAGCGTGAGCCTTTTGGTAGATTGGGGATGATTTATGCTATAGTTGGAATTGGTGGGATAGGGTTTGTTGTATGAGCCCATCATATATTTTCTGTTGGAATGGATGTGGATACTCGGGCGTATTTTACTGCTGCTACTATAATTATTGCAGTTCCCACTGGAATTAGGGTATTTAGATGGATAGCTACTTTATATGGGTCTTATTTTAAATTGGAAGCTCCATTATTATGATGTGTGGGATTTGTGTTTTTATTTACTTTAGGCGGGGTTACAGGAGTAGTTTTAGCTAATTCTTCTTTAGATATTGTTTTACATGATACTTACTATGTGGTTGCTCATTTTCATTATGTGTTAAGTATAGGAGCTGTATTTGCTATTTTGGCTGGTATTACTTATTGATTTCCTTTGTTTTTTGGGGTAGTTCTGAATTCAAGGAATTCTAATTTACAATTTTTTATTATATTTATTGGAGTGAATTTAACTTTTTTTCCTCAACATTTTTTGGGGTTAAATGGTATACCACGTCGTTATTCTGATTATCCTGACGCTTTTATTTACTGAAATATAGTTTCTTCTTTAGGGTCTTTATTATCTTTATTAGGAATTTTATTTTTTATATTAATCATTTGAGATGGATTTATTTCAAAAAATTTAGGATTTTCGAATTATTATATATATTCTTCGTTGGAGTGAAATAATGGAGTTCCCCCATTAGATCATACATTTAATCAGTTAGGACAATTGAATATTTAATTT
COII mtDNA (nucleotides 1072-1609)
**TTG**CCAACTTGAGGTTCATTGTATTTTCAAAATAGTTCTTCTTTTGTTATGGAGCAGTTAATTTTTTTTCATGATTATACAATGGTAATTTTGATTATGATTATAGTTATTGTGGGGTATTTATTAGTGAATTCTTGTTATGAAAATTATTATAATCACATGTTAAATGAGGGTCAAGAGTTAGAGAGAATTTGGACTGTTCTTCCAGCTTTATTTTTGTTATTAATTGCTTTTCCTTCTTTACAATTGTTATATTTAATAGAGGAAATAGAATTTCCTGAATTAACTATTAAAATTTTAGGTCATCAGTGATATTGATCTTATGAGTATAGAGATATAGGTTTAGATTCGTTTGAGTCTTATATAATCAGAGGGGGGAGTGTACTTTTACGGCTTTTAGAGGTTGATAATAATTTAGTGATCCCTTATAATTCTATTACTCGTATAATTATTTCTAGAAGAGATGTTATTCATTCTTGAACTATTCCGTCTTTAGGTGTAAAAATAGATGCTATTCCAGGTCGATTAAACCAAATTT

## Taxonomy

**Family Archaeidae Koch & Berendt, 1854**

### Genus Austrarchaea

Forster & Platnick, 1984

http://species-id.net/wiki/Austrarchaea

Austrarchaea Forster & Platnick, 1984: 21; Platnick 1991b: 259; Main 1995: 151; Harvey 2002a: 35.

#### Type species.

*Archaea nodosa* Forster, 1956, by original designation.

#### Diagnosis.

**Figure 4.**
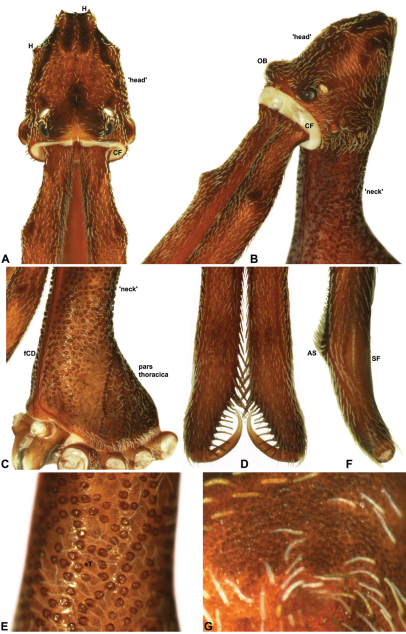
Carapace morphology of *Austrarchaea *species. **A–E**, *A. alani* sp. n.: **A**, male pars cephalica, frontal view, showing dorsal ‘head’ region, posterior horns (H) and cheliceral foramen (CF); **B**, female pars cephalica, antero-lateral view, showing ocular bulge (OB), cheliceral foramen (CF) and division of pars cephalica into ‘head’ and ‘neck’ regions; **C**, male pars thoracica, ‘neck’ and fused cheliceral diastema (fCD), antero-lateral view; **D**, female chelicerae and peg teeth, frontal view; **E**, male ‘neck’, lateral view, showing setose tubercles (sT). **F–G**, *A. judyae* sp. n.: **F**, male chelicerae, lateral view, showing accessory setae (AS) and ectal stridulatory file (SF); **G**, detail of female posterior pars cephalica, lateral view, showing field of densely granulate cuticle.

**Figure 5.**
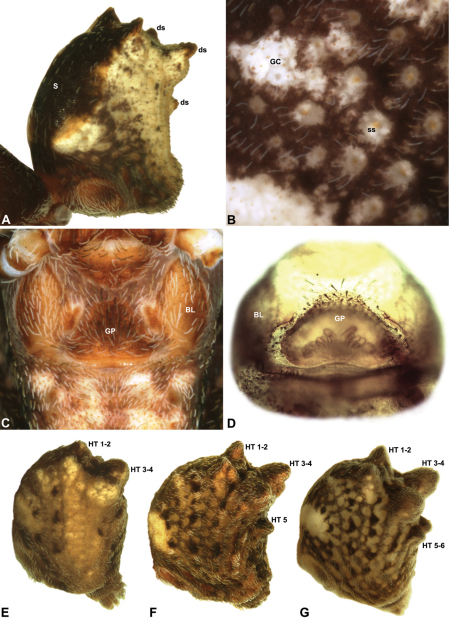
Abdominal morphology of *Austrarchaea *species. **A–C**, *A. judyae* sp. n.: **A**, male abdomen, antero-lateral view, showing dorsal scute (S) and additional dorsal sclerites (ds); **B**, detail of female abdomen, lateral view, showing subcuticular guanine crystals (GC) and concentric arrangements of setae around sclerotic spots (ss); **C**, female epigastric region, ventral view, showing setose book lung covers (BL) and genital plate (GP). **D**, Cleared epigastric region of female *A. nodosa* (Forster), postero-ventral view, showing position of clustered spermathecae under posterior rim of genital plate. **E–G**, Female abdomens, postero-lateral view, showing arrangement of dorsal hump-like tubercles (HT) in different taxa: **E**, *A*. sp. nr. *daviesae* (QMB S72989, from Mount Bartle Frere, NE. Queensland); **F**, *A. monteithi* sp. n.; **G**, *A. aleenae* sp. n. Note the presence of only a single posterior hump-like tubercle (HT 5) in *A. monteithi*.

Species of *Austrarchaea* can be distinguished from all other extant Archaeidae (i.e. Malagasy and African species of *Eriauchenius* and *Afrarchaea*) by the presence of numerous, clustered spermathecae in females (Figs 5D, 10G, 14G) and by the presence of a long, wiry embolus on the pedipalp of males (Figs 10E, 15E, 27E) (Forster and Platnick 1984, Wood 2008). The remarkable, elevated shape of the carapace (Figs 4A-C, 10A-B) and the very long chelicerae (Figs 4B, 4D) will also immediately separate this genus from all other Australian spiders.

#### Description.

Small, haplogyne, araneomorph spiders; total length 2.5 to 5.0.

*Colouration*: Body colouration cryptic and relatively uniform across species, usually with only subtle intraspecific variation in abdominal patterning; carapace, sternum and chelicerae tan brown to dark reddish-brown, interspersed with darker regions of granulate cuticle (Fig. 5), covered in highly reflective setae; legs tan-brown to darker reddish-brown, with pattern of darker annulations on distal segments; abdomen mottled with beige and variable hues of grey-brown (Figs 5E-G), with darker sclerites, scutes and sclerotic spots (Figs 5A-B); paler beige markings due to reflective, subcuticular guanine crystals (Fig. 5B); antero-lateral face of abdomen always with large, humeral patch of reflective guanine crystals (Figs 5A, 5E-G).

**Figure 6.**
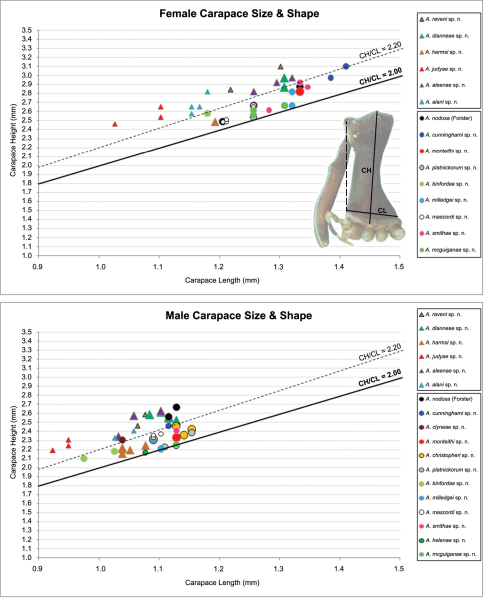
Graphs depicting the relationship between carapace length (CL) and carapace height (CH) for species of *Austrarchaea* from mid-eastern Australia. Overall body size variation is quantified by the relative lengths of the carapace, whereas carapace shape variation is reflected by the CH/CL ratio; taxa with a very tall, greatly elevated pars cephalica have a CH/CL ratio > 2.20. Circles ● denote New South Wales and southern Queensland species; and triangles ▲ denote Queensland species (from north of the Border Ranges). Note the relatively small body sizes of *A. judyae *sp. n., *A. binfordae* sp. n. and *A. alani *sp. n., and the relatively tall carapaces of most Queensland taxa. Note also the smaller body sizes and lower variance in carapace length among males relative to females.

*Cephalothorax*: Carapace greatly elevated anteriorly (CH/CL ratio usually 2.0–2.4; Fig. 6), with raised, highly modified pars cephalica forming ‘neck’ and bulbous ‘head’ (see Wood 2008) (Figs 4A-C); ‘neck’ with concomitantly long diastema (see Schütt 2002) between cheliceral bases and anterior margin of carapace, fused along entire length with sclerotised cuticle (Fig. 4C); cheliceral bases emanating from broad, fully-enclosed cheliceral foramen situated at front of ‘head’ (Figs 4A-B); posterior ‘head’ region usually also bearing two pairs of rudimentary protrusions or ‘horns’, each typically terminating in a short, thickened seta (Fig. 4A). Carapace with densely granulate cuticular microstructure (Fig. 4G), covered in larger setose tubercles arranged in clusters or distinct rows (Figs 4C, 4E); each tubercle bearing single densely plumose or ciliate seta; setose tubercles largest on ‘neck’ and pars thoracica (Figs 4C, 4E). Eight eyes present on anterior margin of ‘head’, in four widely separated diads (Figs 4A-B); AME largest, widely separated, directed antero-laterally on rounded ocular bulge (Fig. 4B); PME situated closely posterior to AME, directed obliquely on postero-lateral side of ocular bulge; lateral eyes contiguous, with shared raised bases, directed ventro-laterally on widest lateral margin of ‘head’ (Figs 4A-B). Sternum longer than wide, covered in setose tubercles; lateral margins separated from dorsal pleural sclerite extending between coxae I-IV. Labium subtriangular, not fused to sternum, directed antero-ventrally at oblique angle to sternum; labrum with pair of divergent projections on anterior surface. Maxillae large (Fig. 4C), straddling labium and labrum, converging distally; serrula a single row of teeth. Chelicerae very long, spear-like, distally divergent (Figs 4B, 4D, 4F), usually with proximal bulging projection (Fig. 4B); both sexes with oval, ectal stridulatory file adjacent to pedipalps (Fig. 4F); males usually also with brush (Figs 4F, 12C, 19C, 22C), short comb (Figs 14C, 18C) or dense tuft (Figs 16C, 17C) of accessory setae on anterior face of paturon. Chelicerae armed with three rows of peg teeth; anterior (prolateral) row with two peg teeth near tip of fang; posterior (retrolateral) row with single peg tooth near tip of fang; median (prolateral) row with more than 15 peg teeth extending along inner prolateral margin of paturon to near base of fang; median row with approximately nine porrect, comb-like peg teeth adjacent to fang, several larger, flattened, spiniform peg teeth near tip of fang, and additional progressively shorter, spiniform peg teeth along inner paturon (Fig. 4D); cheliceral retromargin also with four or five true teeth and prominent cheliceral gland mound.

*Legs and female pedipalp*: Legs (longest to shortest) 1–4–2–3, covered with short plumose setae; spines absent; patella I long, greater than one-third length of femur I. Trichobothria present on tibiae and metatarsi of legs; tibiae I-IV each with two trichobothria; metatarsi I-IV each with single trichobothrium; bothrial bases with strongly ridged hood. Tarsi shorter than metatarsi, with capsulate tarsal organ and three claws; tarsi, metatarsi and distal tibiae of legs I-II usually with ventral and pro-ventral rows of moveable, spatulate setae. Female pedipalp with long, porrect trochanter and small tarsal claw; tibia with two dorsal trichobothria.

*Abdomen*: Abdomen arched anteriorly, rounded-subtriangular in lateral view, usually with four to six large hump-like tubercles on dorsal surface (Figs 5A, 5E-G); cuticle covered with short plumose setae and numerous sclerotic spots (Figs 5A-B). Epigastric region with sclerotised (setose) book lung covers and dorsal and ventral plates surrounding pedicel (Fig. 5C) (plates fused in males); dorsal pedicel plate with transverse ridges; females with median genital plate and sclerotised lateral sigillae (Figs 5C-D); males with broad dorsal scute fused anteriorly to epigastric sclerites, with or without additional paired sclerites associated with hump-like tubercles (Fig. 5A). Six spinnerets, surrounded by thickened cuticle; ALS largest, PMS smallest; colulus absent. Posterior pair of divided tracheal spiracles situated anterior to spinnerets; males also with transverse row of epiandrous gland spigots situated closely anterior to epigastric furrow.

*Genitalia*: Female genitalia haplogyne, with sclerotised, strongly arched genital plate anterior to epigastric furrow (Figs 5C-D); internally with gonopore leading to large, spherical membranous bursa (Fig. 17G; see also Forster and Platnick 1984, fig. 57) overlying two separate, radiating clusters of sclerotised anterior spermathecae (Figs 5D, 10G, 14G, 19G). Male pedipalp with complex, expandable pyriform bulb (Figs 10E, 19E, 23E, 24E), consisting of smooth tegulum, proximal ‘subtegulum’ and associated tegular groove with basal haematodocha (Figs 10E, 23E, 27E) (similar to Mecysmaucheniidae and potentially analogous to the subtegular division of Entelegynae); distal tegulum with excavate, rimmed cavity surrounding massive, inflatable haematodochal complex incorporating distal embolus, basal embolic sclerite and multiple tegular sclerites (Figs 26D, 27E) (see below); distal haematodochal complex with balloon-like proximal portion (anchored by distal rim of tegulum) and sinuous, tapering embolic portion (anchored by flexible, hinged retro-ventral conductor) (Figs 26D, 27E). Unexpanded pedipalp with folded, wiry embolus abutting conductor (Fig. 17E); tegular sclerites embedded pro-distally (Fig. 20E); pedipalpal expansion and haematodochal inflation (e.g. see Figs 14E, 23E, 26D, 27E) resulting in significant conformational changes to shape of conductor, length and orientation of embolus, and relative position of tegular sclerites.

As noted by Wood (2008), the homology of the tegular sclerites among archaeid genera remains unclear, and this is especially true for *Austrarchaea* relative to Malagasy and African taxa. For the purposes of this revision, and for an easy comparison among species of *Austrarchaea* from mid-eastern Australia, the moveable tegular sclerites of the pedipalp are here numbered (1–3), relative to their pro-distal position within the unexpanded tegular cavity (e.g. see Figs 11F, 17F). Tegular sclerite 1 (TS 1) is a porrect, variably spiniform (Fig. 25F), rod-like (Fig. 20E) or filiform (Fig. 10F) process (breakable in some specimens; Fig. 21F) that originates near the prolateral base of the conductor, adjacent to the embedded base of the proximal embolic sclerite; during pedipalpal expansion this sclerite usually remains distally directed, positioned adjacent to the embolic haematodocha (Figs 26D, 27E). Tegular sclerite 2 (TS 2) is a distinctive, pointed, usually spur-like process, angled obliquely towards the conductor (Figs 11F, 25F), which is closely associated with the adjacent tegular sclerite 2a (TS 2a); in the unexpanded state, the sinuous, filiform TS 2a is usually obscured and ‘locked’ within a folded groove along the margin of TS 2 (see Forster and Platnick 1984, figs 60, 62). Tegular sclerite 3 (TS 3) is the most disto-dorsally positioned of the tegular sclerites, with a broader, more plate-like morphology relative to TS 1–2, usually visible as a distally pointed or rod-like projection beyond the retro-distal rim of the tegulum (Figs 14E-F, 17E-F, 20D-E).

#### Distribution.

Assassin spiders occur in mesic habitats throughout south-eastern, south-western and north-eastern mainland Australia (Fig. 2), usually in montane rainforests (Figs 30C, 38C, 41C) and wet eucalypt forests (Figs 39C, 42C, 45C), but occasionally in temperate heathlands or lowland rainforests (Fig. 40C). In south-eastern Australia they occur on Kangaroo Island (South Australia) and along the Great Dividing Range, from Grampians National Park in south-western Victoria north to Kroombit Tops National Park in south-eastern Queensland. In south-western Western Australia they occur from the Leeuwin-Naturaliste National Park east to Cape Le Grand National Park, with outlying populations in the Porongurup and Stirling Range National Parks. In north-eastern Queensland archaeids occur along the Great Dividing Range, from Eungella National Park near Mackay north to the Mount Finnigan Uplands, near Cooktown. Although this distribution is markedly concordant with the distribution of closed and tall open forests in Australia’s east and extreme south-west (see Specht 1981), assassin spiders appear to be notably absent from Tasmania, from the Australian Alps and from the ‘St Lawrence Gap’ (Webb and Tracey 1981) (Fig. 2), as evidenced by the lack of museum specimens and despite targeted searches by the senior author.

#### Composition.

Five described species – *Austrarchaea daviesae* Forster & Platnick, 1984, *Austrarchaea hickmani* (Butler, 1929), *Austrarchaea mainae* Platnick, 1991b, *Austrarchaea nodosa* (Forster, 1956) and *Austrarchaea robinsi* Harvey, 2002a – and the 17 new species from mid-eastern Australia: *Austrarchaea alani* sp. n., *Austrarchaea aleenae* sp. n., *Austrarchaea binfordae* sp. n., *Austrarchaea christopheri* sp. n., *Austrarchaea clyneae* sp. n., *Austrarchaea cunninghami* sp. n., *Austrarchaea dianneae* sp. n., *Austrarchaea harmsi* sp. n., *Austrarchaea helenae* sp. n., *Austrarchaea judyae* sp. n., *Austrarchaea mascordi* sp. n., *Austrarchaea mcguiganae* sp. n., *Austrarchaea milledgei* sp. n., *Austrarchaea monteithi* sp. n., *Austrarchaea platnickorum* sp. n., *Austrarchaea raveni* sp. n. and *Austrarchaea smithae* sp. n.

#### Remarks.

At least three clades of Archaeidae can be recognised in Australia (Fig. 3B; see also Wood et al. 2010): a mid-eastern Australian clade, distributed from southern New South Wales to south-eastern Queensland (including the enigmatic, basal species *Austrarchaea monteithi* sp. n.); a north-eastern Queensland clade, endemic to tropical Queensland; and a southern Australian clade, known from Victoria, South Australia and south-western Western Australia. For the purposes of this revision, mid-eastern Australian species are diagnosed relative only to other related species from mid-eastern Australia (i.e. *Austrarchaea nodosa* and its closest relatives; Fig. 3B), all of which possess five or six dorsal hump-like tubercles on the abdomen (Figs 5F-G) and have a carapace height to carapace length (CH/CL) ratio ≥ 2.00. *Austrarchaea daviesae* and related species from north-eastern Queensland have only two pairs of hump-like tubercles on the abdomen (Fig. 5E), and *Austrarchaea hickmani*, *Austrarchaea robinsi* and *Austrarchaea mainae* from southern Australia have a carapace height to carapace length (CH/CL) ratio significantly less than 2.00 (M. Rix, pers. obs.).

#### Key to the species of Austrarchaea known from mid-eastern Australia (males required)

**Table:** 

1	Abdomen with five dorsal hump-like tubercles (Fig. 5F)	*Austrarchaea monteithi* sp. n.
–	Abdomen with six dorsal hump-like tubercles, in three pairs (Fig. 5G)	2
2	Male chelicerae with dense tuft of accessory setae on anterior face of paturon (Figs 16C, 17C, 23C)	3
–	Male chelicerae with uniform brush (Figs 12C, 19C, 22C) or comb (Figs 14C, 18C) of accessory setae on anterior face of paturon	5
3	Tuft of accessory setae on anterior face of male paturon very strong, dorsally-directed, with ‘pick-like’ profile in lateral view (Fig. 16C)	*Austrarchaea harmsi* sp. n.
–	Tuft of accessory setae on anterior face of male paturon less pronounced, with shorter, densely-bunched profile in lateral view (Figs 17C, 23C)	4
4	Tegular sclerite 3 (TS 3) very large, porrect (Figs 17D-F); TS 2 thin, spiniform (Fig. 17F)	*Austrarchaea aleenae* sp. n.
–	Tegular sclerite 3 (TS 3) not enlarged, rounded-rectangular (Fig. 23E); TS 2 spur-like, not spiniform (Fig. 23E)	*Austrarchaea milledgei* sp. n.
5	Tegular sclerite 1 (TS 1) very long, rod-like, visible in retrolateral view, reaching to near distal apex of conductor, with broadly-rounded apex (Figs 20C-E)	*Austrarchaea christopheri* sp. n.
–	Tegular sclerite 1 (TS 1) usually relatively short, obscured by conductor in retrolateral view (Figs 18F, 25F); if TS 1 long, never with broadly-rounded apex (Figs 10F, 22F)	6
6	Highest point of male pars cephalica (HPC) near posterior margin of ‘head’ (with carapace sometimes almost horizontal anterior to HPC; Figs 8A, 8H), ratio of HPC to post-ocular length = 0.84 (Figs 8A, 8C-D, 8H, 9D)	7
–	Highest point of male pars cephalica (HPC) closer to middle of ‘head’, ratio of HPC to post-ocular length < 0.75 (Figs 8F-G, 8I, 9C, 9F-I)	11
7	Male chelicerae with short comb of accessory setae on anterior face of paturon (Figs 14C, 18C)	8
–	Male chelicerae with longer brush of accessory setae on anterior face of paturon (Figs 11C, 15C)	9
8	Conductor ‘ear-shaped’, with large proximal lobe (Figs 14D-F); tegular sclerite 3 (TS 3) triangular, with pointed apex (Fig. 14E)	*Austrarchaea raveni* sp. n.
–	Conductor foliate, obliquely-angled (Figs 18D-E); tegular sclerite 3 (TS 3) very large, porrect, with broadly-pointed rectangular apex (Figs 18D-F)	*Austrarchaea alani* sp. n.
9	Tegular sclerite 1 (TS 1) very long, spiniform, visible in retrolateral view, reaching to near distal apex of conductor, with sharply-pointed apex (Figs 22E-F)	*Austrarchaea binfordae* sp. n.
–	Tegular sclerite 1 (TS 1) relatively short, shorter than TS 2, obscured by conductor in retrolateral view (Figs 11F, 15F)	10
10	Conductor ‘spade-shaped’, with sharply-incised proximal margin (Figs 15D-F); male ‘head’ strongly elevated postero-dorsally, post-ocular ratio > 0.40 (Fig. 8C)	*Austrarchaea judyae* sp. n.
–	Conductor foliate, without sharply-incised proximal margin (Figs 11D-E); male ‘head’ not strongly elevated dorsally, post-ocular ratio < 0.30 (Fig. 8H)	*Austrarchaea dianneae* sp. n.
11	Tegular sclerite 1 (TS 1) very thin, filiform (Figs 10F, 24F)	12
–	Tegular sclerite 1 (TS 1) broader, spiniform or rod-like (Figs 12F, 13E, 21F, 25F)	13
12	Proximal portion of embolic sclerite very broad, flanged, overlying proximal conductor (Figs 10D-E).	*Austrarchaea nodosa* (Forster, 1956)
–	Proximal portion of embolic sclerite not flanged, fully-embraced by conductor (Figs 24D-E)	*Austrarchaea mascordi* sp. n.
13	Tegular sclerite 1 (TS 1) rod-like, without sharply-pointed apex (Fig. 27E)	*Austrarchaea mcguiganae* sp. n.
–	Tegular sclerite 1 (TS 1) usually spiniform, with sharply-pointed apex (Figs 12F, 13E, 21F, 25F, 26D)	14
14	Male ‘head’ strongly elevated dorsally, post-ocular ratio > 0.38 (Fig. 9G); highest point of pars cephalica (HPC) approaching posterior quarter of ‘head’, ratio of HPC to post-ocular length ~0.70 (Fig. 9G)	*Austrarchaea smithae* sp. n.
–	Male ‘head’ not strongly elevated dorsally, post-ocular ratio = 0.35 (Figs 8F-G, 9C, 9I); highest point of pars cephalica (HPC) near middle of ‘head’, ratio of HPC to post-ocular length < 0.65 (Figs 8F-G, 9C, 9I)	15
15	Male ‘head’ with concave depression near posterior margin (Fig. 8F)	*Austrarchaea clyneae* sp. n.
–	Male ‘head’ without concave depression near posterior margin (Figs 8G, 9C, 9I)	16
16	Tegular sclerite 1 (TS 1) spiniform, with long, gently-tapered apex (Figs 21F, 26D)	17
–	Tegular sclerite 1 (TS 1) relatively short, with rectangular base and sharply-tapered apex (Fig. 12F)	*Austrarchaea cunninghami* sp. n.
17	Tegular sclerite 1 (TS 1) with curled distal tip (Fig. 26D)	*Austrarchaea helenae* sp. n.
–	Tegular sclerite 1 (TS 1) straight, without curled distal tip (Fig. 21F)	*Austrarchaea platnickorum* sp. n.

### The south-eastern Queensland (including Border Ranges) fauna

#### Austrarchaea nodosa McPherson Range Assassin Spider

(Forster, 1956)

http://species-id.net/wiki/Austrarchaea_nodosa

Figs 1A-B 5D 7I 8I 10 28

Archaea nodosa Forster, 1956: 151, figs 1–7.
Austrarchaea nodosa (Forster): Forster & Platnick, 1984: 21, figs 4–6, 9–10, 19, 27, 34–35, 57, 60–65.

##### Type material.

Holotype juvenile: Lamington National Park, Tullawallal [Antarctic Beech forest], south of Binna Burra, Queensland, Australia, [28°12'20"S, 153°11'20"E], from moss, 31.X.1955, T. Woodward (QMB W1955).

##### Other material examined.

**AUSTRALIA:** Queensland**:** **Lamington National Park:** Binna Burra, track to Tullawallal Antarctic Beech forest, 28°12'20"S, 153°11'20"E, sifting and teasing low vegetation, 7.IV.2006, M. & A. Rix, 1♂, 2 juveniles (WAM T89592DNA: LAM-51-J); Binna Burra, 11.II.1971, Y. Lubin, R. Raven, V. Davies, 1♀ (QMB S73925); Binna Burra, Ships Stern Circuit track, 28°11'51"S, 153°11'28"E, sifting elevated leaf litter, subtropical rainforest, 764 m, 25.IV.2010, M. & A. Rix, D. & S. Harms, J. Wojcieszek, 1 juvenile (WAM T112571DNA: Ar56-58-J); IBISCA Plot IQ-1100-A, 28°15'29"S, 153°09'32"E, bark spray, 1141 m, 11.III.2007, G. Thompson, A. Marcora, 1♂, 1♀, 1 juvenile (QMB S75416). New South Wales**:** **Border Ranges National Park:** Upper Brindle Creek, Wiangarie, 28°23'S, 153°06'E, pyrethrum, *Nothofagus* rainforest, 840 m, 15.XII.2008, G. Monteith, 1♀ (QMB S87983). **Mount Warning National Park:** 1975–1976, G. & S. Monteith, 1 juvenile (QMB S20426); Mount Warning, track to summit, 28°24'08"S, 153°16'27"E, sifting elevated leaf litter under *Xanthorrhoea*, wet eucalypt forest bordering subtropical rainforest, 728 m, 26.IV.2010, M. Rix, 1 juvenile (WAM T112572DNA: Ar57-46-J); off Mount Warning Road, 28°23'51"S, 153°17'20"E, sifting elevated leaf litter, subtropical rainforest, 348 m, 26.IV.2010, D. Harms, 1 juvenile (WAM T112573DNA: Ar58-53-J).

##### Additional material examined (of tentative identification).

**AUSTRALIA:** Queensland**: Lamington National Park:** Mount Hobwee, in moss, 3.IV.1976, R. Raven, 1 juvenile (QMB S30827); Nagarigoon, 8.IV.1976, 1 juvenile (QMB S30817). **Mount Chinghee National Park:** QM Berlesate, stick brushing, 17.XII.1982, G. Monteith, D. Yeates, G. Thompson, 1 juvenile (QMB S30804). New South Wales**:** **Border Ranges National Park:** Border Fence, Levers Plateau, via Rathdowney, pitfall trap, 670 m, 22.V.–IX.1976, G. & S. Monteith, 1 juvenile (QMB S30823).

##### Additional material (not examined).

**AUSTRALIA:** Queensland**: Lamington National Park:** Tullawallal Antarctic Beech forest, south of Binna Burra, 28°12'39"S, 153°11'32"E, *Nothofagus* rainforest, 900 m, 21.III.2006, C. Griswold, D. Silva, R. Raven, B. Baehr, M. Ramírez, 1♂ (CASENT 9018966); Binna Burra, 27.III.1976, R. Raven, V. Davies, 1♀, 1 juvenile (QMB S30820); Binna Burra, 28°11'38"S, 153°11'13"E, rainforest, 790 m, 21-23.III.2006, C. Griswold, D. Silva, R. Raven, B. Baehr, M. Ramírez, 1 juvenile (CASENT 9018963); Binna Burra, 28°11'38"S, 153°11'13"E, Berlese of leaf litter, rainforest, 790 m, 23.III.2006, C. Griswold, D. Silva, R. Raven, B. Baehr, M. Ramírez, 1 juvenile (CASENT 9018964); Binna Burra, along Border Track, 28°11'56"S, 153°11'15"E, beating vegetation, 900 m, 29–30.IV.2009, H. Wood, 1♂ (CASENT 9028426); same data, 1♂ (CASENT 9028388); O'Reillys, 25-26.IX.1986, J. Gallon, R. Raven, 1♀ (QMB S30814).

##### Diagnosis.

**Figure 7.**
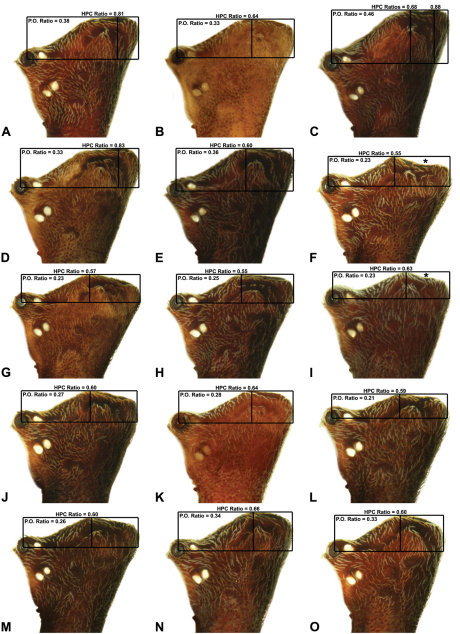
Lateral ‘head’ profiles of females of species of *Austrarchaea* from mid-eastern Australia, showing variation in carapace shape as quantified by the post-ocular ratio (P.O. Ratio) and ratio of highest point of carapace relative to post-ocular length (HPC Ratio): **A**, allotype *A. alani* sp. n.; **B**, allotype *A. aleenae* sp. n.; **C**, allotype *A. judyae* sp. n.; **D**, allotype *A. raveni* sp. n.; **E**, allotype *A. harmsi* sp. n.; **F**, allotype *A. monteithi* sp. n.; **G**, allotype *A. cunninghami* sp. n.; **H**, allotype *A. dianneae* sp. n.; **I**, *A. nodosa* (Forster, 1956); **J**, allotype *A. platnickorum* sp. n.; **K**, allotype *A. binfordae* sp. n.; **L**, *A. milledgei* sp. n. (WAM T112568); **M**, allotype *A. mascordi* sp. n.; **N**, allotype *A. smithae* sp. n.; **O**, allotype *A. mcguiganae* sp. n. Asterisks (*) denote concave depressions.

*Austrarchaea nodosa* can be distinguished from all other Archaeidae from mid-eastern Australia by the broad, flanged proximal portion of the embolic sclerite (Figs 10D-E; see also Forster and Platnick 1984, figs 61, 63) and the unique shape of the conductor (Figs 10D-E), which is thin, gently-tapered and slightly bent along its distal half. The presence of a shallow concave depression near the posterior margin of the ‘head’ (Fig. 7I) can also be used to distinguish females from most other species, including the sympatric *Austrarchaea dianneae* sp. n.

This species can also be distinguished from other genotyped taxa from mid-eastern Australia (see Fig. 3B) by the following seven unique nucleotide substitutions for COI (n = 4): A(42), C(393), C(639), C(939), A(960), A(1038), A(1053).

##### Description.

**Figure 8.**
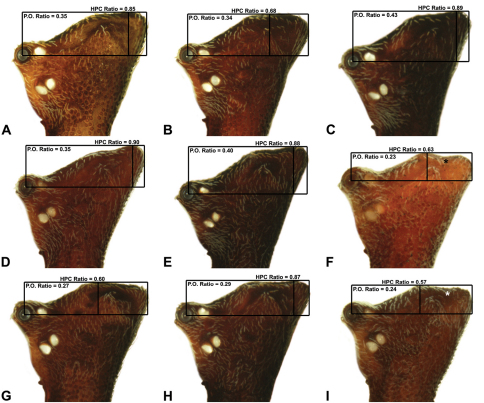
Lateral ‘head’ profiles of males of species of *Austrarchaea* from south-eastern Queensland and extreme north-eastern New South Wales (including the Border Ranges), showing variation in carapace shape as quantified by the post-ocular ratio (P.O. Ratio) and ratio of highest point of carapace relative to post-ocular length (HPC Ratio): **A**, holotype *A. alani* sp. n.; **B**, holotype *A. aleenae* sp. n.; **C**, holotype *A. judyae* sp. n.; **D**, holotype *A. raveni* sp. n.; **E**, holotype *A. harmsi* sp. n.; **F**, holotype *A. clyneae* sp. n.; **G**, holotype *A. cunninghami* sp. n.; **H**, holotype *A. dianneae* sp. n.; **I**, *A. nodosa* (Forster, 1956) (QMB S75416). Asterisks (*) denote concave depressions.

*Male* (QMB S30817): Total length 3.18; leg I femur 3.01; F1/CL ratio 2.70. Cephalothorax dark reddish-brown; legs tan-brown with darker annulations; abdomen mottled grey-brown and beige, with darker reddish-brown dorsal scute and sclerites (Fig. 10B). Carapace very tall (CH/CL ratio 2.30); 1.12 long, 2.56 high, 1.08 wide; ‘neck’ 0.56 wide; bearing two pairs of rudimentary horns; highest point of pars cephalica (HPC) near middle of ‘head’ (ratio of HPC to post-ocular length 0.57), carapace with shallow concave depression posterior to HPC; ‘head’ not strongly elevated dorsally (post-ocular ratio 0.24) (Fig. 8I). Chelicerae with short brush of accessory setae on anterior face of paturon (Fig. 10C). Abdomen 1.64 long, 1.13 wide; with three pairs of dorsal hump-like tubercles (HT 1–6); dorsal scute fused anteriorly to epigastric sclerites, extending posteriorly to first pair of hump-like tubercles; HT 3–6 each covered by separate dorsal sclerites. Unexpanded pedipalp (WAM T89592) (Figs 10D-F) with thin, pointed conductor, gently-tapered and slightly bent along distal half; embolic sclerite with broad, flanged proximal portion overlying proximal conductor; tegular sclerite 1 (TS 1) long, filiform, with sinuous distal tip, visible in retrolateral view; TS 2 spiniform, shorter than TS 1; TS 2a sinuous, largely obscured by TS 2; TS 3 indistinct, embedded within distal haematodocha, barely visible beyond retro-distal rim of tegulum.

*Female* (QMB S30817): Total length 3.54; leg I femur 3.01; F1/CL ratio 2.40. Cephalothorax dark reddish-brown; legs tan-brown with darker annulations; abdomen bi-coloured grey-brown and beige, palest posteriorly (Fig. 10A). Carapace tall (CH/CL ratio 2.12); 1.26 long, 2.67 high, 1.15 wide; ‘neck’ 0.64 wide; bearing two pairs of rudimentary horns; highest point of pars cephalica (HPC) near posterior third of ‘head’ (ratio of HPC to post-ocular length 0.63), carapace with shallow concave depression posterior to HPC; ‘head’ not strongly elevated dorsally (post-ocular ratio 0.23) (Fig. 7I). Chelicerae without accessory setae on anterior face of paturon. Abdomen 2.15 long, 1.64 wide; with three pairs of dorsal hump-like tubercles (HT 1–6). Internal genitalia with cluster of ≤ 12 variably shaped spermathecae on either side of gonopore, clusters meeting near midline of genital plate (Figs 5D, 10G); innermost (anterior) spermathecae longest, sausage-shaped, curved antero-laterally; outermost (posterior) spermathecae bulbous; other spermathecae variably pyriform, straight, directed antero-laterally.

*Variation*: Males (n=2): total length 2.97–3.18; carapace length 1.12–1.13; carapace height 2.56–2.67; CH/CL ratio 2.30–2.36. Females (n=3): total length 3.54–4.00; carapace length 1.21–1.33; carapace height 2.49–2.87; CH/CL ratio 2.06–2.15.

##### Distribution and habitat.

*Austrarchaea nodosa* is known from rainforest habitats along the McPherson Range and ‘scenic rim’ of extreme south-eastern Queensland and north-eastern New South Wales, in the Lamington, Border Ranges and Mount Warning National Parks (Fig. 28). At Binna Burra (Lamington National Park) it has been found in sympatry with *Austrarchaea dianneae* sp. n., in the only known example of two-species sympatry among Australian archaeids (see Nomenclatural Remarks, below).

##### Conservation status.

This species has a relatively widespread distribution in several National Parks protected under World Heritage legislation, and is not considered to be of conservation concern.

##### Nomenclatural remarks.

The holotype specimen of *Austrarchaea nodosa*, described by Forster (1956), is a juvenile (probably penultimate) female from the Tullawallal *Nothofagus* forest near Binna Burra, Lamington National Park. Although long assumed to have only a single species, the greater Binna Burra region is now the only locality in Australia known to have two species of Archaeidae living in close sympatry: numerous specimens of *Austrarchaea dianneae* sp. n. were discovered near Binna Burra in April 2010, along the ‘Ships Stern Circuit Track’, along with one juvenile specimen of *Austrarchaea nodosa*. Both *Austrarchaea dianneae* sp. n. and *Austrarchaea nodosa* are closely related (Fig. 3B) rainforest-dwelling taxa, rendering the identification of Forster’s holotype specimen – and therefore the identification of the generic type species – questionable. To address this issue, and to determine which species was actually described by Forster (1956), two lines of evidence are discussed below.

‘Tullawallal’ – the type locality cited by Forster (1956) – is a well-known, high-altitude *Nothofagus moorei* cool-temperate rainforest, situated off Binna Burra’s ‘Border Track’ at around 900 m elevation. The dominant rainforest surrounding Tullawallal is a closed, complex notophyllous vine forest (with isolated warm-temperate and cool-temperate elements), typical of higher elevations throughout the Lamington National Park and McPherson Range (Fig. 28C). In all of the higher-altitude and/or closed rainforests of the Lamington Plateau and Border Ranges National Park, only identifiable specimens of *Austrarchaea nodosa* (as recognised above) have so far been collected. Furthermore, the two male specimens collected at or near Tullawallal (WAM T89592, CASENT 9018966) are also both *Austrarchaea nodosa* as here recognised. In contrast, the three localities where *Austrarchaea dianneae* sp. n. has been found (i.e. along the ‘Ships Stern Circuit Track’ near Binna Burra, Wojigumal Creek, and in the Tamborine National Park) are significantly lower in altitude than Tullawallal and the surrounding ‘Border Track’ region of Binna Burra (764 m, 570 m and 313 m, respectively), with more open ‘mixed’ rainforests and emergent eucalypts at the Binna Burra and Mount Tamborine localities (Fig. 29C).

Secondly, female specimens of both species possess a distinctive ‘head’ morphology; females of *Austrarchaea nodosa* (as here recognised) are characterised by a shallow concave depression posterior to the highest point of the pars cephalica (HPC) (Fig. 7I), whereas females of *Austrarchaea dianneae* sp. n. have no such depression and a significantly more pronounced posterior margin of the ‘head’ (Fig. 7H). The holotype juvenile specimen of *Austrarchaea nodosa* has a clear concave depression posterior to the HPC, and ‘head’ proportions otherwise very similar to the female illustrated in Figure 7I. In contrast, the only known penultimate female specimen of *Austrarchaea dianneae* sp. n., collected from near Binna Burra (WAM T112556), does not have a concave depression posterior to the HPC, and ‘head’ proportions otherwise similar to the allotype female *Austrarchaea dianneae* sp. n. illustrated in Figure 7H.

Clearly, given the identification of specimens collected from the type locality and similar nearby habitats, and the morphology of the holotype juvenile specimen, we are as confident as possible in newly-diagnosing *Austrarchaea nodosa* as the species described above, given an otherwise highly precarious nomenclatural situation.

#### Austrarchaea dianneae Gold Coast Hinterland Assassin Spider

Rix & Harvey sp. n.

urn:lsid:zoobank.org:act:C0149F76-0A44-4DB4-8DAF-088B17161900

http://species-id.net/wiki/Austrarchaea_dianneae

Figs 7H 8H 11 29

##### Type material.

Holotype male: Tamborine National Park, Joalah section, track to Curtis Falls, Queensland, Australia, 27°55'33"S, 153°11'35"E, sifting elevated leaf litter and hand collecting at night, subtropical rainforest, 313 m, 26.IV.2010, M. Rix, D. Harms (QMB S90185).

Paratypes: Allotype female, same data as holotype (QMB S90186); 2 males and 7 juveniles, same data as holotype (WAM T112557DNA: Ar59-60-M/Ar59-61-J/Ar59-62-J).

##### Other material examined.

**AUSTRALIA:** Queensland**: Lamington National Park:** Binna Burra, Ships Stern Circuit track, 28°11'51"S, 153°11'28"E, sifting elevated leaf litter, subtropical rainforest, 764 m, 25.IV.2010, M. & A. Rix, D. & S. Harms, J. Wojcieszek, 2♂, 4 juveniles (WAM T112556DNA: Ar56-54-M/Ar56-55-J/ Ar56-56-J); Wojigumal Creek, 28°12'29"S, 153°11'56"E, pyrethrum, 570 m, 19.III.2008, A. Nakamura, 1♀ (QMB S87980).

##### Additional material examined (of tentative identification).

**AUSTRALIA:** Queensland**: Lamington National Park:** IBISCA Plot IQ-300-C, 28°09'04"S, 153°08'17"E, pitfall trap, 260 m, 23.I.2007, K. Staunton, 1 juvenile (QMB S90181).

##### Etymology.

The specific epithet is a patronym in honour of the late Dianne Wojcieszek (1962–2003), for her love of the Mount Tamborine Hinterland.

##### Diagnosis.

*Austrarchaea dianneae* can be distinguished from all other Archaeidae from mid-eastern Australia except *Austrarchaea cunninghami* sp. n. by the shape of the conductor (Figs 11D-E), which is broad, foliate and curved laterally, with a triangular apex; and from *Austrarchaea cunninghami* sp. n. by the longer, spiniform tegular sclerite 1 (TS 1) (Fig. 11F) and by the more conical, posteriorly elevated shape of the male ‘head’ (Fig. 8H).

This species can also be distinguished from other genotyped taxa from mid-eastern Australia (see Fig. 3B) by the following three unique nucleotide substitutions for COI (n = 6): T(303), G(798), A(1065).

##### Description.

*Holotype male*: Total length 3.03; leg I femur 3.12; F1/CL ratio 2.83. Cephalothorax dark reddish-brown; legs tan-brown with darker annulations; abdomen mottled grey-brown and beige, with darker reddish-brown dorsal scute and sclerites (Fig. 11B). Carapace very tall (CH/CL ratio 2.37); 1.10 long, 2.62 high, 1.02 wide; ‘neck’ 0.51 wide; bearing two pairs of rudimentary horns; highest point of pars cephalica (HPC) near posterior margin of ‘head’ (ratio of HPC to post-ocular length 0.87), carapace gently sloping and almost horizontal anterior to HPC; ‘head’ not strongly elevated dorsally (post-ocular ratio 0.29) (Fig. 8H). Chelicerae with brush of accessory setae on anterior face of paturon (Fig. 11C). Abdomen 1.69 long, 1.33 wide; with three pairs of dorsal hump-like tubercles (HT 1–6); dorsal scute fused anteriorly to epigastric sclerites, extending posteriorly to first pair of hump-like tubercles; HT 3–6 each covered by separate dorsal sclerites. Unexpanded pedipalp (Figs 11D-F) with broad, foliate conductor, curved laterally with triangular apex; tegular sclerite 1 (TS 1) spiniform, obscured by conductor in retrolateral view; TS 2 spur-like, longer than TS 1; TS 2a sinuous, largely obscured by TS 2; TS 3 embedded proximally within distal haematodocha, with sharply-pointed, triangular apex projecting beyond retro-distal rim of tegulum.

*Allotype female*: Total length 3.74; leg I femur 3.18; F1/CL ratio 2.43. Cephalothorax dark reddish-brown; legs tan-brown with darker annulations; abdomen mottled grey-brown and beige, palest posteriorly (Fig. 11A). Carapace very tall (CH/CL ratio 2.28); 1.31 long, 2.97 high, 1.21 wide; ‘neck’ 0.65 wide; bearing two pairs of rudimentary horns; highest point of pars cephalica (HPC) near middle of ‘head’ (ratio of HPC to post-ocular length 0.55), carapace gently sloping posterior to HPC; ‘head’ not strongly elevated dorsally (post-ocular ratio 0.25) (Fig. 7H). Chelicerae without accessory setae on anterior face of paturon. Abdomen 2.15 long, 1.64 wide; with three pairs of dorsal hump-like tubercles (HT 1–6). Internal genitalia with cluster of ≤ 12 variably shaped spermathecae on either side of gonopore, clusters meeting near midline of genital plate (Fig. 11G); innermost (anterior) spermathecae longest, sausage-shaped, curved antero-laterally; other spermathecae variably pyriform, curved, directed laterally.

*Variation*: Males (n=5): total length 2.73–3.21; carapace length 1.09–1.13; carapace height 2.53–2.62; CH/CL ratio 2.24–2.39. Females (n=2): total length 3.64–3.74; carapace length 1.31 (invariable); carapace height 2.87–2.97; CH/CL ratio 2.20–2.28.

##### Distribution and habitat.

*Austrarchaea dianneae* is known only from subtropical rainforest habitats in the Tamborine and Lamington National Parks south of Brisbane, south-eastern Queensland (Fig. 29). At Binna Burra (Lamington National Park) it has been found in sympatry with *Austrarchaea nodosa*, in the only known example of two-species sympatry among Australian archaeids (see Nomenclatural Remarks for *Austrarchaea nodosa*, above).

##### Conservation status.

This species is a short-range endemic taxon (Harvey 2002b), which although restricted in distribution, is abundant within the Tamborine National Park (M. Rix, pers. obs.) and is further protected within the World Heritage-listed Lamington National Park. It is not considered to be of conservation concern.

#### Austrarchaea cunninghami Main Range Assassin Spider

Rix & Harvey sp. n.

urn:lsid:zoobank.org:act:EFE94CB8-B85A-4573-B181-E6279995D9B2

http://species-id.net/wiki/Austrarchaea_cunninghami

Figs 7G 8G 12 30

##### Type material.

Holotype male: Main Range National Park, Cunningham's Gap, track to Mount Mitchell, Queensland, Australia, 28°03'05"S, 152°23'41"E, sifting elevated leaf litter, subtropical rainforest and adjacent transitional eucalypt forest, 805 m, 23.IV.2010, M. Rix, D. Harms (QMB S90184).

Paratypes: Allotype female, same data as holotype (QMB S90183); 1 female and 14 juveniles, same data as holotype (WAM T112555DNA: Ar55-89-F/Ar55-90-J/Ar55-91-J).

##### Other material examined.

**AUSTRALIA:** Queensland**:** **Main Range National Park:** Mount Mitchell, pitfall, 1060 m, 1.III.1992, D. Cook, 1 juvenile (QMB S25714).

##### Additional material examined (of tentative identification).

**AUSTRALIA:** Queensland**: Main Range National Park:** Mount Superbus, summit, pyrethrum, trees and logs, 1300 m, 8-9.II.1990, G. Monteith, G. Thompson, H. Janetski, 2 juveniles (QMB S38509); Mount Asplenium, pyrethrum, trees and logs, 1290 m, 30.I.1993, G. Monteith, 1 juvenile (QMB S90179).

##### Etymology.

The specific epithet is a patronym in honour of British botanist and explorer Allan Cunningham (1791–1839), after whom the type locality of this species – Cunningham’s Gap in the Main Range National Park – is named.

##### Diagnosis.

*Austrarchaea cunninghami* can be distinguished from all other Archaeidae from mid-eastern Australia except *Austrarchaea dianneae* by the shape of the conductor (Figs 12D-E), which is broad, foliate and curved laterally, with a triangular apex; and from *Austrarchaea dianneae* by the shorter, sharply-tapered tegular sclerite 1 (TS 1) (Fig. 12F) and by the more rounded, less conical shape of the male ‘head’ (Fig. 8G).

This species can also be distinguished from other genotyped taxa from mid-eastern Australia (see Fig. 3B) by the following four unique nucleotide substitutions for COI and COII (n = 3): C(769), C(981), C(1140), G(1152).

##### Description.

*Holotype male*: Total length 2.82; leg I femur 3.01; F1/CL ratio 2.70. Cephalothorax dark reddish-brown; legs tan-brown with darker annulations; abdomen mottled grey-brown and beige, with darker brown dorsal scute and sclerites (Fig. 12B). Carapace very tall (CH/CL ratio 2.21); 1.12 long, 2.46 high, 1.05 wide; ‘neck’ 0.56 wide; bearing two pairs of rudimentary horns; highest point of pars cephalica (HPC) near middle of ‘head’ (ratio of HPC to post-ocular length 0.60), carapace gently sloping posterior to HPC; ‘head’ not strongly elevated dorsally (post-ocular ratio 0.27) (Fig. 8G). Chelicerae with brush of accessory setae on anterior face of paturon (Fig. 12C). Abdomen 1.46 long, 0.97 wide; with three pairs of dorsal hump-like tubercles (HT 1–6); dorsal scute fused anteriorly to epigastric sclerites, extending posteriorly to first pair of hump-like tubercles; HT 3–6 each covered by separate dorsal sclerites. Unexpanded pedipalp (Figs 12D-F) with broad, foliate conductor, strongly curved laterally with triangular, evenly-tapered apex; tegular sclerite 1 (TS 1) relatively short, with rectangular base and sharply-tapered apex, obscured by conductor in retrolateral view; TS 2 spur-like, longer than TS 1; TS 2a sinuous, filiform, exposed distally; TS 3 embedded proximally within distal haematodocha, with sharply-pointed apex projecting beyond retro-distal rim of tegulum.

*Allotype female*: Total length 3.54; leg I femur 3.24; F1/CL ratio 2.30. Cephalothorax brown; legs tan-brown with darker annulations; abdomen mottled grey-brown and beige (Fig. 12A). Carapace tall (CH/CL ratio 2.20); 1.41 long, 3.10 high, 1.28 wide; ‘neck’ 0.76 wide; bearing two pairs of rudimentary horns; highest point of pars cephalica (HPC) near middle of ‘head’ (ratio of HPC to post-ocular length 0.57), carapace gently sloping posterior to HPC; ‘head’ not strongly elevated dorsally (post-ocular ratio 0.23) (Fig. 7G). Chelicerae without accessory setae on anterior face of paturon. Abdomen 1.90 long, 1.41 wide; with three pairs of dorsal hump-like tubercles (HT 1–6). Internal genitalia with cluster of ≤ 10 variably shaped spermathecae on either side of gonopore, clusters marginally separated near midline of genital plate (Fig. 12G); innermost (anterior) spermathecae longest, sausage-shaped, bent laterally; other spermathecae variably pyriform, curved, directed laterally.

*Variation*: Females (n=2): total length 3.44–3.54; carapace length 1.38–1.41; carapace height 2.97–3.10; CH/CL ratio 2.15–2.20.

##### Distribution and habitat.

*Austrarchaea cunninghami* is known only from rainforest habitats in the Main Range National Park of extreme south-eastern Queensland (Fig. 30).

##### Conservation status.

This species is a short-range endemic taxon (Harvey 2002b), which although restricted in distribution, is abundant within the World Heritage-listed Main Range National Park near Cunningham’s Gap (M. Rix, pers. obs.). It is not considered to be of conservation concern.

#### Austrarchaea clyneae Mount Clunie Assassin Spider

Rix & Harvey sp. n.

urn:lsid:zoobank.org:act:3F559C8C-005F-442A-84D1-0676D6EB56A6

http://species-id.net/wiki/Austrarchaea_clyneae

Figs 8F 13 31

##### Type material.

Holotype male: Mount Clunie National Park, Mount Clunie, via Woodenbong, Queensland, Australia, 1975–1976, G. & S. Monteith (QMB S20425).

##### Other material examined.

**AUSTRALIA:** New South Wales**:** **Mount Clunie National Park:** Mount Clunie, via Woodenbong, pitfall trap, 670 m, 8.V.–15.VIII.1976, G. & S. Monteith, 2 juveniles (QMB S69811).

##### Additional material examined (of tentative identification).

**AUSTRALIA:** New South Wales**: Tooloom National Park:** “Beaury State Forest", north along Wallaby Creek, 28°26'S, 152°27'E, 830 m, 9.IV.1993, M. Gray, G. Cassis, 1 juvenile (AMS KS37854).

##### Etymology.

The specific epithet is a patronym in honour of Australian naturalist, zoologist, conservationist, author, wildlife photographer and documentary film-maker Densey Clyne, for her landmark contributions to Australian natural history, and for having such a profound impact on the senior author during his formative childhood years.

##### Diagnosis.

*Austrarchaea clyneae* can be distinguished from all other Archaeidae from mid-eastern Australia by the very long, spiniform tegular sclerite 1 (TS 1) (Fig. 13E) combined with the unique shape of the conductor (Figs 13C-D), which is thin, gently-curved laterally and pointed distally.

##### Description.

*Holotype male*: Total length 2.87; leg I femur 2.72; F1/CL ratio 2.62. Cephalothorax reddish-brown; legs tan-brown with darker annulations; abdomen mottled grey-brown and dark beige, with darker reddish-brown dorsal scute and sclerites (Fig. 13A). Carapace very tall (CH/CL ratio 2.22); 1.04 long, 2.31 high, 0.99 wide; ‘neck’ 0.51 wide; bearing two pairs of rudimentary horns; highest point of pars cephalica (HPC) near posterior third of ‘head’ (ratio of HPC to post-ocular length 0.63), carapace with concave depression posterior to HPC; ‘head’ not strongly elevated dorsally (post-ocular ratio 0.23) (Fig. 8F). Chelicerae with short brush of accessory setae on anterior face of paturon (Fig. 13B). Abdomen 1.54 long, 1.18 wide; with three pairs of dorsal hump-like tubercles (HT 1–6); dorsal scute fused anteriorly to epigastric sclerites, extending posteriorly to first pair of hump-like tubercles; HT 3–6 each covered by separate dorsal sclerites. Unexpanded pedipalp (Figs 13C-E) with thin, gently-curved, pointed conductor; tegular sclerite 1 (TS 1) very long, spiniform, reaching to near distal tip of conductor, visible in retrolateral view; TS 2 spur-like, shorter than TS 1; TS 2a sinuous, largely obscured by TS 2; TS 3 indistinct, embedded within distal haematodocha, barely visible beyond retro-distal rim of tegulum.

*Female*: Unknown.

##### Distribution and habitat.

*Austrarchaea clyneae* is known only from rainforest habitats in the Mount Clunie National Park of extreme north-eastern New South Wales (Fig. 31). A juvenile specimen from Tooloom National Park (near Urbenville) may also belong to this species based on proximity.

##### Conservation status.

This species appears to be a short-range endemic taxon (Harvey 2002b), which although potentially restricted in distribution, seems well-protected in at least one World Heritage-listed National Park. It is not considered to be of conservation concern.

#### Austrarchaea raveni D’Aguilar Range Assassin Spider

Rix & Harvey sp. n.

urn:lsid:zoobank.org:act:8EFF87A1-A86D-452C-8F69-0A4C5191CACA

http://species-id.net/wiki/Austrarchaea_raveni

Figs 1E-F 7D 8D 14 32

##### Type material.

Holotype male: Mount Nebo, D'Aguilar Range, Queensland, Australia, 15.VIII.1990, [inside hollow log], M. Harvey, T. Churchill (QMB S90193).

Paratype: Allotype female, D'Aguilar National Park, Mount Glorious, Maiala section, track to Greene's Falls, Queensland, Australia, 27°19'57"S, 152°45'47"E, sifting elevated leaf litter, subtropical rainforest, 633 m, 4.V.2010, M. Rix, D. Harms (QMB S90192DNA: Ar73-83-F).

##### Other material examined.

**AUSTRALIA:** Queensland**: D'Aguilar National Park:** Mount Glorious, Maiala section, track to Greene's Falls, 27°19'30"S, 152°45'45"E, hand collected at night from maternal web at base of fallen log, 3.III.2001, M. & A. Rix, 1♀, 1 juvenile (WAM T94092); Mount Glorious, Maiala section, track to Greene's Falls, 27°19'57"S, 152°45'47"E, sifting elevated leaf litter, subtropical rainforest, 633 m, 4.V.2010, M. Rix, D. Harms, 6 juveniles (WAM T112574DNA: Ar73-84-J/Ar73-85-J); Mount Glorious, Maiala section, ANIC Berlesate, ~635 m, 13.III.1973, R. Taylor, 1 juvenile (ANIC). **Mount Glorious:** “Mount Glorious", spraying logs with Mortein, 26.IV.1988, P. Blus, V. Davies, 1 juvenile (QMB S30810); Hiller Family's Property, 20.I.– 26.VI.1978, G. Monteith, 1 juvenile (QMB S30807). **Mount Mee Forest Reserve:** The Mill Rainforest Walk, 27°04'57"S, 152°42'39"E, sifting elevated leaf litter, subtropical rainforest, 271 m, 1.V.2010, M. Rix, D. Harms, 1♂, 3 juveniles (WAM T112575DNA: Ar69-86-M/Ar69-87-J/Ar69-88-J).

##### Additional material (not examined).

**AUSTRALIA:** Queensland**: Brisbane Forest Park:** Mount Glorious, Lawton Road section of Westside Track, NW. of Maiala, 27°19'07"S, 152°44'50"E, beating ferns ~20 cm from ground, rainforest, 1.I.2010, G. Anderson, 1 juvenile (QMB).

##### Etymology.

The specific epithet is a patronym in honour of Dr Robert Raven, for his extraordinary contributions to arachnology, and for his ongoing efforts documenting the diverse spider fauna of south-eastern Queensland.

##### Diagnosis.

*Austrarchaea raveni* can be distinguished from all other Archaeidae from mid-eastern Australia by the very short, barely differentiated comb of accessory setae on the male chelicerae (Fig. 14C) combined with the unique shape of the conductor (Figs 14D-E), which is ‘ear-shaped’ with a large proximal lobe.

This species can also be distinguished from other genotyped taxa from mid-eastern Australia (see Fig. 3B) by the following three unique nucleotide substitutions for COI and COII (n = 6): G(9), G(843), T(1408).

##### Description.

*Holotype male*: Total length 2.90; leg I femur 3.10; F1/CL ratio 2.88. Cephalothorax dark reddish-brown; legs tan-brown with darker annulations; abdomen mottled grey-brown and beige, with darker reddish-brown dorsal scute and sclerites (Fig. 14B). Carapace very tall (CH/CL ratio 2.41); 1.08 long, 2.59 high, 0.98 wide; ‘neck’ 0.51 wide; bearing two pairs of rudimentary horns; highest point of pars cephalica (HPC) near posterior margin of ‘head’ (ratio of HPC to post-ocular length 0.90), carapace slightly concave anterior to HPC; ‘head’ moderately elevated postero-dorsally (post-ocular ratio 0.35) (Fig. 8D). Chelicerae with short, barely differentiated comb of accessory setae on anterior face of paturon (Fig. 14C). Abdomen 1.59 long, 1.18 wide; with three pairs of dorsal hump-like tubercles (HT 1–6); dorsal scute fused anteriorly to epigastric sclerites, extending posteriorly to first pair of hump-like tubercles; HT 3–6 each covered by separate dorsal sclerites. Partially expanded pedipalp (Figs 14D-F) with lobed, ‘ear-shaped’ conductor; tegular sclerite 1 (TS 1) spiniform, obscured by conductor in retrolateral view; TS 2 spiniform, longer than TS 1, directed across proximal lobe of conductor; TS 2a sinuous, largely obscured by TS 2; TS 3 embedded proximally within distal haematodocha, with sharply-pointed, broadly triangular apex directed retro-ventrally across conductor.

*Allotype female*: Total length 3.05; leg I femur 3.14; F1/CL ratio 2.58. Cephalothorax brown; legs pale tan-brown with darker annulations; abdomen mottled grey-brown and beige, palest posteriorly (Fig. 14A). Carapace very tall (CH/CL ratio 2.34); 1.22 long, 2.85 high, 1.10 wide; ‘neck’ 0.60 wide; bearing two pairs of rudimentary horns; highest point of pars cephalica (HPC) near posterior margin of ‘head’ (ratio of HPC to post-ocular length 0.83), carapace gently sloping anterior to HPC; ‘head’ moderately elevated postero-dorsally (post-ocular ratio 0.33) (Fig. 7D). Chelicerae without accessory setae on anterior face of paturon. Abdomen 1.59 long, 1.03 wide; with three pairs of dorsal hump-like tubercles (HT 1–6). Internal genitalia with dense cluster of ≤ 15 variably shaped spermathecae on either side of gonopore, clusters meeting near midline of genital plate (Fig. 14G); innermost (anterior) spermathecae sausage-shaped, curved antero-laterally; other spermathecae variably aciniform, straight, directed antero-laterally.

*Variation*: Males (n=2): total length 2.64–2.90; carapace length 1.06–1.08; carapace height 2.46–2.59; CH/CL ratio 2.31–2.41. Females (n=2): total length 3.05–3.46; carapace length 1.22–1.30; carapace height 2.85–3.10; CH/CL ratio 2.34–2.38.

##### Distribution and habitat.

*Austrarchaea raveni* is known only from rainforest habitats at Mount Glorious, Mount Nebo and Mount Mee, on the D’Aguilar Range north-west of Brisbane, south-eastern Queensland (Fig. 32).

##### Conservation status.

This species is a short-range endemic taxon (Harvey 2002b), which although restricted in distribution, is relatively abundant within several National Parks and Forest Reserves (M. Rix, pers. obs.). It is not considered to be of conservation concern.

#### Austrarchaea judyae Sunshine Hinterland Assassin Spider

Rix & Harvey sp. n.

urn:lsid:zoobank.org:act:190BA8C6-1373-45DB-AA36-813FC8DBCA59

http://species-id.net/wiki/Austrarchaea_judyae

Figs 4F-G 5A-C 7C 8C 15 33

##### Type material.

Holotype male: Conondale National Park, walking trail from Booloumba Creek Day Use Area No. 2, Queensland, Australia, 26°38'38"S, 152°38'50"E, sifting elevated leaf litter, subtropical rainforest, 187 m, 30.IV.2010, M. Rix, D. Harms (QMB S90190).

Paratypes: Allotype female, same data as holotype (QMB S90191); 2 females and 2 juveniles, same data as holotype (WAM T112563DNA: Ar67-76-F/Ar67-78-J).

##### Other material examined.

**AUSTRALIA:** Queensland**: Conondale National Park:** off Booloumba Creek Forest Drive, 26°40'45"S, 152°38'06"E, sifting elevated leaf litter, subtropical rainforest, 351 m, 30.IV.2010, M. Rix, D. Harms, 1 juvenile (WAM T112562DNA: Ar66-79-J); Booloumba Creek, leaf litter, 13-18.IV.1976, R. Raven, 1 juvenile (QMB S30822); “Conondale Range", rainforest, 1-3.V.1976, R. Raven, 1 juvenile (QMB S29324); “Conondale National Park", 26°43'30"S, 152°36'00"E, canopy fogging, 26.I.1998, R. Kitching, 1 juvenile (ANIC). **Maleny:** 7 km SE. of Maleny, rainforest, 900 m, 18.VI.–15.VIII.1982, S. & J. Peck, 2♂ (ANIC). **Mapleton Forest Reserve:** Bonyee Walk, off Mapleton Forest Drive, 26°33'28"S, 152°51'58"E, sifting elevated leaf litter, subtropical rainforest, 175 m, 1.V.2010, M. Rix, D. Harms, 1♂, 8 juveniles (WAM T112564DNA: Ar68-80-M/Ar68-81-J/Ar68-82-J).

##### Additional material examined (of tentative identification).

**AUSTRALIA:** Queensland**: Oakview State Forest:** “summit”, 26°10’S, 152°20’E, pyrethrum on trees, rainforest, 600 m, 26.V.2002, G. Monteith, 1 juvenile (QMB S90180).

##### Etymology.

The specific epithet is a patronym in honour of Judy Rix, for her love of the Sunshine Coast hinterland, and for a lifetime of generosity and support to the senior author.

##### Diagnosis.

*Austrarchaea judyae* can be distinguished from all other Archaeidae from mid-eastern Australia by the small body size of males and females (Fig. 6) and by the unique shape of the conductor (Figs 15D-E), which is ‘spade-shaped’ and laterally incised.

This species cannot be distinguished from other genotyped taxa from mid-eastern Australia on the basis of unique nucleotide substitutions, but can be distinguished from all other genotyped taxa from south-eastern Queensland (see Fig. 3B) by the following three nucleotide substitutions for COI and COII (n = 6): G(1010), A(1413), T(1560).

##### Description.

*Holotype male*: Total length 2.44; leg I femur 2.69; F1/CL ratio 2.92. Cephalothorax dark reddish-brown; legs tan-brown with darker annulations; abdomen mottled grey-brown and beige, palest posteriorly, with darker reddish-brown dorsal scute and sclerites (Fig. 15B). Carapace very tall (CH/CL ratio 2.38); 0.92 long, 2.19 high, 0.85 wide; ‘neck’ 0.42 wide; bearing two pairs of rudimentary horns; highest point of pars cephalica (HPC) near posterior margin of ‘head’ (ratio of HPC to post-ocular length 0.89), carapace slightly concave anterior to HPC; ‘head’ strongly elevated postero-dorsally (post-ocular ratio 0.43) (Fig. 8C). Chelicerae with short brush of accessory setae on anterior face of paturon (Figs 4F, 15C). Abdomen 1.28 long, 0.97 wide; with three pairs of dorsal hump-like tubercles (HT 1–6); dorsal scute fused anteriorly to epigastric sclerites, extending posteriorly to first pair of hump-like tubercles; HT 3–6 each covered by separate dorsal sclerites (Fig. 5A). Unexpanded pedipalp (Figs 15D-F) with laterally incised, ‘spade-shaped’ conductor; tegular sclerite 1 (TS 1) spiniform, obscured by conductor in retrolateral view; TS 2 spiniform, longer than TS 1; TS 2a sinuous, largely obscured by TS 2; TS 3 embedded proximally within distal haematodocha, with broadly-pointed apex projecting beyond retro-distal rim of tegulum.

*Allotype female*: Total length 3.08; leg I femur 2.97; F1/CL ratio 2.70. Cephalothorax dark reddish-brown; legs tan-brown with darker annulations; abdomen mottled grey-brown and beige, palest behind hump-like tubercles (Fig. 15A). Carapace very tall (CH/CL ratio 2.41); 1.10 long, 2.65 high, 0.97 wide; ‘neck’ 0.54 wide; bearing two pairs of rudimentary horns; dual highest points of pars cephalica (HPC1–2) near posterior third of ‘head’(ratio of HPC1 to post-ocular length 0.68) and near posterior margin of ‘head’ (ratio of HPC2 to post-ocular length 0.88), carapace slightly concave between HPC1 and HPC2; ‘head’ strongly elevated postero-dorsally (post-ocular ratio 0.46) (Fig. 7C). Chelicerae without accessory setae on anterior face of paturon. Abdomen 1.90 long, 1.54 wide; with three pairs of dorsal hump-like tubercles (HT 1–6). Internal genitalia with dense cluster of ≤ 15 variably shaped spermathecae on either side of gonopore, clusters meeting near midline of genital plate (Fig. 15G); innermost (anterior) spermathecae longest, sausage-shaped, curved antero-laterally; outermost (posterior) spermathecae bulbous; other spermathecae variably pyriform, straight, directed antero-laterally.

*Variation*: Males (n=4): total length 2.44–2.51; carapace length 0.92–0.95; carapace height 2.19–2.31; CH/CL ratio 2.37–2.43. Females (n=3): total length 2.67–3.08; carapace length 1.03–1.10; carapace height 2.46–2.65; CH/CL ratio 2.30–2.41. Two male specimens from near Maleny (ANIC) are in poor condition, but seem to have a slightly broader, less markedly incised conductor, suggesting that there may be some population-level variation in the shape of the conductor in this species.

##### Distribution and habitat.

*Austrarchaea judyae* is known from rainforest habitats on the Blackall and Conondale Ranges of south-eastern Queensland, in the Conondale National Park, Mapleton Forest Reserve and in the region surrounding Maleny/Montville (Fig. 33). A juvenile specimen from Oakview State Forest (near Gympie) may also belong to this species based on proximity.

##### Conservation status.

This species has a relatively widespread distribution in several National Parks and Forest Reserves, and is not considered to be of conservation concern.

#### Austrarchaea harmsi Bunya Mountains Assassin Spider

Rix & Harvey sp. n.

urn:lsid:zoobank.org:act:E8650140-16BF-40B4-AC61-F787040DD692

http://species-id.net/wiki/Austrarchaea_harmsi

Figs 7E 8E 16 34

##### Type material.

Holotype male, Bunya Mountains National Park, Dandabah, Scenic Circuit track, ~400 m from entrance, Queensland, Australia, 26°52'43"S, 151°35'53"E, sifting elevated leaf litter, subtropical araucarian rainforest, 959 m, 2.V.2010, M. Rix, D. Harms (QMB S90189).

Paratypes: Allotype female, Bunya Mountains National Park, Dandabah, off Bunya Mountains Road, Queensland, Australia, 26°53'07"S, 151°35'38"E, sifting elevated leaf litter, subtropical araucarian rainforest, 1030 m, 2.V.2010, M. Rix, D. Harms (QMB S90187); 1 male, same data as holotype (QMB S90188); 2 males and 3 juveniles, same data as holotype (WAM T112559DNA: Ar70-73-M/Ar70-74-J/Ar70-75-J).

##### Other material examined.

**AUSTRALIA:** Queensland**: Bunya Mountains National Park:** Dandabah, on tree trunk at night, 3.III.1976, 1♂ (QMB S1095); off Bunya Mountains Road, 26°53'00"S, 151°35'20"E, sifting elevated leaf litter, subtropical araucarian rainforest, 917 m, 2.V.2010, M. Rix, D. Harms, 3 juveniles (WAM T112560DNA: Ar71-71-J/Ar71-72-J); adjacent to Stirling Family's Property, ~1.5 km SE. of Dandabah, beating low-hanging Bunya Pine branch in rainforest, 7-10.XI.2005, M. Rix, 1 juvenile (WAM T94093); Marlaybrook, 1.III.1976, V. Davies, R. Raven, 1 juvenile (QMB S30826).

##### Additional material (not examined).

**AUSTRALIA:** Queensland**: Bunya Mountains National Park:** track starting from Paradise carpark going towards Westcliff lookout, 26°52'33"S, 151°34'24"E, shaking dense mats of grass, transition zone between araucarian rainforest and grasslands, 1040 m, 3.V.2009, H. Wood, 1♂ (CASENT 9028427); same data, 1♂ (CASENT 9034524); same data, 2♀ (CASENT 9028386).

##### Etymology.

The specific epithet is a patronym in honour of Danilo Harms, for his contributions to arachnology, and his invaluable assistance to the senior author during field work in south-eastern Australia.

##### Diagnosis.

*Austrarchaea harmsi* can be distinguished from all other Archaeidae from mid-eastern Australia by the dense, pick-like tuft of accessory setae on the male chelicerae (Fig. 16C) and by the unique shape of the conductor (Figs 16D-E), which is ‘shield-shaped’ and twisted proximally.

This species can also be distinguished from other genotyped taxa from mid-eastern Australia (see Fig. 3B) by the following eight unique nucleotide substitutions for COI and COII (n = 5): C(57), A(756), A(798), C(1061), C(1191), A(1294), T(1465), A(1467).

##### Description.

*Holotype male*: Total length 2.67; leg I femur 2.67; F1/CL ratio 2.57. Cephalothorax dark reddish-brown; legs tan-brown with darker annulations; abdomen mottled grey-brown brown and beige, with darker reddish-brown dorsal scute and sclerites (Fig. 16B). Carapace tall (CH/CL ratio 2.12); 1.04 long, 2.21 high, 0.97 wide; ‘neck’ 0.46 wide; bearing two pairs of rudimentary horns; highest point of pars cephalica (HPC) near posterior margin of ‘head’ (ratio of HPC to post-ocular length 0.88), carapace slightly concave anterior to HPC; ‘head’ strongly elevated postero-dorsally (post-ocular ratio 0.40) (Fig. 8E). Chelicerae with dense, pick-like tuft of accessory setae on anterior face of paturon (Fig. 16C). Abdomen 1.44 long, 1.05 wide; with three pairs of dorsal hump-like tubercles (HT 1–6); dorsal scute fused anteriorly to epigastric sclerites, extending posteriorly to first pair of hump-like tubercles; HT 3–6 each covered by separate dorsal sclerites. Unexpanded pedipalp (Figs 16D-F) with twisted, ‘shield-shaped’ conductor; tegular sclerite 1 (TS 1) relatively short, spiniform, obscured by conductor in retrolateral view; TS 2 spur-like, sinuous, longer than TS 1; TS 2a sinuous, largely obscured by TS 2; TS 3 porrect, spur-like, with sharply-pointed apex mostly obscured in retrolateral view by haematodochal membranes and retro-distal rim of tegulum.

*Allotype female*: Total length 3.28; leg I femur 2.72; F1/CL ratio 2.28. Cephalothorax dark reddish-brown; legs tan-brown with darker annulations; abdomen mottled grey-brown and beige (Fig. 16A). Carapace tall (CH/CL ratio 2.09); 1.19 long, 2.49 high, 1.08 wide; ‘neck’ 0.56 wide; bearing two pairs of rudimentary horns (lateral pair asymmetrically reduced); highest point of pars cephalica (HPC) near middle of ‘head’ (ratio of HPC to post-ocular length 0.60), carapace gently sloping posterior to HPC; ‘head’ moderately elevated postero-dorsally (post-ocular ratio 0.36) (Fig. 7E). Chelicerae without accessory setae on anterior face of paturon. Abdomen 1.90 long, 1.44 wide; with three pairs of dorsal hump-like tubercles (HT 1–6). Internal genitalia with dense cluster of ≤ 15 variably shaped spermathecae on either side of gonopore, clusters meeting near midline of genital plate (Fig. 16G); innermost (anterior) spermathecae longest, sausage-shaped, curved antero-laterally; other spermathecae variably pyriform, straight, directed antero-laterally.

*Variation*: Males (n=5): total length 2.64–3.05; carapace length 1.04–1.08; carapace height 2.15–2.24; CH/CL ratio 2.08–2.14.

##### Distribution and habitat.

*Austrarchaea harmsi* is known only from araucarian rainforest habitats in the Bunya Mountains National Park of south-eastern Queensland (Fig. 34).

##### Conservation status.

This species is a short-range endemic taxon (Harvey 2002b), which although restricted in distribution, is abundant within the Bunya Mountains National Park (M. Rix, pers. obs.). It is not considered to be of conservation concern.

#### Austrarchaea aleenae Bulburin Assassin Spider

Rix & Harvey sp. n.

urn:lsid:zoobank.org:act:B80C6FF2-DD73-44D8-BD1C-5153A64E125F

http://species-id.net/wiki/Austrarchaea_aleenae

Figs 5G 7B 8B 17 35

##### Type material.

Holotype male: Bulburin National Park, via Builyan, off Bulburin Forest Road, Queensland, Australia, 24°31'17"S, 151°28'02"E, sifting elevated leaf litter, subtropical vine rainforest, 618 m, 25.X.2010, M. & A. Rix (QMB S90182).

Paratypes: Allotype female, Bulburin National Park (written “Bulburin State Forest"), Queensland, Australia, 25.II.–8.III.1977, R. Raven, V. Davies (QMB S1094); 1 male and 4 juveniles, same data as holotype (WAM T112552DNA: BUL-68-M/BUL-69-J/BUL-70-J).

##### Other material examined.

**AUSTRALIA:** Queensland**: Bulburin National Park:** “Bulburin State Forest", 19.III.1975, 1♂, 2 juveniles (QMB S1099); “Bulburin Forestry Nursery", NW. of Bundaberg, under rock in log, rainforest, 580 m, III.1975, M. Gray, C. Horseman, 2♀, 4 juveniles (AMS KS6776); same data, 2 juveniles (AMS KS87). **Kalpowar State Forest:** Mount Fort William, via Kalpowar, pyrethrum, logs, 18.I.1990, G. Monteith, 1♀, 2 juveniles (QMB S25803); Mount Fort William, 6 km NE. of Kalpowar, pyrethrum in rainforest, 700 m, 18.IX.1989, G. Monteith, 1 juvenile (QMB S31311).

##### Etymology.

The specific epithet is a patronym in honour of Aleena Wojcieszek, for her love of assassin spiders, and for her support of the senior author over many years.

##### Diagnosis.

*Austrarchaea aleenae* can be distinguished from all other Archaeidae from mid-eastern Australia except *Austrarchaea alani* sp. n. by the very large, porrect tegular sclerite 3 (TS 3) (Figs 17D-F); and from *Austrarchaea alani* sp. n. by the dense tuft of accessory setae on the male chelicerae (Fig. 17C).

This species can also be distinguished from other genotyped taxa from mid-eastern Australia (see Fig. 3B) by the following unique nucleotide substitution for COI (n = 3): A(429). The COI and COII substitutions G(363), A(552), G(627), T(897), G(1020), G(1029), G(1317) and T(1422) further distinguish this species from all other south-eastern Queensland species.

##### Description.

*Holotype male*: Total length 3.10; leg I femur 3.05; F1/CL ratio 2.77. Cephalothorax dark reddish-brown; legs tan-brown with darker annulations; abdomen mottled grey-brown and beige, palest behind hump-like tubercles, with darker reddish-brown dorsal scute and sclerites (Fig. 17B). Carapace very tall (CH/CL ratio 2.38); 1.10 long, 2.63 high, 1.03 wide; ‘neck’ 0.56 wide; bearing two pairs of rudimentary horns; highest point of pars cephalica (HPC) near posterior third of ‘head’ (ratio of HPC to post-ocular length 0.68), carapace gently sloping and almost horizontal anterior and posterior to HPC; ‘head’ moderately elevated postero-dorsally (post-ocular ratio 0.34) (Fig. 8B). Chelicerae with dense tuft of accessory setae on anterior face of paturon (Fig. 17C). Abdomen 1.67 long, 1.23 wide; with three pairs of dorsal hump-like tubercles (HT 1–6); dorsal scute fused anteriorly to epigastric sclerites, extending posteriorly to first pair of hump-like tubercles; HT 3–6 each covered by separate dorsal sclerites. Unexpanded pedipalp (Figs 17D-F) with broad, distally-directed foliate conductor; tegular sclerite 1 (TS 1) spiniform, widest across middle, obscured by conductor in retrolateral view; TS 2 thin, spiniform, longer than TS 1; TS 2a sinuous, largely obscured by TS 2; TS 3 very large, porrect, with broadly-pointed rectangular apex projecting well beyond retro-distal rim of tegulum.

*Allotype female*: Total length 3.62; leg I femur 3.17; F1/CL ratio 2.40. Cephalothorax tan-brown; legs pale tan-brown with darker annulations; abdomen mottled grey-brown and beige (Figs 5G, 17A). Carapace very tall (CH/CL ratio 2.25); 1.32 long, 2.97 high, 1.18 wide; ‘neck’ 0.62 wide; bearing two pairs of rudimentary horns; highest point of pars cephalica (HPC) near posterior third of ‘head’ (ratio of HPC to post-ocular length 0.64), carapace gently sloping posterior to HPC; ‘head’ moderately elevated postero-dorsally (post-ocular ratio 0.33) (Fig. 7B). Chelicerae without accessory setae on anterior face of paturon. Abdomen 1.85 long, 1.28 wide; with three pairs of dorsal hump-like tubercles (HT 1–6) (Fig. 5G). Internal genitalia with dense cluster of ≤ 15 variably shaped spermathecae on either side of gonopore, clusters meeting near midline of genital plate (Fig. 17G); innermost (anterior) spermathecae longest, sausage-shaped, curved antero-laterally; outermost (posterior) spermathecae bulbous; other spermathecae variably pyriform, straight, directed antero-laterally.

*Variation*: Males (n=3): total length 2.82–3.10; carapace length 1.03–1.10; carapace height 2.35–2.63; CH/CL ratio 2.27–2.44. Females (n=4): total length 3.13–3.62; carapace length 1.26–1.32; carapace height 2.82–2.97; CH/CL ratio 2.25–2.26.

##### Distribution and habitat.

*Austrarchaea aleenae* is known only from rainforest habitats in the Kalpowar-Builyan region of south-eastern Queensland, in the Bulburin National Park and nearby Kalpowar State Forest (Fig. 35).

##### Conservation status.

This species appears to be a short-range endemic taxon (Harvey 2002b), which although potentially restricted in distribution, is abundant within the Bulburin National Park (M. Rix, pers. obs.). It is not considered to be of conservation concern.

#### Austrarchaea alani Kroombit Tops Assassin Spider

Rix & Harvey sp. n.

urn:lsid:zoobank.org:act:C227AAB1-B201-40F7-A94D-4390A30C8518

http://species-id.net/wiki/Austrarchaea_alani

Figs 4A-E 7A 8A 18 36

##### Type material.

Holotype male: Kroombit Tops National Park, creek crossing off Tablelands Road, Queensland, Australia, 24°22'40"S, 150°59'46"E, sifting elevated leaf litter, subtropical rainforest with emergent eucalypts, 799 m, 26.X.2010, M. & A. Rix (QMB S90195).

Paratypes: Allotype female, same data as holotype (QMB S90194); 1 female, same data as holotype (QMB S90196); 2 females and 3 juveniles, same data as holotype (WAM T112550DNA: KT-63-F/KT-64-J/KT-65-J); 1 male and 2 juveniles, Kroombit Tops National Park, Rainforest Walk off Tablelands Road, near Munholme Creek, Queensland, Australia, 24°24'47"S, 151°02'22"E, sifting elevated leaf litter, subtropical rainforest, 753 m, 26.X.2010, M. & A. Rix (WAM T112551DNA: KT-66-M/KT-67-J).

##### Additional material (not examined).

**AUSTRALIA:** Queensland**: Kroombit Tops National Park:** Lower Dry Creek, pitfall trap, rainforest, 13-18.XII.1983, G. Monteith, V. Davies, J. Gallon, G. Thompson, 1♂ (QMB S30812); Three Moon Creek, rainforest, 9-19.XII.1983, V. Davies, J. Gallon, 2♀ (QMB S30816); Beauty Spot 98, rainforest, 9-19.XII.1983, V. Davies, J. Gallon, 1♀ (QMB S30803).

##### Etymology.

The specific epithet is a patronym in honour of Alan Rix, for his great assistance in helping to collect this species, and for a lifetime of generosity and support to the senior author.

##### Diagnosis.

*Austrarchaea alani* can be distinguished from all other Archaeidae from mid-eastern Australia except *Austrarchaea aleenae* by the very large, porrect tegular sclerite 3 (TS 3) (Figs 18D-F); and from *Austrarchaea aleenae* by the short comb of accessory setae on the male chelicerae (Fig. 18C).

This species can also be distinguished from other genotyped taxa from mid-eastern Australia (see Fig. 3B) by the following three unique nucleotide substitutions for COI and COII (n = 5): T(684), A(1218), C(1347).

##### Description.

*Holotype male*: Total length 2.69; leg I femur 2.83; F1/CL ratio 2.68. Cephalothorax reddish-brown; legs tan-brown with darker annulations; abdomen mottled grey-brown and beige, with darker reddish-brown dorsal scute and sclerites (Fig. 18B). Carapace very tall (CH/CL ratio 2.28); 1.06 long, 2.41 high, 0.97 wide; ‘neck’ 0.49 wide; bearing two pairs of rudimentary horns; highest point of pars cephalica (HPC) near posterior margin of ‘head’ (ratio of HPC to post-ocular length 0.85), carapace gently sloping anterior to HPC; ‘head’ moderately elevated postero-dorsally (post-ocular ratio 0.35) (Fig. 8A). Chelicerae with short comb of accessory setae on anterior face of paturon (Fig. 18C). Abdomen 1.38 long, 0.92 wide; with three pairs of dorsal hump-like tubercles (HT 1–6); dorsal scute fused anteriorly to epigastric sclerites, extending posteriorly to first pair of hump-like tubercles; HT 3–6 each covered by separate dorsal sclerites. Unexpanded pedipalp (Figs 18D-F) with broad, obliquely-angled foliate conductor; tegular sclerite 1 (TS 1) relatively short, almost triangular, obscured by conductor in retrolateral view; TS 2 thin, spiniform, longer than TS 1; TS 2a sinuous, largely obscured by TS 2; TS 3 very large, porrect, with broadly-pointed rectangular apex projecting well beyond retro-distal rim of tegulum.

*Allotype female*: Total length 3.41; leg I femur 3.06; F1/CL ratio 2.66. Cephalothorax dark reddish-brown; legs tan-brown with darker annulations; abdomen mottled grey-brown and beige (Fig. 18A). Carapace very tall (CH/CL ratio 2.30); 1.15 long, 2.65 high, 1.04 wide; ‘neck’ 0.56 wide; bearing two pairs of rudimentary horns; highest point of pars cephalica (HPC) near posterior margin of ‘head’ (ratio of HPC to post-ocular length 0.81), carapace sloping gently anterior to HPC; ‘head’ strongly elevated postero-dorsally (post-ocular ratio 0.38) (Fig. 7A). Chelicerae without accessory setae on anterior face of paturon. Abdomen 1.97 long, 1.38 wide; with three pairs of dorsal hump-like tubercles (HT 1–6). Internal genitalia with dense cluster of ≤ 15 variably shaped spermathecae on either side of gonopore, clusters meeting near midline of genital plate (Fig. 18G); innermost (anterior) spermathecae longest, sausage-shaped, curved antero-laterally; outermost (posterior) spermathecae bulbous; other spermathecae variably pyriform, straight, directed antero-laterally.

*Variation*: Males (n=2): total length 2.26–2.69; carapace length 1.03–1.06; carapace height 2.33–2.41; CH/CL ratio 2.28 (invariable). Females (n=4): total length 3.18–3.44; carapace length 1.15–1.18; carapace height 2.58–2.82; CH/CL ratio 2.23–2.39.

##### Distribution and habitat.

*Austrarchaea alani* is known only from rainforest habitats in the Kroombit Tops National Park of south-eastern Queensland (Fig. 36).

**Conservation status.** This species appears to be a short-range endemic taxon (Harvey 2002b), which although potentially restricted in distribution, is abundant within the Kroombit Tops National Park (M. Rix, pers. obs.). It is not considered to be of conservation concern.

### The New South Wales fauna

#### Austrarchaea monteithi Gibraltar Range Assassin Spider

Rix & Harvey sp. n.

urn:lsid:zoobank.org:act:BC7A50E4-6220-4CFC-9A80-7D1EFC20E555

http://species-id.net/wiki/Austrarchaea_monteithi

Figs 5F 7F 9A 19 37

##### Type material.

Holotype male: Gibraltar Range National Park, World Heritage Walk, off Gwydir Highway, near Richardsons Creek, New South Wales, Australia, 29°29'23"S, 152°19'47, sifting elevated leaf litter, subtropical rainforest, 1061 m, 20.IV.2010, M. Rix, D. Harms (AMS KS114977).

Paratype: Allotype female, same data as holotype (AMS KS114976DNA: Ar52-92-F).

##### Other material examined.

**AUSTRALIA:** New South Wales**: Gibraltar Range National Park:** same data as holotype, 2 juveniles (WAM T112570DNA: Ar52-93-J/Ar52-94-J); “Gibraltar Range", pyrethrum, rainforest, 30.III.1980, G. Monteith, 1 juvenile (QMB S30824).

##### Etymology.

The specific epithet is a patronym in honour of Dr Geoff Monteith, for first discovering this species in the Gibraltar Range National Park.

##### Diagnosis.

*Austrarchaea monteithi* can be distinguished from all other Archaeidae from mid-eastern Australia by the presence of only five dorsal hump-like tubercles on the abdomen (Fig. 5F).

This species can also be distinguished from other genotyped taxa from mid-eastern Australia (see Fig. 3B) by the following 29 unique nucleotide substitutions for COI and COII (n = 3): G(81), T(93), A(243), C(300), C(360), C(396), A(597), A(957), C(993), G(1008), C(1115), A(1212), G(1216), T(1217), T(1220), A(1221), G(1229), G(1231), T(1233), G(1275), C(1369), A(1390), G(1391), G(1414), T(1453), G(1509), A(1525), G(1526), G(1554).

##### Description.

**Figure 9.**
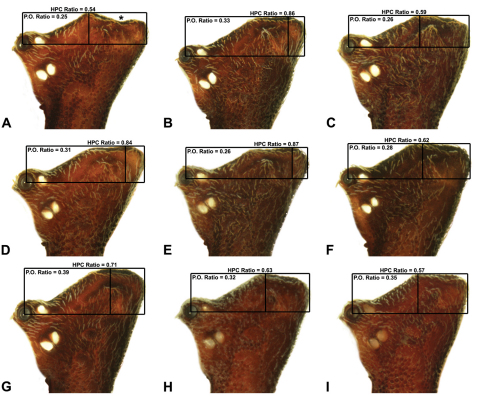
Lateral ‘head’ profiles of males of species of *Austrarchaea* from New South Wales (excluding the Border Ranges), showing variation in carapace shape as quantified by the post-ocular ratio (P.O. Ratio) and ratio of highest point of carapace relative to post-ocular length (HPC Ratio): **A**, holotype *A. monteithi* sp. n.; **B**, holotype *A. christopheri* sp. n.; **C**, holotype *A. platnickorum* sp. n.; **D**, holotype *A. binfordae* sp. n.; **E**, holotype *A. milledgei* sp. n.; **F**, holotype *A. mascordi* sp. n.; **G**, holotype *A. smithae* sp. n.; **H**, holotype *A. mcguiganae* sp. n.; **I**, holotype *A. helenae* sp. n. Asterisks (*) denote concave depressions.

**Figure 10.**
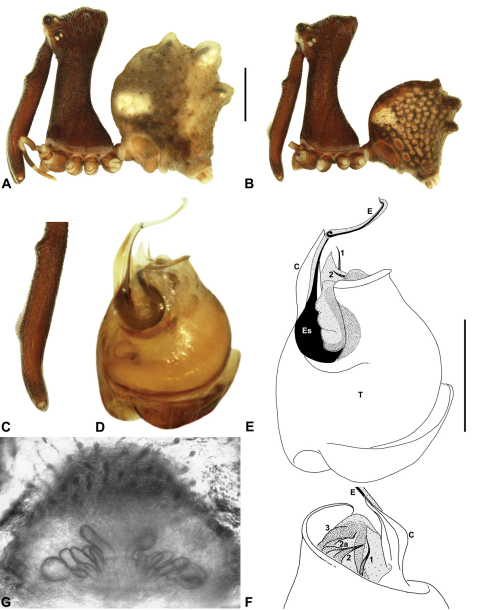
*Austrarchaea nodosa* (Forster, 1956). **A–B**, Cephalothorax and abdomen, lateral view: **A**, female (QMB S75416) from Lamington National Park, Queensland; **B**, male (QMB S75416) from Lamington National Park, Queensland. **C**, Male chelicerae, lateral view, showing accessory setae. **D–F**, Male (WAM T89592) pedipalp: **D–E**, bulb, retrolateral view; **F**, detail of distal tegular sclerites, prodistal view. **G**, Female (QMB S75416) internal genitalia, dorsal view. C = conductor; E = embolus; Es = embolic sclerite; T = tegulum; (TS)1–3 = tegular sclerites 1–3. Scale bars: A–B = 1.0 mm; E = 0.2 mm.

**Figure 11.**
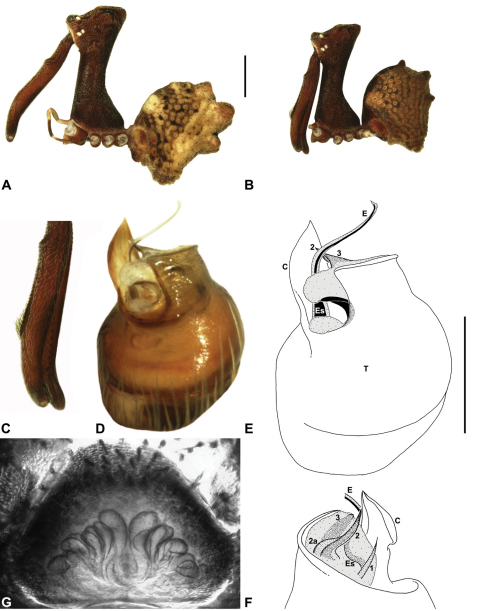
*Austrarchaea dianneae* sp. n. **A–B**, Cephalothorax and abdomen, lateral view: **A**, allotype female (QMB S90186) from Tamborine National Park, Queensland; **B**, holotype male (QMB S90185) from Tamborine National Park, Queensland. **C**, Holotype male chelicerae, lateral view, showing accessory setae. **D–F**, Holotype male pedipalp: **D–E**, bulb, retrolateral view; **F**, detail of distal tegular sclerites, prodistal view. **G**, Allotype female internal genitalia, dorsal view. C = conductor; E = embolus; Es = embolic sclerite; T = tegulum; (TS)1–3 = tegular sclerites 1–3. Scale bars: A–B = 1.0 mm; E = 0.2 mm.

**Figure 12.**
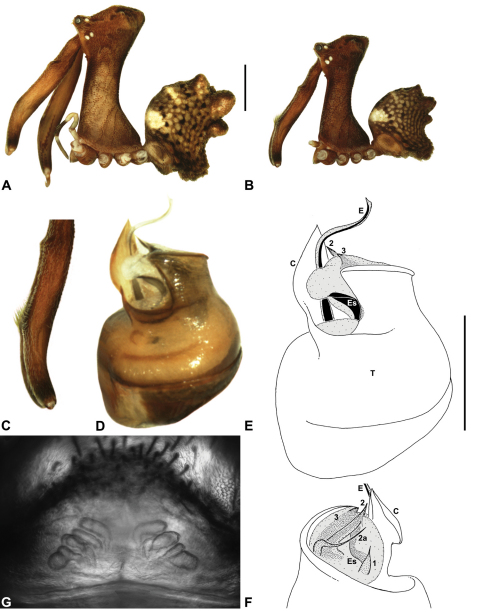
*Austrarchaea cunninghami* sp. n. **A–B**, Cephalothorax and abdomen, lateral view: **A**, allotype female (QMB S90183) from Main Range National Park, Queensland; **B**, holotype male (QMB S90184) from Main Range National Park, Queensland. **C**, Holotype male chelicerae, lateral view, showing accessory setae. **D–F**, Holotype male right pedipalp (flipped horizontal for inter-specific comparison): **D–E**, bulb, retrolateral view; **F**, detail of distal tegular sclerites, prodistal view. **G**, Allotype female internal genitalia, dorsal view. C = conductor; E = embolus; Es = embolic sclerite; T = tegulum; (TS)1–3 = tegular sclerites 1–3. Scale bars: A–B = 1.0 mm; E = 0.2 mm.

**Figure 13.**
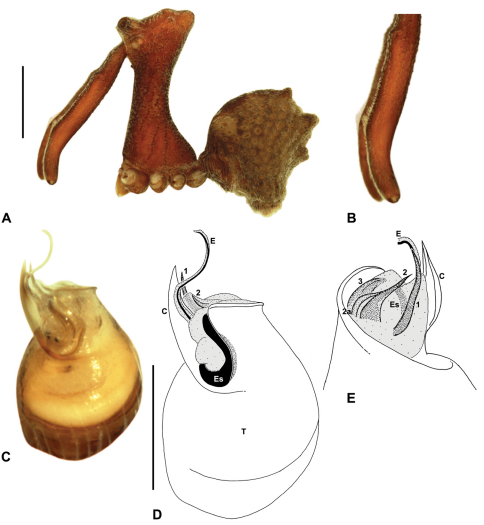
*Austrarchaea clyneae* sp. n. **A–E**, Holotype male (QMB S20425) from Mount Clunie National Park, New South Wales: **A**, cephalothorax and abdomen, lateral view; **B**, chelicerae, lateral view, showing accessory setae; **C–D**, pedipalpal bulb, retrolateral view; **E**, detail of distal tegular sclerites, prodistal view. C = conductor; E = embolus; Es = embolic sclerite; T = tegulum; (TS)1–3 = tegular sclerites 1–3. Scale bars: A = 1.0 mm; D = 0.2 mm.

**Figure 14.**
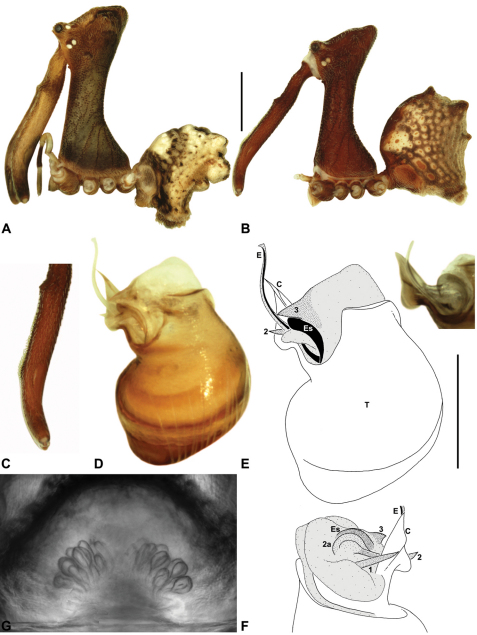
*Austrarchaea raveni* sp. n. **A–B**, Cephalothorax and abdomen, lateral view: **A**, allotype female (QMB S90192) from D’Aguilar National Park, Queensland; **B**, holotype male (QMB S90193) from D’Aguilar National Park, Queensland. **C**, Holotype male chelicerae, lateral view, showing accessory setae. **D–F**, Holotype male pedipalp (partially expanded): **D–E**, bulb, retrolateral view (inset shows conductor and embolus on unexpanded pedipalp of male from Mt Mee Forest Reserve, Queensland); **F**, detail of distal tegular sclerites, prodistal view. **G**, Allotype female internal genitalia, dorsal view. C = conductor; E = embolus; Es = embolic sclerite; T = tegulum; (TS)1–3 = tegular sclerites 1–3. Scale bars: A–B = 1.0 mm; E = 0.2 mm.

**Figure 15.**
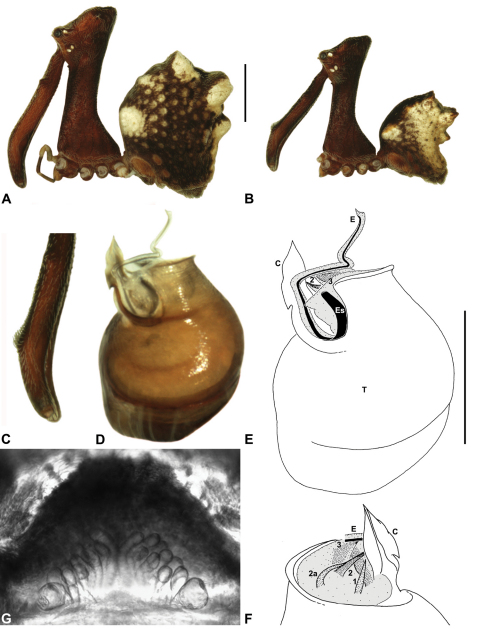
*Austrarchaea judyae* sp. n. **A–B**, Cephalothorax and abdomen, lateral view: **A**, allotype female (QMB S90191) from Conondale National Park, Queensland; **B**, holotype male (QMB S90190) from Conondale National Park, Queensland. **C**, Holotype male chelicerae, lateral view, showing accessory setae. **D–F**, Holotype male pedipalp: **D–E**, bulb, retrolateral view; **F**, detail of distal tegular sclerites, prodistal view. **G**, Allotype female internal genitalia, dorsal view. C = conductor; E = embolus; Es = embolic sclerite; T = tegulum; (TS)1–3 = tegular sclerites 1–3. Scale bars: A–B = 1.0 mm; E = 0.2 mm.

**Figure 16.**
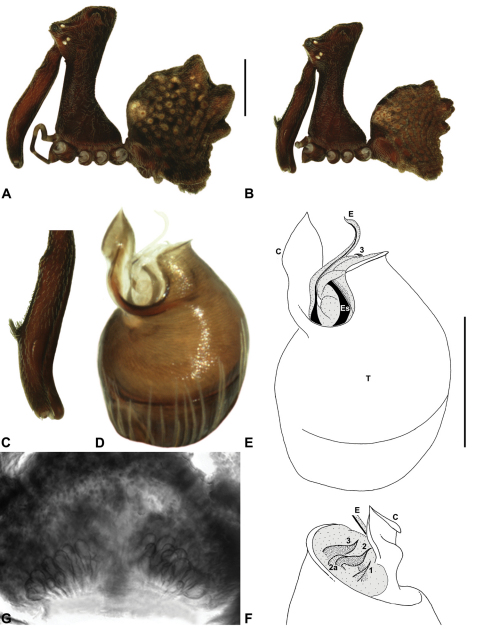
*Austrarchaea harmsi* sp. n. **A–B**, Cephalothorax and abdomen, lateral view: **A**, allotype female (QMB S90187) from Bunya Mountains National Park, Queensland; **B**, holotype male (QMB S90189) from Bunya Mountains National Park, Queensland. **C**, Holotype male chelicerae, lateral view, showing accessory setae. **D–F**, Holotype male pedipalp: **D–E**, bulb, retrolateral view; **F**, detail of distal tegular sclerites, prodistal view. **G**, Allotype female internal genitalia, dorsal view. C = conductor; E = embolus; Es = embolic sclerite; T = tegulum; (TS)1–3 = tegular sclerites 1–3. Scale bars: A–B = 1.0 mm; E = 0.2 mm.

**Figure 17.**
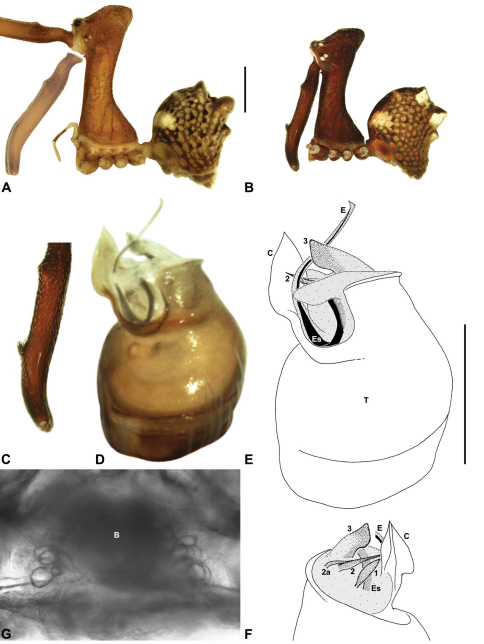
*Austrarchaea aleenae* sp. n. **A–B**, Cephalothorax and abdomen, lateral view: **A**, allotype female (QMB S1094) from Bulburin National Park, Queensland; **B**, holotype male (QMB S90182) from Bulburin National Park, Queensland. **C**, Holotype male chelicerae, lateral view, showing accessory setae. **D–F**, Holotype male pedipalp: **D–E**, bulb, retrolateral view; **F**, detail of distal tegular sclerites, prodistal view. **G**, Allotype female internal genitalia, dorsal view, showing membranous bursa overlying clustered spermathecae. B = bursa; C = conductor; E = embolus; Es = embolic sclerite; T = tegulum; (TS)1–3 = tegular sclerites 1–3. Scale bars: A–B = 1.0 mm; E = 0.2 mm.

**Figure 18.**
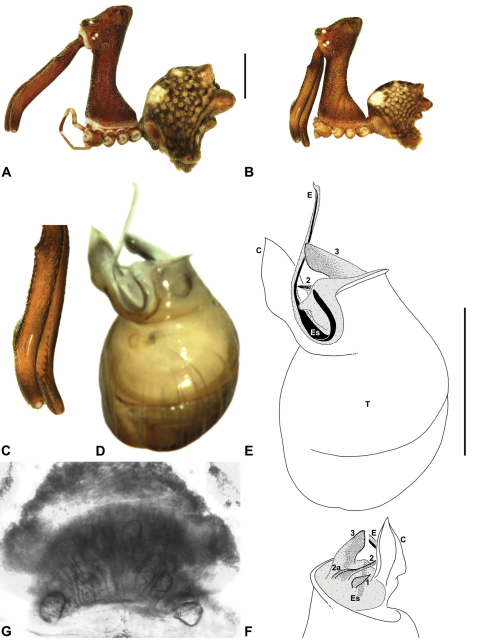
*Austrarchaea alani* sp. n. **A–B**, Cephalothorax and abdomen, lateral view: **A**, allotype female (QMB S90194) from Kroombit Tops National Park, Queensland; **B**, holotype male (QMB S90195) from Kroombit Tops National Park, Queensland. **C**, Holotype male chelicerae, lateral view, showing accessory setae. **D–F**, Holotype male pedipalp: **D–E**, bulb, retrolateral view; **F**, detail of distal tegular sclerites, prodistal view. **G**, Allotype female internal genitalia, dorsal view. C = conductor; E = embolus; Es = embolic sclerite; T = tegulum; (TS)1–3 = tegular sclerites 1–3. Scale bars: A–B = 1.0 mm; E = 0.2 mm.

**Figure 19.**
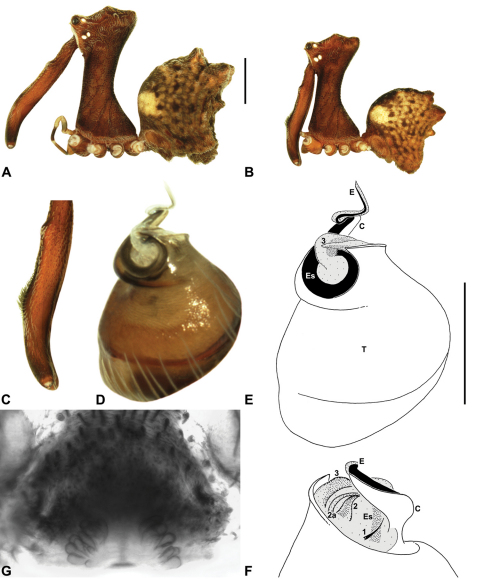
*Austrarchaea monteithi* sp. n. **A–B**, Cephalothorax and abdomen, lateral view: **A**, allotype female (AMS KS114976) from Gibralter Range National Park, New South Wales; **B**, holotype male (AMS KS114977) from Gibralter Range National Park, New South Wales. **C**, Holotype male chelicerae, lateral view, showing accessory setae. **D–F**, Holotype male pedipalp: **D–E**, bulb, retrolateral view; **F**, detail of distal tegular sclerites, prodistal view. **G**, Allotype female internal genitalia, dorsal view. C = conductor; E = embolus; Es = embolic sclerite; T = tegulum; (TS)1–3 = tegular sclerites 1–3. Scale bars: A–B = 1.0 mm; E = 0.2 mm.

*Holotype male*: Total length 3.13; leg I femur 2.91; F1/CL ratio 2.58. Cephalothorax dark reddish-brown; legs tan-brown with darker annulations; abdomen mottled grey-brown and beige, with darker reddish-brown dorsal scute and sclerites (Fig. 19B). Carapace tall (CH/CL ratio 2.07); 1.13 long, 2.33 high, 1.05 wide; ‘neck’ 0.53 wide; bearing two pairs of rudimentary horns; highest point of pars cephalica (HPC) near middle of ‘head’ (ratio of HPC to post-ocular length 0.54), carapace with concave depression posterior to HPC; ‘head’ not strongly elevated dorsally (post-ocular ratio 0.25) (Fig. 9A). Chelicerae with short brush of accessory setae on anterior face of paturon (Fig. 19C). Abdomen 1.69 long, 1.10 wide; with five dorsal hump-like tubercles (HT 1–5), HT1–4 arranged in two pairs; dorsal scute fused anteriorly to epigastric sclerites, extending posteriorly to first pair of hump-like tubercles; HT 3–5 each covered by separate dorsal sclerites. Unexpanded pedipalp (Figs 19D-F) with stout, almost spherical bulb and thin, strongly hooked conductor; embolic sclerite with broad, looped proximal portion extending for entire length of conductor; tegular sclerite 1 (TS 1) relatively short, filiform, obscured by conductor in retrolateral view; TS 2 spur-like, longer than TS 1; TS 2a sinuous, largely obscured by TS 2; TS 3 embedded proximally within distal haematodocha, with sharply-pointed apex projecting ventrally beyond retro-distal rim of tegulum.

*Allotype female*: Total length 3.38; leg I femur 3.08; F1/CL ratio 2.31. Cephalothorax dark reddish-brown; legs tan-brown with darker annulations; abdomen mottled grey-brown and beige (Fig. 19A). Carapace tall (CH/CL ratio 2.12); 1.33 long, 2.82 high, 1.21 wide; ‘neck’ 0.66 wide; bearing two pairs of rudimentary horns; highest point of pars cephalica (HPC) near middle of ‘head’ (ratio of HPC to post-ocular length 0.55), carapace with concave depression posterior to HPC; ‘head’ not strongly elevated dorsally (post-ocular ratio 0.23) (Fig. 7F). Chelicerae without accessory setae on anterior face of paturon. Abdomen 1.85 long, 1.44 wide; with five dorsal hump-like tubercles (HT 1–5), HT1–4 arranged in two pairs (Fig. 5F). Internal genitalia with cluster of ≤ 12 variably shaped spermathecae on either side of gonopore, clusters widely separated along midline of genital plate (Fig. 19G); innermost (anterior) spermathecae longest; other spermathecae variably pyriform, straight, directed antero-laterally.

##### Distribution and habitat.

*Austrarchaea monteithi* is known only from subtropical rainforest habitats in the Gibraltar Range National Park of north-eastern New South Wales (Fig. 37).

##### Conservation status.

This enigmatic species has an imperfectly known distribution, and although potentially restricted, appears to be relatively abundant within the World Heritage-listed Gibraltar Range National Park near Richardsons Creek (M. Rix, pers. obs.). It is not considered to be of conservation concern.

#### Austrarchaea christopheri Dorrigo Assassin Spider

Rix & Harvey sp. n.

urn:lsid:zoobank.org:act:EF9D764D-FA0B-4473-BBD3-B31D30AF251D

http://species-id.net/wiki/Austrarchaea_christopheri

Figs 9B 20 38

##### Type material.

Holotype male: Dorrigo National Park, Rosewood Creek Circuit track from The Never Never Picnic Area, New South Wales, Australia, 30°21'42"S, 152°47'55"E, sifting elevated leaf litter, subtropical rainforest, 1092 m, 17.IV.2010, M. Rix, D. Harms (AMS KS114968DNA: Ar49-95-M).

Paratypes: 2 males and 4 juveniles, same data as holotype (WAM T112554DNA: Ar49-96-J/Ar49-97-J).

##### Other material examined.

**AUSTRALIA:** New South Wales**: Dorrigo National Park:** The Never Never, III.–12.XI.1980, G. Monteith, 1♂ (QMB S30806). **Cascades National Park:** off Briggsvale Road, N. of Megan, 30°15'11"S, 152°46'52"E, sifting elevated leaf litter, subtropical rainforest, 848 m, 17.IV.2010, M. Rix, D. Harms, 3 juveniles (WAM T112553DNA: Ar50-98-J/Ar50-99-J/Ar50-100-J). **New England National Park:** “Oakes State Forest", Horseshoe Road, ~1.2 km S. of Killiekrankie Mountain, 30°33'10"S, 152°32'15"E, pitfall trap, 11-24.XI.1999, M. Gray, G. Milledge, H. Smith, 1♂ (AMS KS61544).

##### Etymology.

The specific epithet is a patronym in honour of Christopher Rix, for his close association with the Dorrigo region, and for his great achievements, both personal and professional.

##### Diagnosis.

**Figure 20.**
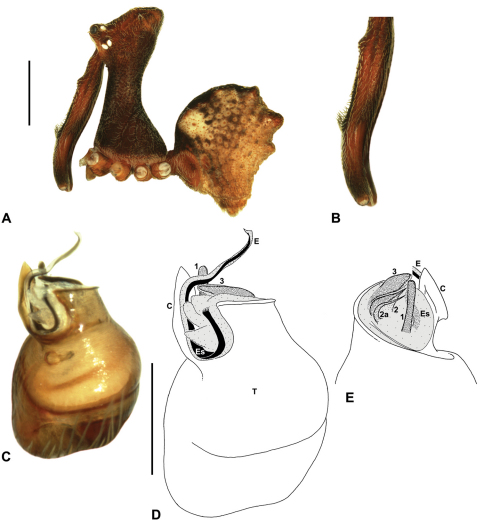
*Austrarchaea christopheri* sp. n. **A–E**, Holotype male (AMS KS114968) from Dorrigo National Park, New South Wales: **A**, cephalothorax and abdomen, lateral view; **B**, chelicerae, lateral view, showing accessory setae; **C–D**, pedipalpal bulb, retrolateral view; **E**, detail of distal tegular sclerites, prodistal view. C = conductor; E = embolus; Es = embolic sclerite; T = tegulum; (TS)1–3 = tegular sclerites 1–3. Scale bars: A = 1.0 mm; D = 0.2 mm.

*Austrarchaea christopheri* can be distinguished from all other Archaeidae from mid-eastern Australia by the very long, uniquely rod-like tegular sclerite 1 (TS 1) (Figs 20D-E).

This species can also be distinguished from other genotyped taxa from mid-eastern Australia (see Fig. 3B) by the following four unique nucleotide substitutions for COI and COII (n = 6): A(63), C(801), A(1070), G(1332).

##### Description.

*Holotype male*: Total length 3.17; leg I femur 2.96; F1/CL ratio 2.57. Cephalothorax dark reddish-brown; legs tan-brown with darker annulations; abdomen mottled grey-brown and beige, with darker reddish-brown dorsal scute and sclerites (Fig. 20A). Carapace tall (CH/CL ratio 2.10); 1.15 long, 2.42 high, 1.08 wide; ‘neck’ 0.54 wide; bearing two pairs of rudimentary horns; highest point of pars cephalica (HPC) near posterior margin of ‘head’ (ratio of HPC to post-ocular length 0.86), carapace gently sloping and almost horizontal anterior to HPC; ‘head’ moderately elevated postero-dorsally (post-ocular ratio 0.33) (Fig. 9B). Chelicerae with brush of accessory setae on anterior face of paturon (Fig. 20B). Abdomen 1.64 long, 1.17 wide; with three pairs of dorsal hump-like tubercles (HT 1–6); dorsal scute fused anteriorly to epigastric sclerites, extending posteriorly to first pair of hump-like tubercles; HT 3–6 each covered by separate dorsal sclerites. Unexpanded pedipalp (Figs 20C-E) with thin, broadly-tapered foliate conductor; tegular sclerite 1 (TS 1) very long, rod-like, reaching to near distal tip of conductor, visible in retrolateral view; TS 2 spur-like, shorter than TS 1; TS 2a sinuous, filiform, exposed distally; TS 3 embedded proximally within distal haematodocha, with prominent, pointed apex projecting beyond retro-distal rim of tegulum.

*Female*: Unknown.

*Variation*: Males (n=5): total length 2.72–3.23; carapace length 1.09–1.15; carapace height 2.31–2.46; CH/CL ratio 2.07–2.18.

##### Distribution and habitat.

*Austrarchaea christopheri* is known from rainforest habitats throughout the Dorrigo and New England hinterland of north-eastern New South Wales (west and south-west of Coffs Harbour), in the Dorrigo, Cascades and New England National Parks (Fig. 38).

##### Conservation status.

This species has a relatively widespread distribution in several National Parks protected under World Heritage legislation, and is not considered to be of conservation concern.

#### Austrarchaea platnickorum New England Assassin Spider

Rix & Harvey sp. n.

urn:lsid:zoobank.org:act:95A5C68E-D47C-44B0-BE6A-26559C286359

http://species-id.net/wiki/Austrarchaea_platnickorum

Figs 7J 9C 21 39

##### Type material.

Holotype male: New England National Park, Banksia Point, Beech Forest and start of Lyrebird Track, New South Wales, Australia, 30°29'29"S, 152°24'22"E, sifting elevated leaf litter under tussocky snow grass, *Nothofagus* rainforest and adjacent snow gum woodland, 1491 m, 18.IV.2010, M. Rix, D. Harms (AMS KS114971).

Paratypes: Allotype female, same data as holotype (AMS KS114970); 3 males, 2 females and 5 juveniles, same data as holotype (WAM T112558DNA: Ar51-101-M/Ar51-102-F/Ar51-103-J).

##### Other material examined.

**AUSTRALIA:** New South Wales**: New England National Park:** Banksia Point, ex pan traps, 2-15.X.1984, I. Naumann, J. Cardale, 1 juvenile (ANIC); Point Lookout, 22.III.1980 – 16.III.1981, G. Monteith, 1 juvenile (QMB S30819).

##### Etymology.

The specific epithet is a patronym in honour of Dr Norman Platnick and his wife Nancy. Dr Platnick’s pioneering research into many different spider lineages – including Archaeidae – has inspired a generation of arachnologists.

##### Diagnosis.

**Figure 21.**
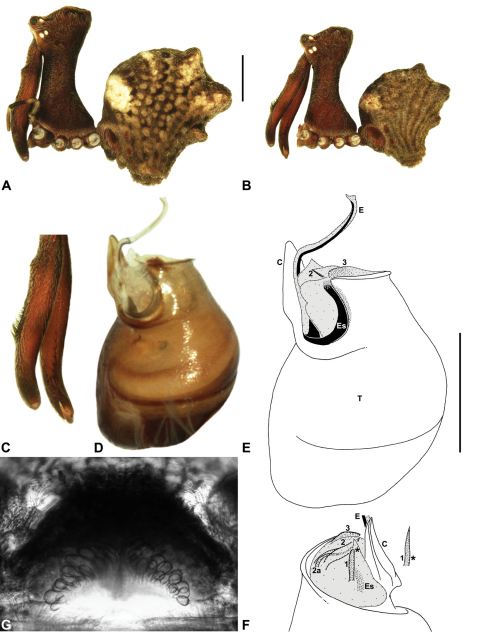
*Austrarchaea platnickorum* sp. n. **A–B**, Cephalothorax and abdomen, lateral view: **A**, allotype female (AMS KS114970) from New England National Park, New South Wales; **B**, holotype male (AMS KS114971) from New England National Park, New South Wales. **C**, Holotype male chelicerae, lateral view, showing accessory setae. **D–F**, Holotype male pedipalp: **D–E**, bulb, retrolateral view; **F**, detail of distal tegular sclerites, prodistal view. **G**, Allotype female internal genitalia, dorsal view. Note the broken left tegular sclerite 1 (TS 1) in (F), highlighted (*) at the point of breakage, compared to the long, sharply-pointed right TS 1 (see inset). C = conductor; E = embolus; Es = embolic sclerite; T = tegulum; (TS)1–3 = tegular sclerites 1–3. Scale bars: A–B = 1.0 mm; E = 0.2 mm.

*Austrarchaea platnickorum* can be distinguished from all other Archaeidae from mid-eastern Australia by the very long, spiniform tegular sclerite 1 (TS 1) (Fig. 21F) combined with the unique shape of the conductor (Figs 21D-E), which is thin and ‘arrow-shaped’, with a long triangular apex.

This species can also be distinguished from other genotyped taxa from mid-eastern Australia (see Fig. 3B) by the following eight unique nucleotide substitutions for COI and COII (n = 3): A(354), A(573), A(624), T(986), G(1061), G(1077), C(1110), T(1533).

##### Description.

*Holotype male*: Total length 3.28; leg I femur 2.67; F1/CL ratio 2.31. Cephalothorax dark reddish-brown; legs tan-brown with darker annulations; abdomen mottled grey-brown and beige, with darker reddish-brown dorsal scute and sclerites (Fig. 21B). Carapace tall (CH/CL ratio 2.07); 1.15 long, 2.38 high, 1.08 wide; ‘neck’ 0.59 wide; bearing two pairs of rudimentary horns; highest point of pars cephalica (HPC) near middle of ‘head’ (ratio of HPC to post-ocular length 0.59), carapace gently sloping posterior to HPC; ‘head’ not strongly elevated dorsally (post-ocular ratio 0.26) (Fig. 9C). Chelicerae with brush of accessory setae on anterior face of paturon (Fig. 21C). Abdomen 1.85 long, 1.41 wide; with three pairs of dorsal hump-like tubercles (HT 1–6); dorsal scute fused anteriorly to epigastric sclerites, extending posteriorly to first pair of hump-like tubercles; HT 3–6 each covered by separate dorsal sclerites. Unexpanded pedipalp (Figs 21D-F) with thin, triangular ‘arrow-shaped’ conductor; tegular sclerite 1 (TS 1) very long, spiniform, visible in retrolateral view (TS 1 broken, rod-like on left pedipalp; Fig. 21F); TS 2 spur-like, poorly-sclerotised, longer than TS 1; TS 2a sinuous, largely obscured by TS 2; TS 3 indistinct, embedded within distal haematodocha, barely visible beyond retro-distal rim of tegulum.

*Allotype female*: Total length 4.31; leg I femur 2.79; F1/CL ratio 2.14. Cephalothorax dark reddish-brown; legs tan-brown with darker annulations; abdomen mottled grey-brown and beige (Fig. 21A). Carapace tall (CH/CL ratio 2.04); 1.31 long, 2.67 high, 1.21 wide; ‘neck’ 0.69 wide; bearing two pairs of rudimentary horns; highest point of pars cephalica (HPC) near middle of ‘head’ (ratio of HPC to post-ocular length 0.60), carapace gently sloping posterior to HPC; ‘head’ not strongly elevated dorsally (post-ocular ratio 0.27) (Fig. 7J). Chelicerae without accessory setae on anterior face of paturon. Abdomen 2.72 long, 1.95 wide; with three pairs of dorsal hump-like tubercles (HT 1–6). Internal genitalia with dense cluster of ≤ 20 variably shaped spermathecae on either side of gonopore, clusters meeting near midline of genital plate (Fig. 21G); innermost (anterior) spermathecae longest, sausage-shaped, curved antero-laterally; other spermathecae variably aciniform, mostly straight, directed antero-laterally.

*Variation*: Males (n=4): total length 2.97–3.28; carapace length 1.10–1.15; carapace height 2.21–2.38; CH/CL ratio 2.00–2.07. Females (n=3): total length 3.79–4.62; carapace length 1.26–1.31; carapace height 2.54–2.67; CH/CL ratio 2.02–2.12. The holotype male and an additional paratype male (WAM T112558) of this species have a shorter, partially broken tegular sclerite 1 (TS 1) on each left pedipalp (Fig. 21F).

##### Distribution and habitat.

*Austrarchaea platnickorum* is known only from rainforest and mesic closed forest habitats in the New England National Park of north-eastern New South Wales (Fig. 39).

##### Conservation status.

This species has an imperfectly known distribution, and although potentially restricted, appears to be abundant within the World Heritage-listed New England National Park near Point Lookout (M. Rix, pers. obs.). It is not considered to be of conservation concern.

#### Austrarchaea binfordae Kerewong Assassin Spider

Rix & Harvey sp. n.

urn:lsid:zoobank.org:act:0AE5EF85-E215-48F7-B443-B0B4E144238F

http://species-id.net/wiki/Austrarchaea_binfordae

Figs 7K 9D 22 40

##### Type material.

Holotype male: Kerewong State Forest, off McLeods Creek Road, New South Wales, Australia, 31°33'39"S, 152°34'44"E, sifting elevated leaf litter, subtropical rainforest, 15.IV.2010, M. Rix, D. Harms (AMS KS114969DNA: Ar46-106-M).

Paratypes: Allotype female, Kerewong State Forest, New South Wales, Australia, 28.XI.1978, D. Milledge (AMS KS13891); 1 male, same data (AMS KS13891).

##### Other material examined.

**AUSTRALIA:** New South Wales**: Lorne State Forest:** “Lorne State Forest", pitfall trap, 4.XI.1979, D. Milledge, 1 juvenile (AMS KS5624); same data except 5.VII.1979, D. Milledge, 1 juvenile (AMS KS5390).

##### Additional material examined (of tentative identification).

**AUSTRALIA**. New South Wales**: Willi Willi National Park:** Banda Banda Antarctic Beech Forest, off Banda Road, 31°09'47"S, 152°24'23"E, sifting elevated leaf litter, *Nothofagus* rainforest, 1045 m, 16.IV.2010, M. Rix, D. Harms, 2 juveniles (WAM T112580DNA: Ar47-104-J/Ar47-105-J).

##### Etymology.

The specific epithet is a patronym in honour of Dr Greta Binford, for her pioneering research on spider venoms and for contributing to a highly successful basal clades tour.

##### Diagnosis.

**Figure 22.**
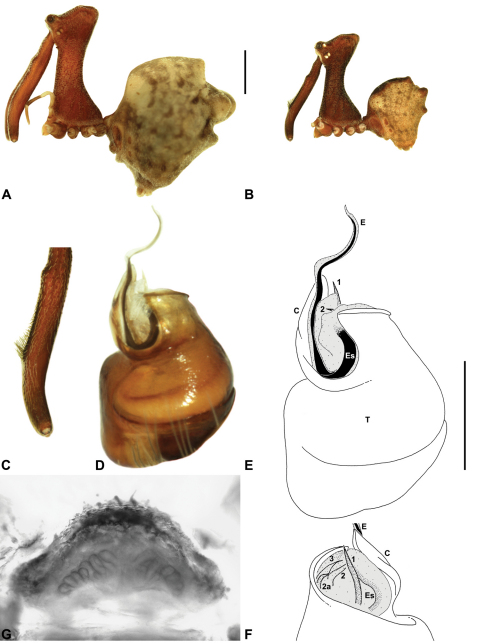
*Austrarchaea binfordae* sp. n. **A–B**, Cephalothorax and abdomen, lateral view: **A**, allotype female (AMS KS13891) from Kerewong State Forest, New South Wales; **B**, holotype male (AMS KS114969) from Kerewong State Forest, New South Wales. **C**, Holotype male chelicerae, lateral view, showing accessory setae. **D–F**, Holotype male pedipalp: **D–E**, bulb, retrolateral view; **F**, detail of distal tegular sclerites, prodistal view. **G**, Allotype female internal genitalia, dorsal view. C = conductor; E = embolus; Es = embolic sclerite; T = tegulum; (TS)1–3 = tegular sclerites 1–3. Scale bars: A–B = 1.0 mm; E = 0.2 mm.

*Austrarchaea binfordae* can be distinguished from all other Archaeidae from mid-eastern Australia by the very long, spiniform tegular sclerite 1 (TS 1) (Fig. 22F) combined with the unique shape of the conductor (Figs 22D-E), which is thin and slightly curved laterally, with a ridged ventral margin.

This species can also be distinguished from other genotyped taxa from mid-eastern Australia (see Fig. 3B) by the following 14 unique nucleotide substitutions for COI and COII (n = 1): C(291), C(369), C(489), C(720), C(807), T(1013), A(1014), A(1018), C(1019), A(1177), G(1214), C(1294), C(1312), G(1563).

##### Description.

*Holotype male*: Total length 2.64; leg I femur 2.63; F1/CL ratio 2.70. Cephalothorax dark reddish-brown; legs tan-brown with darker annulations; abdomen mottled grey-brown and beige, with darker reddish-brown dorsal scute and sclerites (Fig. 22B). Carapace tall (CH/CL ratio 2.16); 0.97 long, 2.10 high, 0.92 wide; ‘neck’ 0.44 wide; bearing two pairs of rudimentary horns; highest point of pars cephalica (HPC) near posterior margin of ‘head’ (ratio of HPC to post-ocular length 0.84), carapace gently sloping and almost horizontal anterior to HPC; ‘head’ not strongly elevated dorsally (post-ocular ratio 0.31) (Fig. 9D). Chelicerae with brush of accessory setae on anterior face of paturon (Fig. 22C). Abdomen 1.38 long, 1.05 wide; with three pairs of dorsal hump-like tubercles (HT 1–6); dorsal scute fused anteriorly to epigastric sclerites, extending posteriorly to first pair of hump-like tubercles; HT 3–6 each covered by separate dorsal sclerites. Unexpanded pedipalp (Figs 22D-F) with thin, slightly curved conductor with ridged ventral margin; tegular sclerite 1 (TS 1) very long, spiniform, visible in retrolateral view; TS 2 spur-like, poorly-sclerotised, shorter than TS 1; TS 2a sinuous, largely obscured by TS 2; TS 3 indistinct, embedded within distal haematodocha, barely visible beyond retro-distal rim of tegulum.

*Allotype female*: Total length 4.15; leg I femur 2.82; F1/CL ratio 2.39. Cephalothorax reddish-brown; legs tan-brown with darker annulations; abdomen mottled grey-brown and beige (Fig. 22A). Carapace tall (CH/CL ratio 2.19); 1.18 long, 2.58 high, 1.08 wide; ‘neck’ 0.59 wide; bearing two pairs of rudimentary horns; highest point of pars cephalica (HPC) near posterior third of ‘head’ (ratio of HPC to post-ocular length 0.64), carapace gently sloping posterior to HPC; ‘head’ not strongly elevated dorsally (post-ocular ratio 0.28) (Fig. 7K). Chelicerae without accessory setae on anterior face of paturon. Abdomen 2.62 long, 2.21 wide; with three pairs of dorsal hump-like tubercles (HT 1–6). Internal genitalia with cluster of ≤ 12 variably shaped spermathecae on either side of gonopore, clusters meeting near midline of genital plate (Fig. 21G); innermost (anterior) spermathecae longest, sausage-shaped, curved antero-laterally; other spermathecae variably pyriform, straight, directed antero-laterally.

*Variation*: Males (n=2): total length 2.64–2.92; carapace length 0.97–1.03; carapace height 2.10–2.18; CH/CL ratio 2.13–2.16.

##### Distribution and habitat.

*Austrarchaea binfordae* is known only from lowland subtropical rainforest habitats in the Kerewong and Lorne State Forests, near Wauchope, New South Wales (Fig. 40). Two juvenile specimens from Mount Banda Banda (Willi Willi National Park) may also belong to this species, but possess divergent mtDNA sequences indicative of possible speciation (Fig. 3B).

##### Conservation status.

This species appears to be a rare short-range endemic taxon (Harvey 2002b), with populations in the Kerewong and Lorne State Forests **potentially threatened** by land-clearing, habitat degradation, fire and climate change. It is one of the few archaeids known to occur in lowland rainforest habitats in south-eastern Australia, and many of these habitats have been severely impacted by forestry activities.

#### Austrarchaea milledgei Barrington Tops Assassin Spider

Rix & Harvey sp. n.

urn:lsid:zoobank.org:act:BE4BD907-11B1-4CCA-AD3A-590B2FC9F3AE

http://species-id.net/wiki/Austrarchaea_milledgei

Figs 7L 9E 23 41

##### Type material.

Holotype male: Barrington Tops State Forest, 0.8 km E. of Moppy Picnic Area, New South Wales, Australia, 31°53'22"S, 151°33'57"E, pitfall trap, *Nothofagus* rainforest, 1250 m, 18.III.–30.IV.2008, G. Milledge, A. Hegedus (AMS KS103905).

##### Other material examined.

**AUSTRALIA:** New South Wales**: Barrington Tops State Forest:** same data as holotype except 31.I.–18.III.2008, 1 juvenile (AMS KS103409); same data as holotype except 18.XII.2007 – 31.I.2008, 1 juvenile (AMS KS102056); same data as holotype except 14.XI.–18.XII.2007, G. Milledge, H. Smith, 1 juvenile (AMS KS104681); same data, 1 juvenile (AMS KS104696); off Barrington Tops Forest Road, 31°54'45"S, 151°29'45, sifting elevated leaf litter under tree ferns, *Nothofagus* rainforest, 1400 m, 14.IV.2010, M. Rix, D. Harms, 8 juveniles (WAM T112569DNA: Ar42-110-J/Ar42-111-J/Ar42-112-J). **Barrington Tops National Park:** Quarry Road turnoff, 31°54'45"S, 151°31'10"E, pitfall trap, *Nothofagus* rainforest, 1230 m, 31.I.–18.III.2008, G. Milledge, A. Hegedus, 1♂, 1 juvenile (AMS KS103340); same data except 18.XII.2007 – 31.I.2008, 1 juvenile (AMS KS102013); off Barrington Tops Forest Road, 31°54'22"S, 151°31'32, sifting elevated leaf litter, complex eucalypt forest with thick understory, 1376 m, 14.IV.2010, M. Rix, D. Harms, 1♀, 3 juveniles (WAM T112568DNA: Ar43-107-F/Ar43-108-J/Ar43-109-J).

##### Additional material examined (of tentative identification).

**AUSTRALIA:** New South Wales**: Barrington Tops National Park:** Gloucester Tops Road, 12.1 km W. of Gloucester River campground, 32°03'45"S, 151°36'02"E, pitfall trap, *Nothofagus* rainforest, 1260 m, 13.XI.–19.XII.2007, G. Milledge, H. Smith, 1♀ (AMS KS102950). **Chichester State Forest:** Bungari Road, 1 km from Mount Allyn Road, 32°08'S, 151°26'E, 940 m, 4.II–9.IV.1993, M. Gray, G. Cassis, 1 juvenile (AMS KS38978).

##### Etymology.

The specific epithet is a patronym in honour of Graham Milledge, for collecting most of the specimens of this species, and for his efforts in documenting the remarkable spider fauna of the Barrington Tops region of New South Wales.

##### Diagnosis.

**Figure 23.**
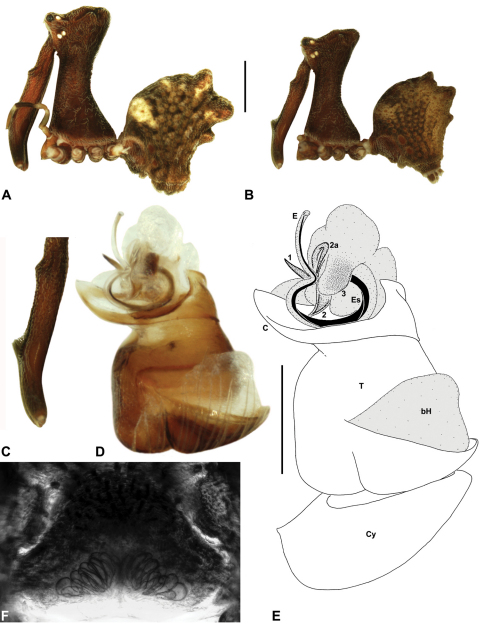
*Austrarchaea milledgei* sp. n. **A–B**, Cephalothorax and abdomen, lateral view: **A**, female (WAM T112568) from Barrington Tops National Park, New South Wales; **B**, holotype male (AMS KS103905) from Barrington Tops State Forest, New South Wales. **C**, Holotype male chelicerae, lateral view, showing accessory setae. **D–E**, Holotype male pedipalpal bulb (expanded), retro-ventral view. **F**, Female (WAM T112568) internal genitalia, dorsal view. bH = basal haematodocha; C = conductor; Cy = cymbium; E = embolus; Es = embolic sclerite; T = tegulum; (TS)1–3 = tegular sclerites 1–3. Scale bars: A–B = 1.0 mm; E = 0.2 mm.

*Austrarchaea milledgei* can be distinguished from all other Archaeidae from mid-eastern Australia except *Austrarchaea aleenae* by the short, dense tuft of accessory setae on the male chelicerae (Fig. 23C); and from *Austrarchaea aleenae* by the broader, spur-like tegular sclerite 2 (TS 2) and much smaller TS 3 (Fig. 23E).

This species can also be distinguished from other genotyped taxa from mid-eastern Australia (see Fig. 3B) by the following unique nucleotide substitution for COII (n = 6): C(1490). The COII substitution T(1511) further distinguishes this species from all other mid-eastern Australian taxa except *Austrarchaea monteithi*.

##### Description.

*Holotype male*: Total length 3.08; leg I femur 2.73; F1/CL ratio 2.51. Cephalothorax dark reddish-brown; legs tan-brown with darker annulations; abdomen mottled grey-brown and beige, palest posteriorly, with darker reddish-brown dorsal scute and sclerites (Fig. 23B). Carapace tall (CH/CL ratio 2.12); 1.09 long, 2.31 high, 1.05 wide; ‘neck’ 0.53 wide; bearing two pairs of rudimentary horns; highest point of pars cephalica (HPC) near posterior margin of ‘head’ (ratio of HPC to post-ocular length 0.87), carapace gently sloping and almost horizontal anterior to HPC; ‘head’ not strongly elevated dorsally (post-ocular ratio 0.26) (Fig. 9E). Chelicerae with dense tuft of accessory setae on anterior face of paturon (Fig. 23C). Abdomen 1.54 long, 1.13 wide; with three pairs of dorsal hump-like tubercles (HT 1–6); dorsal scute fused anteriorly to epigastric sclerites, extending posteriorly to first pair of hump-like tubercles; HT 3–6 each covered by separate dorsal sclerites. Partially expanded pedipalp (Figs 23D-E) with rounded-rectangular, broadly-tapered conductor; tegular sclerite 1 (TS 1) long, spiniform, visible in retro-ventral view; TS 2 spur-like, shorter than TS 1, partially obscured by TS 3; TS 2a sinuous, filiform, exposed distally; TS 3 rectangular, embedded within distal haematodocha, overlying proximal embolic sclerite and TS 2.

*Female* (WAM T112568): Total length 3.74; leg I femur 2.96; F1/CL ratio 2.24. Cephalothorax dark reddish-brown; legs tan-brown with darker annulations; abdomen mottled grey-brown and beige (Fig. 23A). Carapace tall (CH/CL ratio 2.02); 1.32 long, 2.67 high, 1.23 wide; ‘neck’ 0.65 wide; bearing two pairs of rudimentary horns; highest point of pars cephalica (HPC) near middle of ‘head’ (ratio of HPC to post-ocular length 0.59), carapace gently sloping posterior to HPC; ‘head’ not strongly elevated dorsally (post-ocular ratio 0.21) (Fig. 7L). Chelicerae without accessory setae on anterior face of paturon. Abdomen 2.10 long, 1.41 wide; with three pairs of dorsal hump-like tubercles (HT 1–6). Internal genitalia with dense cluster of ≤ 15 variably shaped spermathecae on either side of gonopore, clusters meeting near midline of genital plate (Fig. 23F); innermost (anterior) spermathecae longest, sausage-shaped, curved antero-laterally; outermost (posterior) spermathecae bulbous; other spermathecae variably pyriform, mostly straight, directed antero-laterally.

*Variation*: Males (n=2): total length 3.08–3.23; carapace length 1.09–1.10; carapace height 2.21–2.31; CH/CL ratio 2.00–2.12. For female variation see Remarks (below).

##### Distribution and habitat.

*Austrarchaea milledgei* is known only from rainforest and mesic closed forest habitats on the Barrington Tops Plateau, in the Barrington Tops National Park and Barrington Tops State Forest, New South Wales (Fig. 41). A juvenile specimen from Chichester State Forest and a single female specimen from Gloucester Tops may also belong to this species (see Remarks, below).

##### Conservation status.

This species is a short-range endemic taxon (Harvey 2002b), which although restricted in distribution, is abundant within the World Heritage-listed Barrington Tops National Park (M. Rix, pers. obs.). It is not considered to be of conservation concern.

##### Remarks.

Specimens of *Austrarchaea* from the Barrington Tops Plateaux (i.e. Barrington Tops, Gloucester Tops and Chichester State Forest) seem to exhibit a larger than normal amount of morphological and molecular variation, as highlighted by the deep (~6%) COI and COII sequence divergences observed among specimens collected along the Barrington Tops Forest Road in April 2010 (Fig. 3A), and the incongruous CH/CL ratio of the single female (AMS KS102950) from Gloucester Tops relative to the only known female from Barrington Tops (WAM T112568) (see Fig. 6). It is possible that two cryptic species may occur in sympatry on the various mountains and plateaux that make up the Barrington Tops region, although the current paucity of specimens makes this difficult to ascertain. For the purposes of this revision, specimens from the northern Barrington Tops National Park (and State Forest) are recognised as conspecific with the holotype of *Austrarchaea milledgei*, but further work is required to compare males from across the region, and to confirm the identification of the Gloucester Tops and Chichester State Forest populations.

#### Austrarchaea mascordi Coolah Tops Assassin Spider

Rix & Harvey sp. n.

urn:lsid:zoobank.org:act:BB4D8025-E50A-4D77-AAC4-1BE65F2AF9DC

http://species-id.net/wiki/Austrarchaea_mascordi

Figs 1C-D 7M 9F 24 42

##### Type material.

Holotype male: Coolah Tops National Park, off Gemini Road Loop, New South Wales, Australia, 31°48'59"S, 150°10'31"E, sifting elevated leaf litter under tree ferns, open eucalypt forest with ferny understory, 1159 m, 12-13.IV.2010, M. Rix, D. Harms (AMS KS114972).

Paratypes: Allotype female, same data as holotype (AMS KS114974); 1 female, same data as holotype (AMS KS114973DNA: Ar41-48-F); 3 females and 6 juveniles, same data as holotype (WAM T112566DNA: Ar41-113-J/Ar41-114-J).

##### Other material examined.

**AUSTRALIA:** New South Wales**: Coolah Tops National Park:** Breeza Lookout, 31°49'08"S, 150°11'38"E, sifting elevated leaf litter under bracken ferns, open eucalypt forest with ferny understory, 1128 m, 12-13.IV.2010, M. Rix, D. Harms, 1♂ (WAM T112565DNA: Ar40-115-M); Breeza Lookout, 31°49'17"S, 150°11'28"E, pitfall trap, 7-25.XI.2001, M. Gray, G. Milledge, H. Smith, 1♂ (AMS KS75412).

##### Etymology.

The specific epithet is a patronym in honour of the late Ramon Mascord (1913–1983), for his pioneering contributions to arachnology and natural history, and for writing two of the most influential popular books on Australian spiders (see Mascord 1970, 1980).

##### Diagnosis.

**Figure 24.**
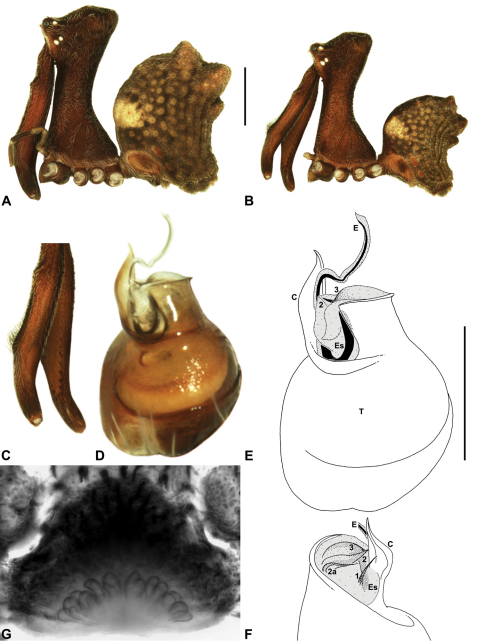
*Austrarchaea mascordi* sp. n. **A–B**, Cephalothorax and abdomen, lateral view: **A**, allotype female (AMS KS114974) from Coolah Tops National Park, New South Wales; **B**, holotype male (AMS KS114972) from Coolah Tops National Park, New South Wales. **C**, Holotype male chelicerae, lateral view, showing accessory setae. **D–F**, Holotype male pedipalp: **D–E**, bulb, retrolateral view; **F**, detail of distal tegular sclerites, prodistal view. **G**, Allotype female internal genitalia, dorsal view. C = conductor; E = embolus; Es = embolic sclerite; T = tegulum; (TS)1–3 = tegular sclerites 1–3. Scale bars: A–B = 1.0 mm; E = 0.2 mm.

*Austrarchaea mascordi* can be distinguished from all other Archaeidae from mid-eastern Australia by the relatively short, filiform tegular sclerite 1 (TS 1) (Fig. 24F) combined with the unique shape of the conductor (Figs 24D-E), which is thin and slightly twisted, with a sharply-tapered apex.

This species can also be distinguished from other genotyped taxa from mid-eastern Australia (see Fig. 3B) by the following six unique nucleotide substitutions for COI and COII (n = 4): C(348), G(468), C(651), C(957), A(967), G(1364).

##### Description.

*Holotype male*: Total length 2.83; leg I femur 2.67; F1/CL ratio 2.45. Cephalothorax dark reddish-brown; legs tan-brown with darker annulations; abdomen mottled grey-brown and beige, with darker reddish-brown dorsal scute and sclerites (Fig. 24B). Carapace tall (CH/CL ratio 2.14); 1.09 long, 2.33 high, 1.02 wide; ‘neck’ 0.54 wide; bearing two pairs of rudimentary horns; highest point of pars cephalica (HPC) approaching posterior third of ‘head’ (ratio of HPC to post-ocular length 0.62), carapace gently sloping posterior to HPC; ‘head’ not strongly elevated dorsally (post-ocular ratio 0.28) (Fig. 9F). Chelicerae with brush of accessory setae on anterior face of paturon (Fig. 24C). Abdomen 1.44 long, 1.08 wide; with three pairs of dorsal hump-like tubercles (HT 1–6); dorsal scute fused anteriorly to epigastric sclerites, extending posteriorly to first pair of hump-like tubercles; HT 3–6 each covered by separate dorsal sclerites. Unexpanded pedipalp (Figs 24D-F) with thin, sharply-tapered, slightly twisted conductor; tegular sclerite 1 (TS 1) filiform, obscured by conductor in retrolateral view; TS 2 spur-like, longer than TS 1; TS 2a sinuous, filiform, exposed distally; TS 3 embedded proximally within distal haematodocha, with arched dorsal margin and sharply-pointed apex projecting ventrally beyond retro-distal rim of tegulum.

*Allotype female*: Total length 3.28; leg I femur 2.87; F1/CL ratio 2.29. Cephalothorax dark reddish-brown; legs tan-brown with darker annulations; abdomen mottled grey-brown and beige (Fig. 24A). Carapace tall (CH/CL ratio 2.11); 1.26 long, 2.65 high, 1.13 wide; ‘neck’ 0.62 wide; bearing two pairs of rudimentary horns; highest point of pars cephalica (HPC) near middle of ‘head’ (ratio of HPC to post-ocular length 0.60), carapace gently sloping posterior to HPC; ‘head’ not strongly elevated dorsally (post-ocular ratio 0.26) (Fig. 7M). Chelicerae without accessory setae on anterior face of paturon. Abdomen 1.90 long, 1.54 wide; with three pairs of dorsal hump-like tubercles (HT 1–6). Internal genitalia with cluster of ≤ 10 variably shaped spermathecae on either side of gonopore, clusters meeting near midline of genital plate (Fig. 21G); innermost (anterior) spermathecae longest, bilobed, curved antero-laterally; other spermathecae variably pyriform, mostly straight, directed antero-laterally.

*Variation*: Males (n=3): total length 2.83–2.92; carapace length 1.09–1.10; carapace height 2.33–2.37; CH/CL ratio 2.14–2.17. Females (n=5): total length 2.97–3.49; carapace length 1.21–1.26; carapace height 2.49–2.65; CH/CL ratio 2.05–2.11.

##### Distribution and habitat.

*Austrarchaea mascordi* is known only from snow gum and wet eucalypt forest habitats in the Coolah Tops National Park, west of Scone, New South Wales (Fig. 42).

##### Conservation status.

This species is a short-range endemic taxon (Harvey 2002b), which although restricted in distribution, is abundant within the eastern Coolah Tops National Park (M. Rix, pers. obs.). It is not considered to be of conservation concern.

#### Austrarchaea smithae Blue Mountains Assassin Spider

Rix & Harvey sp. n.

urn:lsid:zoobank.org:act:A7583511-45FB-47A5-805F-1AA36E168237

http://species-id.net/wiki/Austrarchaea_smithae

Figs 7N 9G 25 43

##### Type material.

Holotype male: Blue Mountains National Park, Mount Wilson, off Mount Wilson Road, New South Wales, Australia, 33°30'56"S, 150°21'54"E, sifting elevated leaf litter under *Xanthorrhoea*, complex eucalypt forest with thick understory bordering rainforest, 948 m, 9.IV.2010, M. Rix, D. Harms (AMS KS114978).

Paratypes: Allotype female, same data as holotype (AMS KS114979); 2 females and 2 juveniles, same data as holotype (WAM T112576DNA: Ar32-116-F/Ar32-117-J/Ar32-118-J).

##### Additional material examined (of tentative identification).

**AUSTRALIA**. New South Wales**: Kanangra-Boyd National Park:** Kanangra Walls, near lookout, 33°59'08"S, 150°06'40"E, sifting elevated leaf litter under *Xanthorrhoea*, montane sclerophyll forest/heathland with thick understory, 1091 m, 10.IV.2010, M. Rix, D. Harms, 4 juveniles (WAM T112578DNA: Ar33-119-J/Ar33-120-J/Ar33-121-J); Kanangra Waterfall, near Kanangra Walls, 33°59'04"S, 150°06'33"E, sifting elevated leaf litter under ferns, streamside vegetation next to Kanangra Brook, 1069 m, 10.IV.2010, M. Rix, D. Harms, 2 juveniles (WAM T112579DNA: Ar34-122-J/Ar34-123-J).

##### Additional material (not examined).

**AUSTRALIA:** New South Wales**: Kanangra-Boyd National Park:** Kanangra Walls, near lookout, 33°59'08"S, 150°06'40"E, sifting elevated leaf litter under *Xanthorrhoea*, montane sclerophyll forest/heathland with thick understory, 6.XI.2008, H. Smith, 2 juveniles (AMS KS110500).

##### Etymology.

The specific epithet is a patronym in honour of Dr Helen Smith, for first discovering archaeids in the Blue Mountains region west of Sydney.

##### Diagnosis.

*Austrarchaea smithae* can be distinguished from all other Archaeidae from mid-eastern Australia by the unique shape of the male ‘head’ (Fig. 9G), which is strongly elevated dorsally (post-ocular ratio 0.39), with the highest point of the pars cephalica (HPC) closer to the middle of the ‘head’ than to the posterior margin of the carapace (ratio of HPC to post-ocular length < 0.75).

This species can also be distinguished from other genotyped taxa from mid-eastern Australia (see Fig. 3B) by the following 12 unique nucleotide substitutions for COI and COII (n = 3): C(42), A(45), A(81), G(120), G(288), G(456), A(708), G(758), G(1269), A(1346), C(1368), C(1590).

##### Description.

**Figure 25.**
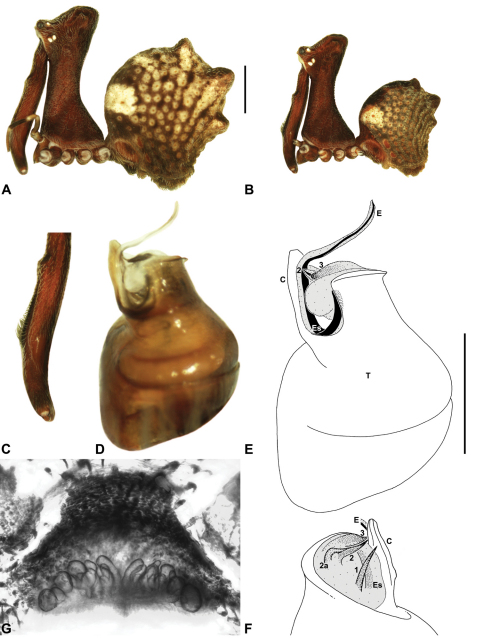
*Austrarchaea smithae* sp. n. **A–B**, Cephalothorax and abdomen, lateral view: **A**, allotype female (AMS KS114979) from Blue Mountains National Park, New South Wales; **B**, holotype male (AMS KS114978) from Blue Mountains National Park, New South Wales. **C**, Holotype male chelicerae, lateral view, showing accessory setae. **D–F**, Holotype male pedipalp: **D–E**, bulb, retrolateral view; **F**, detail of distal tegular sclerites, prodistal view. **G**, Allotype female internal genitalia, dorsal view. C = conductor; E = embolus; Es = embolic sclerite; T = tegulum; (TS)1–3 = tegular sclerites 1–3. Scale bars: A–B = 1.0 mm; E = 0.2 mm.

*Holotype male*: Total length 2.97; leg I femur 2.79; F1/CL ratio 2.48. Cephalothorax dark reddish-brown; legs tan-brown with darker annulations; abdomen mottled grey-brown and beige, with darker reddish-brown dorsal scute and sclerites (Fig. 25B). Carapace tall (CH/CL ratio 2.14); 1.13 long, 2.41 high, 1.05 wide; ‘neck’ 0.51 wide; bearing two pairs of rudimentary horns; highest point of pars cephalica (HPC) approaching posterior quarter of ‘head’ (ratio of HPC to post-ocular length 0.71), carapace gently sloping posterior to HPC; ‘head’ strongly elevated dorsally (post-ocular ratio 0.39) (Fig. 9G). Chelicerae with short brush of accessory setae on anterior face of paturon (Fig. 25C). Abdomen 1.59 long, 1.27 wide; with three pairs of dorsal hump-like tubercles (HT 1–6); dorsal scute fused anteriorly to epigastric sclerites, extending posteriorly to first pair of hump-like tubercles; HT 3–6 each covered by separate dorsal sclerites. Unexpanded pedipalp (Figs 25D-F) with thin, slightly-tapered, rectangular conductor; tegular sclerite 1 (TS 1) long, spiniform, obscured by conductor in retrolateral view; TS 2 spur-like, shorter than TS 1; TS 2a sinuous, largely obscured by TS 2; TS 3 embedded proximally within distal haematodocha, with arched dorsal margin and bluntly-pointed apex projecting ventrally beyond retro-distal rim of tegulum.

*Allotype female*: Total length 4.00; leg I femur 3.09; F1/CL ratio 2.30. Cephalothorax dark reddish-brown; legs tan-brown with darker annulations; abdomen mottled grey-brown and beige (Fig. 25A). Carapace tall (CH/CL ratio 2.13); 1.35 long, 2.87 high, 1.23 wide; ‘neck’ 0.65 wide; bearing two pairs of rudimentary horns; highest point of pars cephalica (HPC) near posterior third of ‘head’ (ratio of HPC to post-ocular length 0.66), carapace gently sloping posterior to HPC; ‘head’ moderately elevated dorsally (post-ocular ratio 0.34) (Fig. 7N). Chelicerae without accessory setae on anterior face of paturon. Abdomen 2.56 long, 1.96 wide; with three pairs of dorsal hump-like tubercles (HT 1–6). Internal genitalia with cluster of ≤ 12 variably shaped spermathecae on either side of gonopore, clusters meeting near midline of genital plate (Fig. 25G); innermost (anterior) spermathecae longest, sausage-shaped, curved antero-laterally; outermost (posterior) spermathecae bulbous; other spermathecae variably pyriform, straight, directed antero-laterally.

*Variation*: Females (n=3): total length 3.74–4.00; carapace length 1.28–1.35; carapace height 2.62–2.92; CH/CL ratio 2.04–2.19.

##### Distribution and habitat.

*Austrarchaea smithae* is known only from wet eucalypt forest habitats in the Blue Mountains National Park west of Sydney, New South Wales (Fig. 43). Numerous juvenile specimens from the Kanangra Walls Plateau (Kanangra-Boyd National Park) may also belong to this species, but possess divergent mtDNA sequences indicative of possible speciation (Fig. 3B).

##### Conservation status.

This species has an imperfectly known distribution, and although potentially restricted, appears to be relatively abundant within the World Heritage-listed Blue Mountains National Park near Mount Wilson (M. Rix, pers. obs.). It is not considered to be of conservation concern.

#### Austrarchaea helenae Illawarra Assassin Spider

Rix & Harvey sp. n.

urn:lsid:zoobank.org:act:278EE43B-C8F7-4069-9F60-4B343BB6FF6E

http://species-id.net/wiki/Austrarchaea_helenae

Figs 9I 26 44

##### Type material.

Holotype male: Macquarie Pass National Park, Macquarie Pass, New South Wales, Australia, 34°34’S, 150°39’E, pitfall trap, 12-26.IX.1999, M. Gray, G. Milledge, H. Smith (AMS KS62774).

##### Other material examined.

**AUSTRALIA:** New South Wales**: Macquarie Pass National Park:** Macquarie Pass, off Clover Hill Road, 34°34'05"S, 150°39'25"E, sifting elevated leaf litter, subtropical rainforest, 828 m, 8.IV.2010, M. Rix, D. Harms, 3 juveniles (WAM T112561DNA: Ar30-124-J/Ar30-125-J/Ar30-126-J).

##### Additional material examined (of tentative identification).

**AUSTRALIA:** New South Wales**: Morton National Park:** Barrengarry Mountain, ANIC Berlesate, rainforest, 460 m, 20.XII.1967, R. Taylor, C. Brooks, 1 juvenile (ANIC).

##### Etymology.

The specific epithet is a patronym in honour of Helen Rix, for her love of the Illawarra Escarpment, and for her hospitality to the senior author during field work in eastern Australia.

##### Diagnosis.

**Figure 26.**
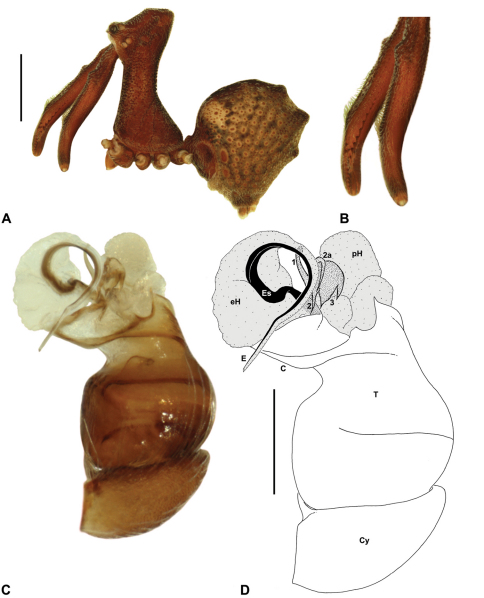
*Austrarchaea helenae* sp. n. **A–D**, Holotype male (AMS KS62774) from Macquarie Pass National Park, New South Wales: **A**, cephalothorax and abdomen, lateral view; **B**, chelicerae, lateral view, showing accessory setae; **C–D**, pedipalpal bulb (expanded), retro-ventral view. C = conductor; Cy = cymbium; E = embolus; eH = embolic portion of distal haematodocha; Es = embolic sclerite; pH = proximal portion of distal haematodocha; T = tegulum; (TS)1–3 = tegular sclerites 1–3. Scale bars: A = 1.0 mm; D = 0.2 mm.

*Austrarchaea helenae* can be distinguished from all other Archaeidae from mid-eastern Australia by the long, spiniform tegular sclerite 1 (TS 1) with a curled distal tip (Fig. 26D) combined with the bifurcate, plate-like TS 3 (Fig. 26D).

This species can also be distinguished from other genotyped taxa from mid-eastern Australia (see Fig. 3B) by the following 14 unique nucleotide substitutions for COI and COII (n = 3): G(243), A(291), T(555), C(654), C(843), G(849), C(901), T(903), T(990), A(1206), C(1209), C(1401), C(1500), C(1548).

##### Description.

*Holotype male*: Total length 3.13; leg I femur 2.69; F1/CL ratio 2.50. Cephalothorax dark reddish-brown; legs tan-brown with darker annulations; abdomen mottled grey-brown and beige, with darker reddish-brown dorsal scute and sclerites (Fig. 26A). Carapace tall (CH/CL ratio 2.01); 1.08 long, 2.17 high, 1.02 wide; ‘neck’ 0.53 wide; bearing two pairs of rudimentary horns; highest point of pars cephalica (HPC) near middle of ‘head’ (ratio of HPC to post-ocular length 0.57), carapace gently sloping and almost horizontal posterior to HPC; ‘head’ moderately elevated postero-dorsally (post-ocular ratio 0.35) (Fig. 9I). Chelicerae with short brush of accessory setae on anterior face of paturon (Fig. 26B). Abdomen 1.79 long, 1.28 wide; with three pairs of dorsal hump-like tubercles (HT 1–6); dorsal scute fused anteriorly to epigastric sclerites, extending posteriorly to first pair of hump-like tubercles; HT 3–6 each covered by separate dorsal sclerites. Expanded pedipalp (Figs 26C-D) with broadly-triangular, pointed conductor; tegular sclerite 1 (TS 1) long, spiniform, with curled distal tip, visible in retro-ventral view; TS 2 spiniform, shorter than TS 1, largely obscured by embolus and TS 3; TS 2a sinuous, filiform, exposed distally; TS 3 exposed, plate-like, overlying TS 2, with bifurcate, triangular apex directed toward proximal conductor.

*Female*: Unknown.

##### Distribution and habitat.

*Austrarchaea helenae* is known only from rainforest habitats in the Macquarie Pass National Park, on the Illawarra Escarpment of south-eastern New South Wales (Fig. 44). A juvenile specimen from Barrengarry Mountain (Morton National Park) may also belong to this species based on proximity.

##### Conservation status.

This species appears to be a rare short-range endemic taxon (Harvey 2002b), with populations on the Illawarra Escarpment **potentially threatened** by land-clearing, habitat fragmentation and fire. Much of the original rainforest of the Illawarra region has been cleared for agriculture and livestock, and only isolated fragments of forest remain.

#### Austrarchaea mcguiganae Southern Highlands Assassin Spider

Rix & Harvey sp. n.

urn:lsid:zoobank.org:act:9926676F-6892-4486-8853-77B3F767AB6A

http://species-id.net/wiki/Austrarchaea_mcguiganae

Figs 7O 9H 27 45

##### Type material.

Holotype male: Monga National Park, Link Road, New South Wales, Australia, 35°34'04"S, 149°54'14"E, 16.III.1999, L. Wilkie, R. Harris, H. Smith (AMS KS62790).

Paratypes: Allotype female, Monga National Park, off Link Road, New South Wales, Australia, 35°34'03"S, 149°54'15"E, sifting elevated leaf litter, complex eucalypt forest with thick understory near tree fern gully, 864 m, 6.IV.2010, M. Rix, D. Harms (AMS KS114975); 1 female and 5 juveniles, same data (WAM T112567DNA: Ar28-47-J/Ar28-128-J).

##### Additional material examined (of tentative identification).

**AUSTRALIA:** New South Wales**: Deua National Park:** Coondella Fire Trail, 35°58'44"S, 149°53'05"E, 11.III.1999, J. Tarnawski, S. Lassau, 1♀ (AMS KS62791). **Badja State Forest:** Badja Fire Trail, 36°07'30"S, 149°31'37"E, 13.III.1999, J. Tarnawski, S. Lassau, 1 juvenile (AMS KS62792); off Peters Road, near junction with Badja Forest Road, 36°07'38"S, 149°31'36"E, sifting elevated leaf litter, complex eucalypt forest with thick understory, 1075 m, 5.IV.2010, M. Rix, D. Harms, 14 juveniles (WAM T112577DNA: Ar27-129-J/Ar27-130-J/Ar27-131-J).

##### Etymology.

The specific epithet is a patronym in honour of the late Margaret McGuigan (1920–2010), for her love of the Southern Highlands, and for a lifetime of kindness and support to the senior author.

##### Diagnosis.

**Figure 27.**
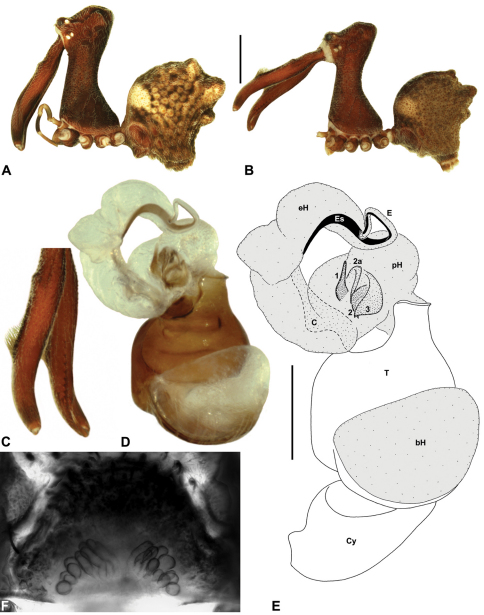
*Austrarchaea mcguiganae* sp. n. **A–B**, Cephalothorax and abdomen, lateral view: **A**, allotype female (AMS KS114975) from Monga National Park, New South Wales; **B**, holotype male (AMS KS62790) from Monga National Park, New South Wales. **C**, Holotype male chelicerae, lateral view, showing accessory setae. **D–E**, Holotype male pedipalpal bulb (fully expanded), retro-ventral view. **G**, Allotype female internal genitalia, dorsal view. bH = basal haematodocha; C = conductor; Cy = cymbium; E = embolus; eH = embolic portion of distal haematodocha; Es = embolic sclerite; pH = proximal portion of distal haematodocha; T = tegulum; (TS)1–3 = tegular sclerites 1–3. Scale bars: A–B = 1.0 mm; E = 0.2 mm.

**Figure 28.**
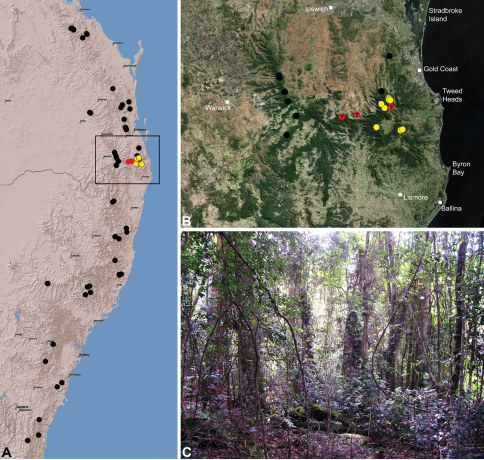
*Austrarchaea nodosa* (Forster, 1956), distribution and habitat: **A**, topographic map showing the known distribution of Archaeidae in south-eastern Queensland and eastern New South Wales, with collection localities for *A. nodosa* highlighted in yellow (red highlighted localities denote juvenile specimens of tentative identification); **B**, satellite image showing detail of inset (A); **C**, subtropical rainforest near the type locality – Binna Burra, Lamington National Park, Queensland (April 2010). Image (C) by M. Rix.

**Figure 29.**
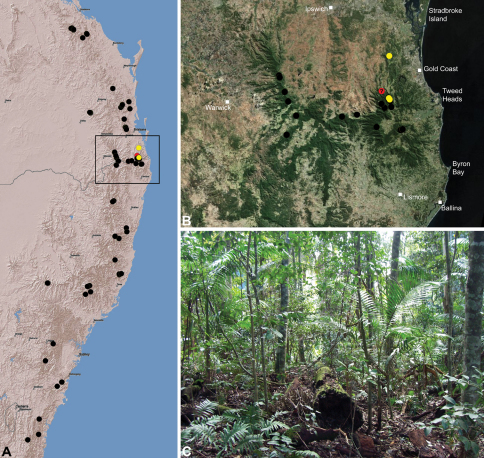
*Austrarchaea dianneae *sp. n., distribution and habitat: **A**, topographic map showing the known distribution of Archaeidae in south-eastern Queensland and eastern New South Wales, with collection localities for *A. dianneae* highlighted in yellow; **B**, satellite image showing detail of inset (A); **C**, subtropical rainforest at the type locality – Joalah Section, Tamborine National Park, Queensland (June 2009). Note the sympatric occurrence of this species with *A. nodosa* on the ‘scenic rim’ at Binna Burra, Lamington National Park. Image (C) by M. Rix.

**Figure 30.**
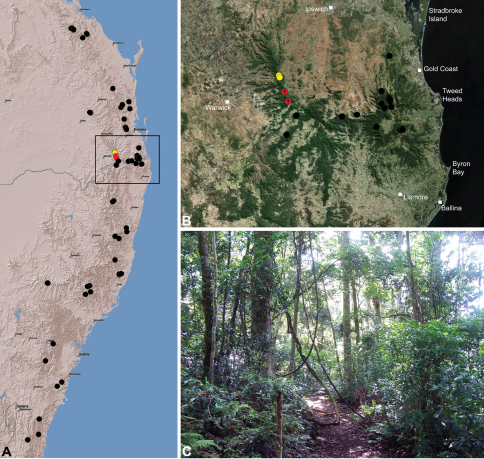
*Austrarchaea cunninghami *sp. n., distribution and habitat: **A**, topographic map showing the known distribution of Archaeidae in south-eastern Queensland and eastern New South Wales, with collection localities for *A. cunninghami* highlighted in yellow (red highlighted localities denote juvenile specimens of tentative identification); **B**, satellite image showing detail of inset (A); **C**, subtropical rainforest at the type locality – Cunningham’s Gap, Main Range National Park, Queensland (April 2010). Image (C) by M. Rix.

**Figure 31.**
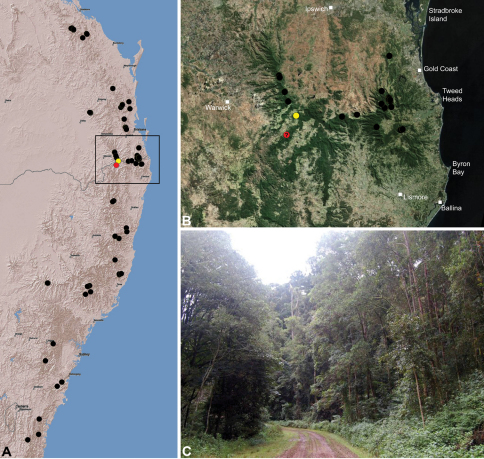
*Austrarchaea clyneae *sp. n., distribution and habitat: **A**, topographic map showing the known distribution of Archaeidae in south-eastern Queensland and eastern New South Wales, with collection localities for *A. clyneae* highlighted in yellow (red highlighted localities denote juvenile specimens of tentative identification); **B**, satellite image showing detail of inset (A); **C**, subtropical rainforest at the type locality – Mount Clunie National Park, Queensland (June 2008). Image (C) by A. Rix, used with permission.

**Figure 32.**
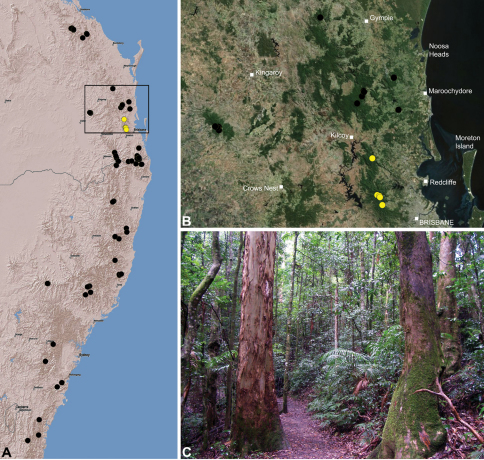
*Austrarchaea raveni *sp. n., distribution and habitat: **A**, topographic map showing the known distribution of Archaeidae in south-eastern Queensland and eastern New South Wales, with collection localities for *A. raveni* highlighted in yellow; **B**, satellite image showing detail of inset (A); **C**, subtropical rainforest near the type locality – Mount Glorious, D’Aguilar National Park, Queensland (May 2010). Image (C) by M. Rix.

**Figure 33.**
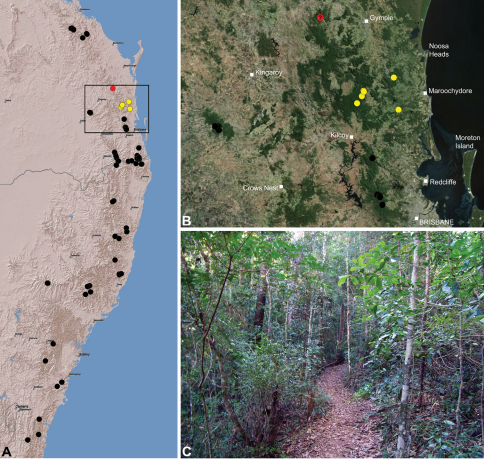
*Austrarchaea judyae *sp. n., distribution and habitat: **A**, topographic map showing the known distribution of Archaeidae in south-eastern Queensland and eastern New South Wales, with collection localities for *A. judyae* highlighted in yellow (red highlighted localities denote juvenile specimens of tentative identification); **B**, satellite image showing detail of inset (A); **C**, subtropical rainforest at the type locality – near Booloumba Creek, Conondale National Park, Queensland (May 2010). Image (C) by M. Rix.

**Figure 34.**
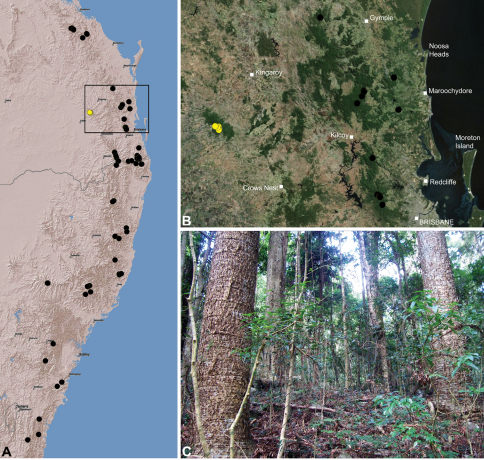
*Austrarchaea harmsi *sp. n., distribution and habitat: **A**, topographic map showing the known distribution of Archaeidae in south-eastern Queensland and eastern New South Wales, with collection localities for *A. harmsi* highlighted in yellow; **B**, satellite image showing detail of inset (A); **C**, subtropical araucarian (*Araucaria bidwillii*) rainforest at the type locality – Dandabah, Bunya Mountains National Park, Queensland (May 2010). Image (C) by M. Rix.

**Figure 35.**
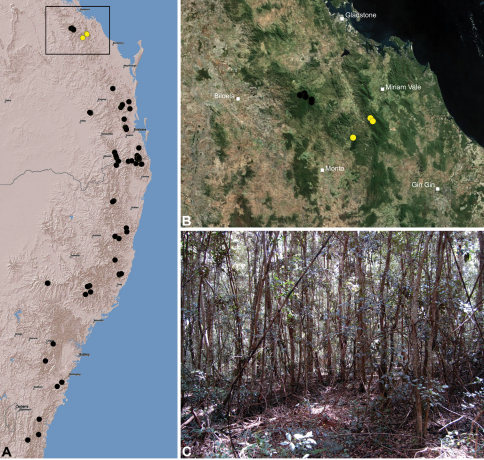
*Austrarchaea aleenae *sp. n., distribution and habitat: **A**, topographic map showing the known distribution of Archaeidae in south-eastern Queensland and eastern New South Wales, with collection localities for *A. aleenae* highlighted in yellow; **B**, satellite image showing detail of inset (A); **C**, subtropical vine rainforest at the type locality – Bulburin Forest Road, Bulburin National Park, Queensland (October 2010). Image (C) by A. Rix, used with permission.

**Figure 36.**
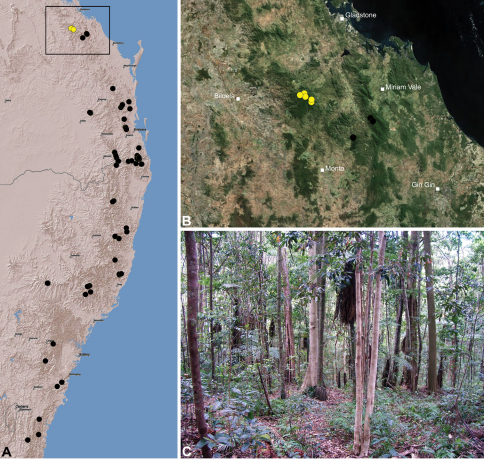
*Austrarchaea alani *sp. n., distribution and habitat: **A**, topographic map showing the known distribution of Archaeidae in south-eastern Queensland and eastern New South Wales, with collection localities for *A. alani* highlighted in yellow; **B**, satellite image showing detail of inset (A); **C**, subtropical rainforest near the type locality – Tablelands Road, Kroombit Tops National Park, Queensland (October 2010). Image (C) by M. Rix.

**Figure 37.**
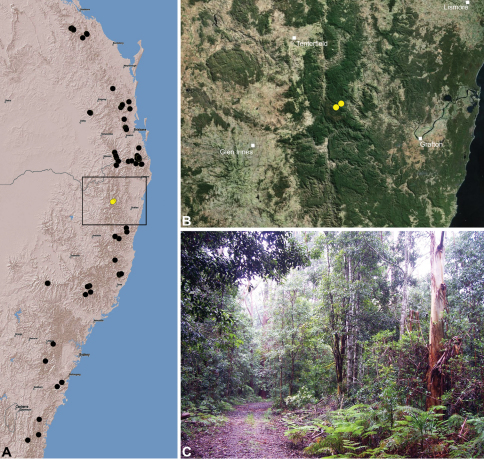
*Austrarchaea monteithi *sp. n., distribution and habitat: **A**, topographic map showing the known distribution of Archaeidae in south-eastern Queensland and eastern New South Wales, with collection localities for *A. monteithi* highlighted in yellow; **B**, satellite image showing detail of inset (A); **C**, subtropical rainforest at the type locality – near Richardsons Creek, Gibralter Range National Park, New South Wales (April 2010). Image (C) by M. Rix.

**Figure 38.**
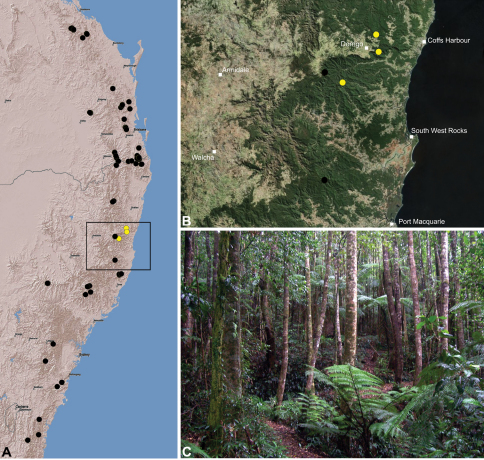
*Austrarchaea christopheri* sp. n., distribution and habitat: **A**, topographic map showing the known distribution of Archaeidae in south-eastern Queensland and eastern New South Wales, with collection localities for *A. christopheri* highlighted in yellow; **B**, satellite image showing detail of inset (A); **C**, subtropical rainforest at the type locality – The Never Never, Dorrigo National Park, New South Wales (April 2010). Image (C) by M. Rix.

**Figure 39.**
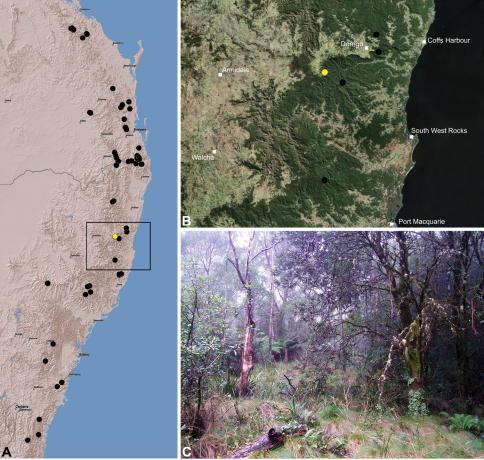
*Austrarchaea platnickorum* sp. n., distribution and habitat: **A**, topographic map showing the known distribution of Archaeidae in south-eastern Queensland and eastern New South Wales, with collection localities for *A. platnickorum* highlighted in yellow; **B**, satellite image showing detail of inset (A); **C**, snow gum woodland adjacent to cool-temperate *Nothofagus moorei* rainforest at the type locality – Banksia Point, New England National Park, New South Wales (April 2010). Image (C) by M. Rix.

**Figure 40.**
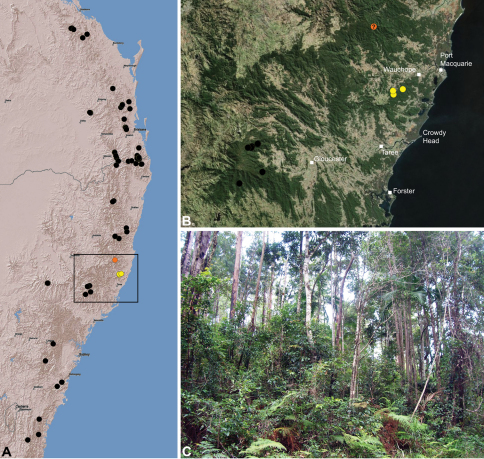
*Austrarchaea binfordae* sp. n., distribution and habitat: **A**, topographic map showing the known distribution of Archaeidae in south-eastern Queensland and eastern New South Wales, with collection localities for *A. binfordae* highlighted in yellow (orange localities denote genotyped juvenile specimens of tentative identification); **B**, satellite image showing detail of inset (A); **C**, lowland subtropical rainforest at the type locality – McLeods Creek Road, Kerewong State Forest, New South Wales (April 2010). Image (C) by M. Rix.

**Figure 41.**
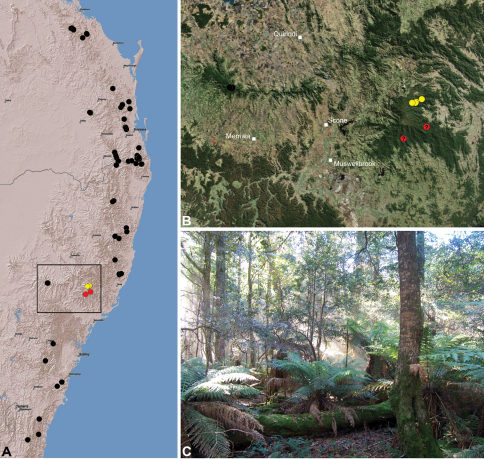
*Austrarchaea milledgei* sp. n., distribution and habitat: **A**, topographic map showing the known distribution of Archaeidae in south-eastern Queensland and eastern New South Wales, with collection localities for *A. milledgei* highlighted in yellow (red highlighted localities denote specimens of tentative identification); **B**, satellite image showing detail of inset (A); **C**, cool-temperate *Nothofagus moorei* rainforest near the type locality – Barrington Tops National Park, New South Wales (April 2010). Image (C) by M. Rix.

**Figure 42.**
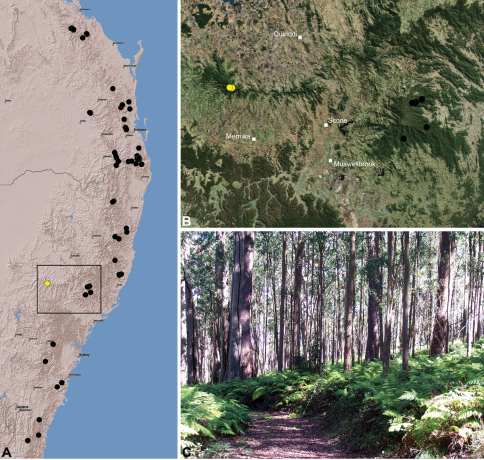
*Austrarchaea mascordi* sp. n., distribution and habitat: **A**, topographic map showing the known distribution of Archaeidae in south-eastern Queensland and eastern New South Wales, with collection localities for *A. mascordi* highlighted in yellow; **B**, satellite image showing detail of inset (A); **C**, open eucalypt forest near the type locality – Breeza Lookout, Coolah Tops National Park, New South Wales (April 2010). Image (C) by M. Rix.

**Figure 43.**
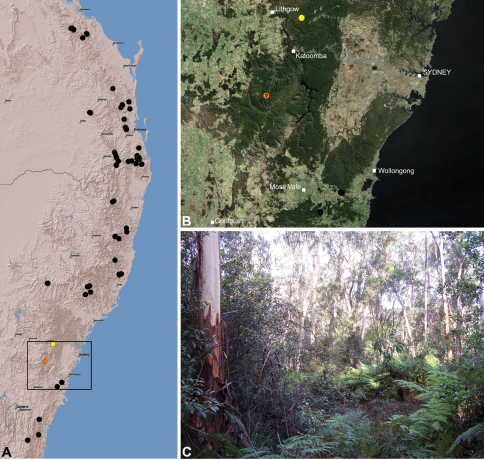
*Austrarchaea smithae* sp. n., distribution and habitat: **A**, topographic map showing the known distribution of Archaeidae in south-eastern Queensland and eastern New South Wales, with collection localities for *A. smithae* highlighted in yellow (orange localities denote genotyped juvenile specimens of tentative identification); **B**, satellite image showing detail of inset (A); **C**, wet eucalypt forest at the type locality – Mount Wilson, Blue Mountains National Park, New South Wales (April 2010). Image (C) by M. Rix.

**Figure 44.**
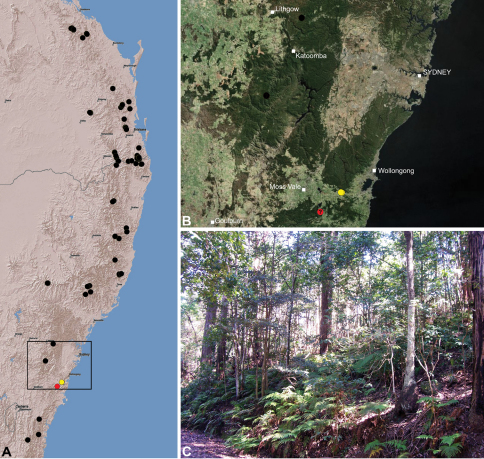
*Austrarchaea helenae* sp. n., distribution and habitat: **A**, topographic map showing the known distribution of Archaeidae in south-eastern Queensland and eastern New South Wales, with collection localities for *A. helenae* highlighted in yellow (red highlighted localities denote juvenile specimens of tentative identification); **B**, satellite image showing detail of inset (A); **C**, subtropical rainforest at the type locality – Macquarie Pass, Macquarie Pass National Park, New South Wales (April 2010). Image (C) by M. Rix.

*Austrarchaea mcguiganae* can be distinguished from all other Archaeidae from mid-eastern Australia by the relatively short, rod-like, proximally-widened tegular sclerite 1 (TS 1) (Figs 27D-E) combined with the long brush of accessory setae on the male chelicerae (Fig. 27C).

This species can also be distinguished from other genotyped taxa from mid-eastern Australia (see Fig. 3B) by the following seven unique nucleotide substitutions for COI and COII (n = 2): T(57), C(144), T(156), G(465), G(504), C(798), G(1548).

##### Description.

*Holotype male*: Total length 3.17; leg I femur 2.81; F1/CL ratio 2.49. Cephalothorax dark reddish-brown; legs tan-brown with darker annulations; abdomen mottled grey-brown and beige, with darker reddish-brown dorsal scute and sclerites (Fig. 27B). Carapace tall (CH/CL ratio 2.00); 1.13 long, 2.26 high, 1.06 wide; ‘neck’ 0.54 wide; bearing two pairs of rudimentary horns; highest point of pars cephalica (HPC) near posterior third of ‘head’ (ratio of HPC to post-ocular length 0.63), carapace gently sloping posterior to HPC; ‘head’ moderately elevated postero-dorsally (post-ocular ratio 0.32) (Fig. 9H). Chelicerae with long brush of accessory setae on anterior face of paturon (Fig. 27C). Abdomen 1.64 long, 1.10 wide; with three pairs of dorsal hump-like tubercles (HT 1–6); dorsal scute fused anteriorly to epigastric sclerites, extending posteriorly to first pair of hump-like tubercles; HT 3–6 each covered by separate dorsal sclerites. Fully expanded pedipalp (Figs 27D-E) with conductor hinged to, and obscured by, embolic haematodocha; tegular sclerite 1 (TS 1) rod-like, bluntly-pointed, visible in retro-ventral view; TS 2 spur-like, slightly longer than TS 1, largely obscured by TS 3; TS 2a sinuous, filiform, exposed distally; TS 3 exposed, plate-like, overlying TS 2, with curved, triangular apex directed toward proximal conductor.

*Allotype female*: Total length 3.49; leg I femur 2.86; F1/CL ratio 2.28. Cephalothorax dark reddish-brown; legs tan-brown with darker annulations; abdomen mottled grey-brown and beige (Fig. 27A). Carapace tall (CH/CL ratio 2.04); 1.26 long, 2.56 high, 1.18 wide; ‘neck’ 0.64 wide; bearing two pairs of rudimentary horns; highest point of pars cephalica (HPC) near middle of ‘head’ (ratio of HPC to post-ocular length 0.60), carapace gently sloping posterior to HPC; ‘head’ moderately elevated dorsally (post-ocular ratio 0.33) (Fig. 7O). Chelicerae without accessory setae on anterior face of paturon. Abdomen 2.00 long, 1.41 wide; with three pairs of dorsal hump-like tubercles (HT 1–6). Internal genitalia with cluster of ≤ 12 variably shaped spermathecae on either side of gonopore, clusters meeting near midline of genital plate (Fig. 27F); innermost (anterior) spermathecae longest, sausage-shaped, curved antero-laterally; other spermathecae variably aciniform, straight, directed antero-laterally.

*Variation*: Females (n=2): total length 3.38–3.49; carapace length 1.26 (invariable); carapace height 2.56–2.62; CH/CL ratio 2.04–2.08.

##### Distribution and habitat.

**Figure 45.**
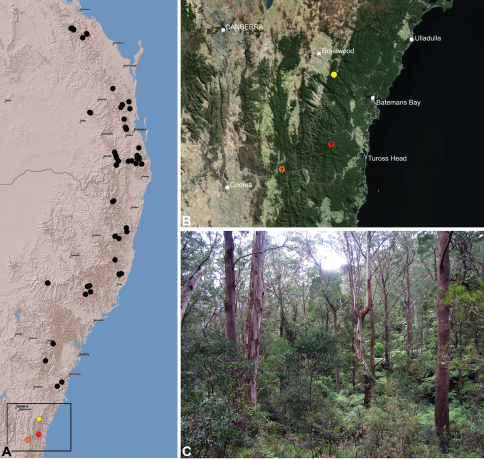
*Austrarchaea mcguiganae* sp. n., distribution and habitat: **A**, topographic map showing the known distribution of Archaeidae in south-eastern Queensland and eastern New South Wales, with collection localities for *A. mcguiganae* highlighted in yellow (red highlighted localities denote specimens of tentative identification; orange highlighted localities denote genotyped juvenile specimens of tentative identification); **B**, satellite image showing detail of inset (A); **C**, wet eucalypt forest at the type locality – Link Road, Monga National Park, New South Wales (April 2010). Image (C) by M. Rix.

*Austrarchaea mcguiganae* is known only from mesic closed forest habitats in the Monga National Park of southern New South Wales (Fig. 45). A female specimen from Deua National Park may also belong to this species based on proximity, and numerous juvenile specimens from the Badja State Forest possess divergent mtDNA sequences indicative of possible speciation (Fig. 3B).

##### Conservation status.

This species appears to be a short-range endemic taxon (Harvey 2002b), which although potentially restricted in distribution, is abundant within the Monga National Park near Link Road (M. Rix, pers. obs.). It is not considered to be of conservation concern.

## Supplementary Material

XML Treatment for Austrarchaea

XML Treatment for Austrarchaea nodosa McPherson Range Assassin Spider

XML Treatment for Austrarchaea dianneae Gold Coast Hinterland Assassin Spider

XML Treatment for Austrarchaea cunninghami Main Range Assassin Spider

XML Treatment for Austrarchaea clyneae Mount Clunie Assassin Spider

XML Treatment for Austrarchaea raveni D’Aguilar Range Assassin Spider

XML Treatment for Austrarchaea judyae Sunshine Hinterland Assassin Spider

XML Treatment for Austrarchaea harmsi Bunya Mountains Assassin Spider

XML Treatment for Austrarchaea aleenae Bulburin Assassin Spider

XML Treatment for Austrarchaea alani Kroombit Tops Assassin Spider

XML Treatment for Austrarchaea monteithi Gibraltar Range Assassin Spider

XML Treatment for Austrarchaea christopheri Dorrigo Assassin Spider

XML Treatment for Austrarchaea platnickorum New England Assassin Spider

XML Treatment for Austrarchaea binfordae Kerewong Assassin Spider

XML Treatment for Austrarchaea milledgei Barrington Tops Assassin Spider

XML Treatment for Austrarchaea mascordi Coolah Tops Assassin Spider

XML Treatment for Austrarchaea smithae Blue Mountains Assassin Spider

XML Treatment for Austrarchaea helenae Illawarra Assassin Spider

XML Treatment for Austrarchaea mcguiganae Southern Highlands Assassin Spider

## Appendix I

Supplementary nexus file. (doi: 10.3897/zookeys.123.1448.app) File format: NXS

Explanation note: Nexus file of aligned COI and COII nucleotide sequences (see Table 2) with Bayesian command block, used for the molecular phylogenetic analysis (see Fig. 3).

Copyright notice: This dataset is made available under the Open Database License (http://opendatacommons.org/licenses/odbl/1.0/). The Open Database License (ODbL) is a license agreement intended to allow users to freely share, modify, and use this Dataset while maintaining this same freedom for others, provided that the original source and author(s) are credited.

## References

[B1] Butler LSG (1929) Studies in Australian spiders, No. 1. Proceedings of the Royal Society of Victoria 42: 41-52.

[B2] Cambridge OP (1881) On some new genera and species of Araneidea. Proceedings of the Zoological Society of London 1881: 765-775.

[B3] Cook LG Edwards RD Crisp MD Hardy NB (2010) Need morphology always be required for new species descriptions? Invertebrate Systematics 24: 322–326. 10.1071/IS10011

[B4] Forster RR (1956) A new spider of the genus *Archaea* from Australia. Memoirs of the Queensland Museum 13: 151-154.

[B5] Forster RR Platnick NI (1984) A review of the archaeid spiders and their relatives, with notes on the limits of the superfamily Palpimanoidea (Arachnida, Araneae). Bulletin of the American Museum of Natural History 178: 1-106.

[B6] Forster RR Platnick NI Gray MR (1987) A review of the spider superfamilies Hypochiloidea and Austrochiloidea (Araneae, Araneomorphae). Bulletin of the American Museum of Natural History 185: 1-116.

[B7] Griswold CE Ramírez MJ Coddington JA Platnick NI (2005) Atlas of phylogenetic data for entelegyne spiders (Araneae: Araneomorphae: Entelegynae) with comments on their phylogeny. Proceedings of the California Academy of Sciences 56(Supplement II): 1–324.

[B8] Harvey MS (2002a) A new species of *Austrarchaea* (Araneae: Archaeidae) from Western Australia. Records of the Western Australian Museum 21: 35-37.

[B9] Harvey MS (2002b) Short-range endemism among the Australian fauna: some examples from non-marine environments. Invertebrate Systematics 16: 555-570. 10.1071/IS02009

[B10] Harvey MS Berry O Edward KL Humphreys G (2008) Molecular and morphological systematics of hypogean schizomids (Schizomida: Hubbardiidae) in semiarid Australia. Invertebrate Systematics 22: 167-194. 10.1071/IS07026

[B11] Higgins ET Petterd WF (1883) Description of a new cave-inhabiting spider, together with notes on mammalian remains from a recently discovered cave in the Chudleigh district. Papers and Proceedings of the Royal Society of Tasmania 1882: 191-192.

[B12] Huelsenbeck JP Ronquist F (2001) MRBAYES: Bayesian inference of phylogenetic trees. Bioinformatics 17: 754-755. 10.1093/bioinformatics/17.8.754 11524383

[B13] Koch CL Berendt GC (1854) Die im Bernstein befindlichen Crustaceen, Myriapoden, Arachniden und Apteren der Vorweld. In: Berendt GC (Ed) Die im Bernstein befindlichen Organischen Reste der Vorweld, Berlin, 1–124.

[B14] Legendre R (1961) Études sur les *Archaea* (Aranéides). II. La capture des proies et la prise de nourriture. Bulletin de la Société zoologique de France 86: 316-319.

[B15] Lotz LN (1996) Afrotropical Archaeidae (Araneae): 1. New species of *Afrarchaea* with notes on *Afrarchaea godfreyi* (Hewitt, 1919). Navorsinge van die Nasionale Museum, Bloemfontein 12: 141-160.

[B16] Lotz LN (2003) Afrotropical Archaeidae: 2. New species of the genera *Archaea* and *Afrarchaea* (Arachnida: Araneae). Navorsinge van die Nasionale Museum, Bloemfontein 19: 221-240.

[B17] Lotz LN (2006) Afrotropical Archaeidae: 3. The female of *Eriauchenius cornutus* and new species of *Afrarchaea* (Arachnida: Araneae) from South Africa. Navorsinge van die Nasionale Museum, Bloemfontein 22: 113-127.

[B18] Main BY (1995) Additional records of the Gondwanan spider *Austrarchaea* from southwestern Australia. Western Australian Naturalist 20: 151-154.

[B19] Mascord R (1970) Australian Spiders in Colour. Reed Books, Sydney, 112 pp.

[B20] Mascord, R. (1980) Spiders of Australia, A Field Guide. Reed Books, Sydney, 128 pp.

[B21] Mello-Leitão CFD (1945) Arañas de Misiones, Corrientes y Entre Ríos. Revista del Museo de La Plata (Nueva Serie, Zoología) 4: 213-302.

[B22] Penney D (2003) *Afrarchaea grimaldii*, a new species of Archaeidae (Araneae) in Cretaceous Burmese amber. Journal of Arachnology 31: 122-130. 10.1636/0161-8202(2003)031[0122:AGANSO]2.0.CO;2

[B23] Platnick NI (1991a) On Malagasy *Archaea* (Araneae, Archaeidae). Journal of African Zoology 105: 135-140.

[B24] Platnick NI (1991b) On Western Australian *Austrarchaea* (Araneae, Archaeidae). Bulletin of the British Arachnological Society 8: 259-261.

[B25] Platnick NI (2011) The World Spider Catalog, Version 11.5. American Museum of Natural History, New York http://research.amnh.org/entomology/spiders/catalog/ [accessed 18.IV.2011]

[B26] Posada D Crandall KA (1998) Modeltest: testing the model of DNA substitution. Bioinformatics 14: 817-818. 10.1093/bioinformatics/14.9.817 9918953

[B27] Rambaut A Drummond AJ (2009) Tracer Version 1.5. http://beast.bio.ed.ac.uk/Tracer [accessed 19.VII.2011]

[B28] Rix MG Harvey MS (2008) A survey of populations of Main’s Assassin Spider (*Austrarchaea mainae*) near Albany. Unpublished report to Verve Energy and the Water Corporation, Western Australian Museum, Perth, 1–9.

[B29] Rix MG Harvey MS (2010) The first pararchaeid spider (Araneae: Pararchaeidae) from New Caledonia, with a discussion on spinneret spigots and egg sac morphology in *Ozarchaea*. Zootaxa 2412: 27-40.

[B30] Rix MG Harvey MS Roberts JD (2008) Molecular phylogenetics of the spider family Micropholcommatidae (Arachnida: Araneae) using nuclear rRNA genes (18S and 28S). Molecular Phylogenetics and Evolution 46: 1031-1048. 10.1016/j.ympev.2007.11.001 18162409

[B31] Ronquist F Huelsenbeck JP (2003) MrBayes 3: Bayesian phylogenetic inference under mixed models. Bioinformatics 19: 1572-1574. 10.1093/bioinformatics/btg180 12912839

[B32] Schütt K (2002) The limits and phylogeny of the Araneoidea (Arachnida, Araneae). PhD thesis, Humboldt-Universität zu Berlin, Berlin.

[B33] Selden PA Diying H Dong R (2008) Palpimanoid spiders from the Jurassic of China. Journal of Arachnology 36: 306-321. 10.1636/CA07-106.1

[B34] Simon C Frati F Beckenbach A Crespi B Liu H Flook P (1994) Evolution, weighting, and phylogenetic utility of mitochondrial gene sequences and a compilation of conserved polymerase chain reaction primers. Annals of the Entomological Society of America 87: 651-701.

[B35] Specht RL (1981) Major vegetation formations in Australia. In: Keast A (Ed). Ecological Biogeography of Australia, Dr. W. Junk bv Publishers, The Hague: 605-694.

[B36] Thompson JD Gibson TJ Plewniak F Jeanmougin F Higgins DG (1997) The CLUSTAL_X windows interface: flexible strategies for multiple sequence alignment aided by quality analysis tools. Nucleic Acids Research 25: 4876-4882. 10.1093/nar/25.24.4876 9396791 PMC147148

[B37] Webb LJ Tracey JG (1981) Australian rainforests: patterns and change. In: Keast A (Ed). Ecological Biogeography of Australia, Dr. W. Junk bv Publishers, The Hague: 605-694.

[B38] Wood H (2008) A revision of the assassin spiders of the *Eriauchenius* gracilicollis group, a clade of spiders endemic to Madagascar (Araneae: Archaeidae). Zoological Journal of the Linnean Society 152: 255-296. 10.1111/j.1096-3642.2007.00359.x

[B39] Wood HM Griswold CE Gillespie RG Elias DO (2010) Archaeid and mecysmaucheniid spiders and their relatives (Araneae: Archaeidae, Mecysmaucheniidae): phylogeny, biogeography and evolution of the carapace morphology. In: Żabka M (Ed) 18th International Congress of Arachnology, Book of Abstracts, July 2010. Akademia Podlaska, Siedlce, 480.

[B40] Wood HM Griswold CE Spicer GS (2007) Phylogenetic relationships within an endemic group of Malagasy ‘assassin spiders’ (Araneae, Archaeidae): ancestral character reconstruction, convergent evolution and biogeography. Molecular Phylogenetics and Evolution 45: 612-619. 10.1016/j.ympev.2007.07.012 17869131

